# Nanomedicines Against Mitochondrial Dysfunction‐Induced Metabolic Diseases

**DOI:** 10.1002/advs.202514522

**Published:** 2025-11-21

**Authors:** Ke Xu, Leyi Wang, Tao Lv, Chen Jin, Qiong Wu, Yongjie Zhou, Gang Xu, Zhenyu Duan, Kui Luo, Jiayin Yang

**Affiliations:** ^1^ Liver Transplant Center Organ Transplant Center Department of General Surgery Department of Radiology Huaxi MR Research Center (HMRRC) Institution of Radiology and Medical Imaging Frontiers Science Center for Disease‐Related Molecular Network State Key Laboratory of Biotherapy West China Hospital Sichuan University Chengdu 610041 China; ^2^ Psychoradiology Key Laboratory of Sichuan Province and Research Unit of Psychoradiology Chinese Academy of Medical Sciences Chengdu 610041 China; ^3^ Laboratory of Liver Transplantation Institute of Organ Transplantation Key Laboratory of Transplant Engineering and Immunology NHC West China Hospital of Sichuan University Chengdu 610041 China

**Keywords:** metabolic diseases, mitochondrial dysfunction, nanomedicine, targeted delivery

## Abstract

Mitochondrial dysfunction is a common pathology for metabolic diseases such as obesity, diabetes, non‐alcoholic fatty liver disease, atherosclerosis, Alzheimer's disease (AD), and Parkinson's disease (PD). Nanomedicines provide a revolutionary strategy for mitochondrial function repair. They can realize targeted delivery, responsive release, and integration of multimodal therapies through nanotechnology and engineering and overcome limitations of traditional therapeutic methods, such as insufficient targeting, low bioavailability, and toxic side effects. In this article, the pathological characteristics of mitochondria are first introduced, and the relationship between mitochondrial dysfunction and metabolic diseases are illustrated. Structural features and design strategies of nanomedicines targeting mitochondrial dysfunction are summarized, with particular elaboration on targeting strategies and response mechanisms for diseased organs and subcellular organelles such as the liver, adipose tissue, atherosclerotic plaques, the brain, and mitochondria. The application and clinical translation of nanomedicines in obesity, atherosclerosis, diabetes, non‐alcoholic fatty liver disease (NAFLD), and brain metabolic disorders are detailed. This article is concluded with a summary and outlook of the current research status, challenges, and future development directions.

## Graphical abstract

Mitochondrial dysfunction is central to metabolic diseases. Nanomedicine offers transformative approaches for restoring function via targeted delivery, responsive release, and multimodal therapy. This review outlines the pathological basis, nanocarrier design, organelle‐specific targeting, recent advances, and future clinical translation challenges.

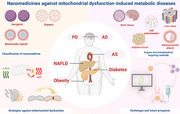

## 1 Introduction

Mitochondria, the energy provider driving diverse life activities, are regarded as the “powerhouses” of the cell, facilitating the conversion of nutrients into adenosine triphosphate (ATP), the energy currency directly usable by cells, through a process known as oxidative phosphorylation^[^ ^1^ ^]^ This process is vital for sustaining basic cellular metabolism and regulating essential physiological processes, including calcium homeostasis, apoptosis, and inflammatory responses.^[^ ^2^, ^3^ ^]^ Each mitochondrion possesses its own genome and double‐membrane structure, thereby establishing a semi‐autonomous organelle as a nexus between gene regulation and metabolic networks.^[^ ^4^ ^]^ Statistical evidence indicates that each cardiomyocyte in the human body contains up to 20 000 mitochondria, with an oxidative phosphorylation efficiency of 80%. This capacity is sufficient to meet the daily ATP consumption demand of ≈500 kcal kg^−1^ in the heart.^[^ ^5^ ^]^ The malfunction of this delicate energy factory instigates a sequence of events that can be likened to a domino effect, resulting in oxidative stress storms, ion homeostasis imbalances, and defects in mitophagy. This cascade of events ultimately culminates in cell death and organ failure.^[^ ^6^ ^]^

In the domain of metabolic diseases, mitochondrial dysfunction has emerged as a prevalent pathology underlying obesity, diabetes, non‐alcoholic fatty liver diseases, atherosclerosis, and other maladies.^[^ ^7^ ^]^ To illustrate this phenomenon, consider an example of the liver. Lipid overload has been demonstrated to induce enhanced mitochondrial uncoupling, leading to a decrease in the fatty acid oxidation efficiency and a subsequent reactive oxygen species (ROS) burst.^[^ ^8^ ^]^ This sequence of events establishes a malicious cycle of “lipotoxicity‐oxidative stress.” This pathological change has been demonstrated to drive the progression of simple fatty liver diseases to non‐alcoholic steatohepatitis.^[^ ^9^ ^]^ Furthermore, it has been shown to exert a systemic metabolic disorder network effect by releasing pro‐inflammatory factors and free fatty acids.^[^ ^10^ ^]^ Conversely, an imbalance of mitochondrial fusion‐fission dynamics in skeletal muscle results in impaired muscle fiber type conversion, thereby reducing the capacity of insulin‐stimulated glucose uptake.^[^ ^11^ ^]^ This phenomenon is a critical component of the pathogenesis of type 2 diabetes.^[^ ^12^ ^]^ It is noteworthy that a bidirectional regulatory relationship exists between mitochondrial dysfunction and metabolic diseases.^[^ ^13^ ^]^ For instance, the reduced activity of mitochondrial complex IV in pancreatic β‐cells has been shown to result in diminished insulin secretion. In addition, chronic hyperglycemia has been observed to intensify mitochondrial DNA damage through advanced glycation end products, thereby establishing an irreversible pathological cycle.^[^ ^14^ ^]^

Despite notable advancements achieved at the mechanistic level by traditional antioxidants (e.g., coenzyme Q10 and idebenone) and mitochondrial protective drugs (e.g., cyclosporine A), their clinical translation continues to face numerous challenges. First, inefficiency in mitochondrially targeted delivery results in drug accumulation in non‐target organs.^[^ ^15^ ^]^ Second, the utilization of single‐target interventions to address multidimensional pathological characteristics of mitochondrial dysfunction presents significant challenges. The elimination of ROS alone is insufficient to rectify mitochondrial biosynthesis disorders, while excessive inhibition of the mitochondrial permeability transition pore (mPTP) may disrupt the regulatory processes that govern normal cell apoptosis.^[^ ^16^ ^]^ Furthermore, current available pharmaceuticals exhibit a lack of temporal and spatial specificity, impeding the ability to achieve precise intervention during the critical window of disease progression.^[^ ^17^ ^]^

The advent of nanomedicine has allowed for transformative resolutions for the aforementioned challenges. The development of multi‐functional nanocarriers has endowed nanomedicine with spatiotemporal precision in the control of mitochondrial targeting.^[^ ^18^ ^]^ On the one hand, nanomedicine has the capacity to overcome numerous biological barriers due to its size (10–200 nm) and surface functional groups (e.g., ligand‐receptor and cationic triphenylphosphine (TPP)) to achieve high‐efficiency enrichment of drugs in organs/tissues and the mitochondrial matrix.^[^ ^19^ ^]^ Conversely, the strategic design of pH/ROS‐sensitive chemical bonds enables precise regulation of the spatiotemporal release of nanomedicine within the mitochondria under pathological conditions.^[^ ^20^ ^]^ Of particular significance is the ability of nanocarriers to address multifaceted pathological characteristics of mitochondrial dysfunction, thereby providing a distinctive platform for the implementation of multimodal synergistic therapy.^[^ ^21^ ^]^ The primary advantage of this approach is the capacity of nanocarriers to effectively co‐deliver or sequentially deliver therapeutic drugs with divergent mechanisms of action (e.g., antioxidants that clear reactive oxygen species, activators that promote mitochondrial biosynthesis, modulators that regulate autophagy, and/or inhibitors that suppress mPTP opening) to the site of dysfunctional mitochondria.^[^ ^22^ ^]^ This precise drug combination strategy utilizes nanomedicine to target multiple aspects of mitochondrial dysfunction (e.g., oxidative stress, impaired biosynthesis, kinetic imbalance, and/or defective quality control) in a simultaneous or synergistic manner. This approach produces synergistic effects to overcome the limitations of traditional monotherapy.^[^ ^23^ ^]^ It has been demonstrated that mitochondrially targeted nanodrugs allow for precise intervention of mitochondrial dysfunction. These nanodrugs have the potential to enhance treatment outcomes and clinical prognosis for mitochondrial dysfunction‐related metabolic diseases, including non‐alcoholic fatty liver disease (NAFLD) and atherosclerosis.^[^ ^24^ ^]^

**Figure 1:**
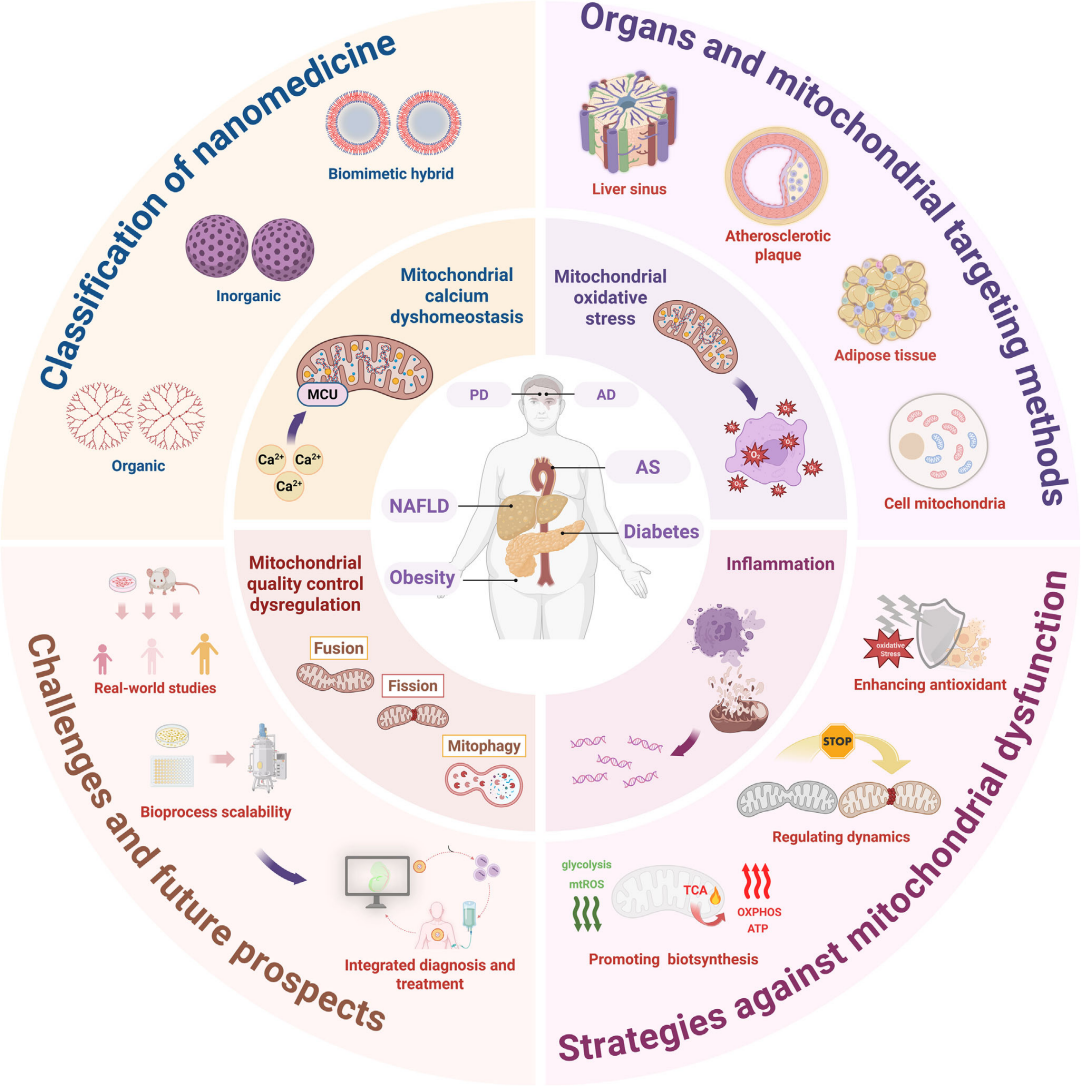
An overview of nanomedicine targeting mitochondria for the treatment of metabolic diseases. A knowledge framework for disease phenotypes and mitochondrial pathological mechanisms is systematically integrated into nanomedicine design and intervention strategies, as well as current challenges in translation and future development directions. Created in https://BioRender.com.

In light of these findings, the present study offers a systematic review of therapeutic advances and mechanistic breakthroughs in nanomedicine for metabolic diseases associated with mitochondrial dysfunction (**Figure** **1**). First, the core pathological mechanism of mitochondrial dysfunction is thoroughly analyzed, including oxidative stress, calcium homeostasis imbalance, inflammatory cascades, and quality control failure. Their molecular networks driving metabolic diseases such as obesity, diabetes, NAFLD, atherosclerosis, and brain metabolic disorders like Alzheimer's disease (AD) and Parkinson's disease (PD), are elucidated. Second, engineering design principles of inorganic, organic, and biomimetic hybrid nanocarriers are discussed, with a focus on elaborating strategies for their targeting and smart response. Treatment and intervention in mitochondrial dysfunction by nanodrugs through promoting mitochondrial biogenesis, regulating mitochondrial dynamics, and enhancing antioxidant defense are systematically summarized. Subsequently, examples are provided to illustrate the application of specific nanomedicine in improving treatment effects on key metabolic diseases, including obesity, atherosclerosis, diabetes, non‐alcoholic fatty liver disease, and neurodegenerative conditions. Moreover, innovative solutions through interdisciplinary collaboration are proposed to address current challenges in clinical translation of nanomedicine, with the aim of establishing a comprehensive knowledge framework that spans from pathological foundations of mitochondrial dysfunction to clinical translation of nanotherapy. This framework is established to provide a robust theoretical foundation and forward‐looking technological development for precise intervention in metabolic diseases.

## 2 Mitochondrial Pathology

Mitochondria are responsible for the precise regulation of cellular energy metabolism and fate decisions through their core function‐oxidative phosphorylation. The electron transport chain (ETC) employs NADH and FADH2, which are produced by the tricarboxylic acid cycle, fatty acid β‐oxidation, and amino acid metabolism, to establish a proton gradient (Δp) across the inner membrane. This process drives ATP synthesis.^[^ ^25^, ^26^ ^]^ Mitochondria, the key metabolic hub within cells, coordinate multiple metabolic pathways, including fatty acid oxidation, the tricarboxylic acid cycle, and ketogenesis. They are also involved in calcium signaling, proliferation, and apoptosis regulation.^[^ ^27^ ^]^ Furthermore, mitochondria constitute an information processing system (MIPS) through mechanisms including dynamic network remodeling (fusion/fission), biogenesis, and autophagy, collectively sustaining cellular homeostasis.^[^ ^28^ ^]^ In this context, an imbalance in mitochondrial homeostasis is the primary pathological mechanism underlying various diseases, particularly metabolic diseases such as obesity, diabetes, NAFLD, NASH, as well as atherosclerosis.^[^ ^7^ ^]^

**Figure 2:**
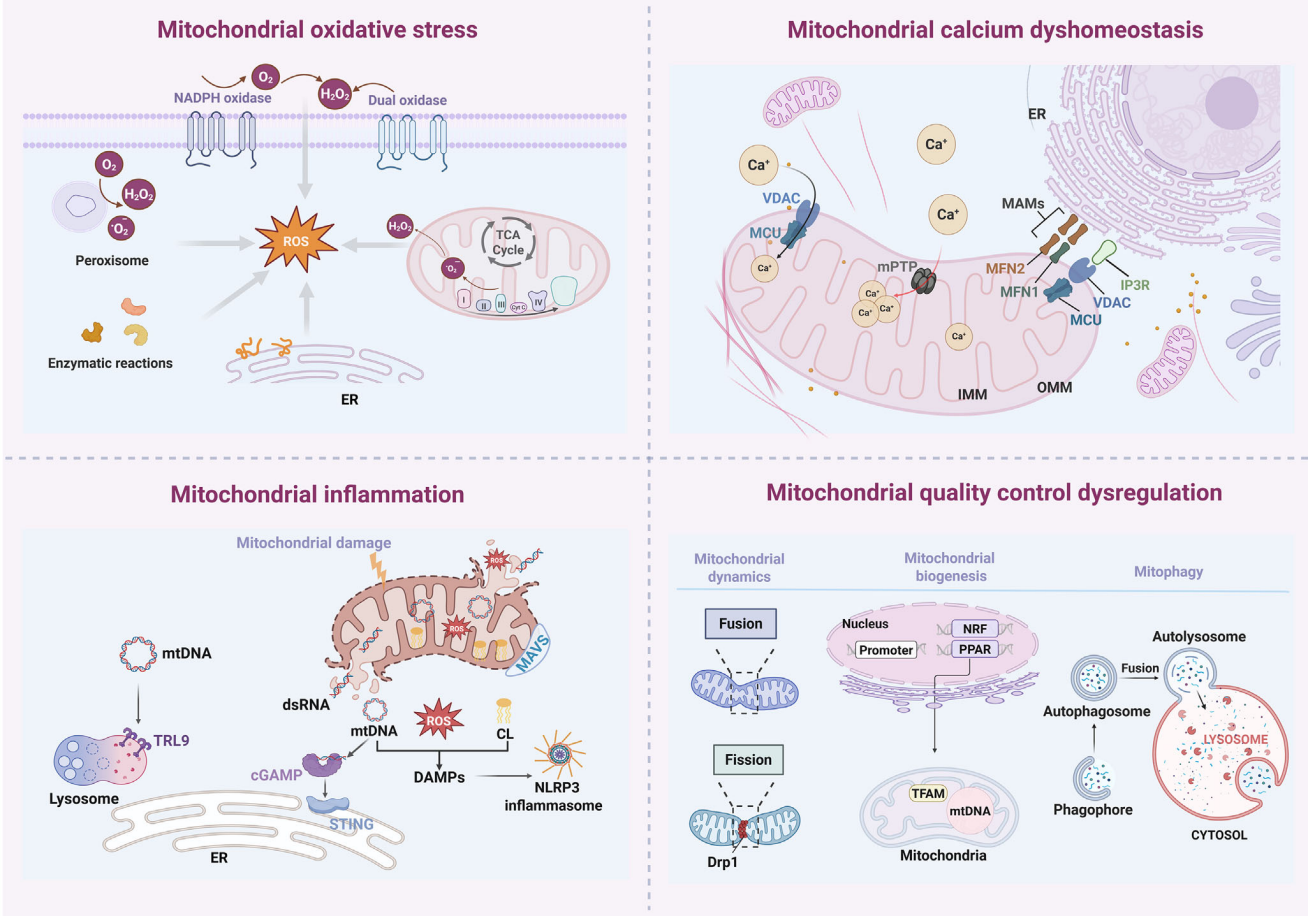
The four core mechanisms of mitochondrial dysfunction, oxidative stress, calcium imbalance, inflammatory response, and quality control imbalance. These four mechanisms are intertwined to form a vicious cycle, which together lead to mitochondrial dysfunction. Created in https://BioRender.com.

Mitochondrial dysfunction is due to a multifaceted, interrelated pathological mechanism (**Figure** **2**). The primary driver of this process is oxidative stress, defined as electron leakage in the ETC, which leads to excessive production of ROS. Once ROS levels exceed the scavenging capacity of antioxidant defense systems (e.g., superoxide dismutase (SOD), catalase (CAT), glutathione peroxidase (GPx, and glutathione), this results in damage to lipids, proteins, and mitochondrial DNA. This process has been shown to induce an aberrant opening of the mitochondrial mPTP, ultimately leading to a collapse in energy metabolism and subsequent cell death.^[^ ^29^, ^30^, ^31^ ^]^ Second, calcium homeostasis imbalance is a critical link. Calcium ions enter the mitochondria through two channels, the inner membrane mitochondrial calcium uniport (MCU) complex and the outer membrane voltage‐dependent anion channel (VDAC) channel. These ions accumulate at the mitochondrial‐associated endoplasmic reticulum (MAM) interface. Calcium overload has been demonstrated to trigger sustained mPTP opening, resulting in mitochondrial swelling, ETC uncoupling, and energy depletion. This phenomenon contributes to a self‐perpetuating cycle, in which oxidative stress exerts a synergistic effect, leading to the induction of cell death.^[^ ^32^ ^]^ These ions subsequently accumulate at the endoplasmic reticulum‐mitochondria contact sites, also known as mitochondria‐associated endoplasmic reticulum membranes (MAMs). Calcium overload in a high‐conductance state has been demonstrated to trigger sustained opening of the mPTP, resulting in mitochondrial swelling, ETC uncoupling, and energy depletion. Moreover, calcium overload has been shown to induce cell death, forming a malicious cycle with oxidative stress. Third, mitochondrial damage leads to the release of damage‐associated molecular patterns (DAMPs, such as mtDNA and ROS), thereby inducing mitochondrial‐related inflammatory responses.^[^ ^33^, ^34^ ^]^ Finally, the impairment of the mitochondrial quality control system (MQC) is pivotal for the perpetuation of dysfunction. The failure of any component of the MQC results in the accumulation of dysfunctional mitochondria, which, in turn, drives disease progression.^[^ ^35^ ^]^

These mitochondrial defects disrupt cellular energy metabolism, lipid homeostasis, and redox balance, thereby triggering systemic metabolic disorders through the “mitochondrial‐metabolic axis” and inter‐organ communication.^[^ ^9^ ^]^ We use the liver as an example. Mitochondrial dysfunction is marked by a diminished capacity for fatty acid oxidation, which is evidenced by decreased carnitine palmitoyltransferase 1 (CPT1) activity, increased uncoupling, elevated ROS levels, and exacerbated inflammatory responses.^[^ ^36^ ^]^ This pathological change is a key driver in the progression of NAFLD/nonalcoholic steatohepatitis (NASH) and insulin resistance, often preceding the emergence of clinical symptoms. Consequently, conducting a thorough investigation into mitochondrial dysfunction and its systemic ramifications is imperative for comprehending the pathological underpinnings of metabolic diseases and devising effective intervention strategies.^[^ ^37^ ^]^

## 3 Current Clinical Treatment Strategies

Mitochondrial dysfunction plays a pivotal role in various metabolic diseases, and treatment strategies for these conditions typically rely on pharmaceuticals and/or surgical interventions, as well as lifestyle modifications.

The development of pharmaceutical interventions is often derived from mitochondrial bioenergetic regulation. Antioxidants (e.g., α‐lipoic acid (α‐LA)and coenzyme Q10) have been shown to restore mitochondrial redox homeostasis, alleviate ROS accumulation caused by electron transport chain leakage, and exert targeted protective effects on diabetic neuropathy and cardiovascular complications.^[^ ^26^, ^38^ ^]^ Insulin sensitizers (e.g., metformin and PPARγ agonists) can directly activate the AMPK/PGC‐1α axis, thereby promoting mitochondrial biogenesis and enhancing the oxidative phosphorylation efficiency in skeletal muscles and liver cells.^[^ ^39^ ^]^ Furthermore, exogenous supplementation of metabolic substrates, such as carnitine, has been demonstrated to correct fatty acid β‐oxidation disorders and reverse mitochondrial lipotoxicity damage in NAFLD.

Surgical interventions are often recommended for certain cases. For patients with moderate to severe obesity and insulin resistance, metabolic surgeries such as Roux‐en‐Y gastric bypass and sleeve gastrectomy have demonstrated mitochondrial repair effects in addition to weight reduction. The underlying mechanisms involve the release of gut hormones glucagon‐like peptide‐1 (GLP‐1), peptide YY (PYY) to promote the conversion of brown adipose tissue and inhibit hepatic lipid neogenesis.^[^ ^40^ ^]^ A comprehensive review of the clinical evidence indicates that the activity of the mitochondrial respiratory chain complexes in liver cells can return to normal ranges after 72 weeks post‐surgery. This finding suggests that surgical interventions can exert a comprehensive regulatory effect on the multi‐organ mitochondrial network.^[^ ^41^ ^]^

Finally, lifestyle changes have become the cornerstone for the treatment of metabolic disorders associated with mitochondrial dysfunction. One of these changes is nutritional uptake. A ketogenic diet with limited caloric content has been shown to enhance myocardial mitochondrial ATP production by increasing ketone body utilization. In addition, polyphenolic compounds, such as resveratrol, have been observed to activate mitophagy through SIRT1 deacetylation, thereby leading to the clearance of dysfunctional mitochondria.^[^ ^42^, ^43^ ^]^ In the context of exercise training, the induction of PGC‐1α expression in skeletal muscle, triggered by physical activity, orchestrates a complex series of events leading to enhanced mitochondrial dynamics, particularly within the fusion process. This, in turn, has been shown to positively impact insulin resistance, a hallmark of the obesity‐related condition.^[^ ^44^ ^]^ In the context of sleep management, the regulation of sleep rhythm and the management of stress have been shown to play a pivotal role in preserving the stability of the mitochondrial genome by reducing cortisol levels.^[^ ^45^, ^46^ ^]^

In light of the organ‐interaction characteristics from the mitochondrial network, the integration of therapeutic modalities encompassing pharmacotherapy, surgical interventions, and lifestyle modifications has emerged as a promising approach, which is capable of amplifying treatment outcomes through a synergistic effect.^[^ ^47^ ^]^ However, current pharmaceutical therapies are challenged by issues including a low delivery efficiency, insufficient targeting, and heterogeneous individual responses; therefore, the development of innovative strategies based on precision medicine is actively explored.^[^ ^48^ ^]^

## 4 Nanomedicine for Targeted Treatment of Mitochondrial Dysfunction

Nanomedicine represents a transformative paradigm in the treatment of metabolic diseases driven by mitochondrial dysfunction, and it offers multi‐dimensional solutions to overcome the limitations of current pharmaceutic therapies.^[^ ^49^ ^]^ Engineered nano‐carriers can protect unstable therapeutic agents from oxidation during delivery, including ROS‐sensitive antioxidants (e.g., α‐lipoic acid and coenzyme Q10).^[^ ^22^, ^50^ ^]^ Additionally, advanced cross‐barrier delivery systems allow precise targeting at the subcellular level.^[^ ^51^ ^]^ In contrast to conventional “static” single‐pathway interventions, modular nanoplatforms employ a synergistic multi‐target therapeutic approach by concurrently delivering mitochondrial repair agents (e.g., superoxide dismutase mimetics), metabolic modulators (e.g., small‐molecule AMPK activators), and genome editing tools to address multifactorial mitochondrial pathologies. It is noteworthy that smart microenvironment‐responsive nanocarriers (e.g., pH‐sensitive liposomes and ROS‐triggered nanogels) have been demonstrated to facilitate spatiotemporal drug release in pathological microenvironments.^[^ ^52^ ^]^ Lesion‐specific accumulation and on‐demand release for synergistic treatment have been demonstrated at an acidic pH, in atherosclerotic plaques, or in ROS‐elevated NAFLD livers.^[^ ^53^ ^]^ The integration of precise delivery, multimodal co‐regulation, and microenvironment‐responsive release by nanomedicine could resolve pharmacokinetic‐pharmacodynamic conflicting issues, such as insufficient drug accumulation and non‐targeted effects.^[^ ^54^ ^]^ Furthermore, nanomedicine establishes a systematic therapeutic paradigm from mitochondrial function restoration to metabolic homeostasis reconstruction.

### 4.1 Classification of Nanomedicines for Mitochondrial Therapy

**Figure 3:**
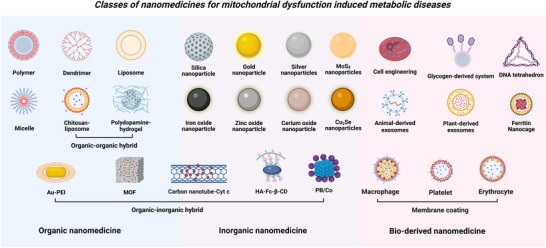
Classification of mitochondrial‐targeted nanomedicine for metabolic diseases. Nanomedicine platforms for overcoming mitochondrial dysfunction in metabolic diseases are divided into three core categories based on their material composition—inorganic, organic, and biomimetic hybrid systems. Created in https://BioRender.com.

Nanomedicine, in a broader sense, employs advanced nanotechnology to prepare medicines within the 1–1000 nm scale range for diagnosis, monitoring, prevention, and treatment of diseases.^[^ ^55^ ^]^ At present, nanomedicine for mitochondrial therapy can be classified into several primary categories based on their material composition (**Figure** **3**), inorganic, organic, hybrid, and bio‐derived systems.

#### 4.1.1 Inorganic Nanomedicines

Inorganic nanomaterials, characterized by their strong chemical stability and diverse programmable functionality, facilitate precise delivery of therapeutic agents. Notable examples of inorganic nanomaterials employed for mitochondrial therapy to treat metabolic diseases include mesoporous silica nanoparticles (MSNs), metallic particles, and metal oxides. Among these nanomaterials, MSNs have emerged as a preeminent carrier.^[^ ^56^ ^]^ For instance, silicon‐based nanomedicines loaded with the mitochondrial uncoupler 2,4‐dinitrophenol methyl ether (DNPME) are structurally designed to leverage the mesoporous channels of MSNs for efficient drug loading and sustained release.^[^ ^57^ ^]^ In an alternative oral formulation design, hollow mesoporous silica (HMS) was utilized as a carrier for ammonium borate. The hollow structure of the material provides a reaction space for H2 release under conditions of acidity in the stomach, forming the core of an oral hydrogen nano‐generator (HMS/A@GE).^[^ ^58^ ^]^

The structural advantage of drug delivery systems based on gold nanoparticles lies in their surface's ease of functionalization.^[^ ^59^ ^]^ The gold nanoparticle‐miRNA complex system (Au@16‐ph‐16/miR‐21 mimic) designed by Lhamyani et al. features a key structural characteristic. The binding of the Gemini surfactant (16‐ph‐16) to the gold nanoparticle surface forms a lipophilic interface, thereby enhancing the system's ability to interact with the lipid bilayer of adipose tissue.^[^ ^60^ ^]^ Surprisingly, Silva et al. demonstrated that gold nanoparticles scavenged ROS and inhibited oxidative stress, suggesting that gold nanoparticles may have the potential for treating metabolic diseases.^[^ ^61^ ^]^ Additionally, silver nanoparticles (AgNPs) synthesized using plant extracts exhibited a remarkable antidiabetic property. The synthesis of AgNPs or their complexes with plant extracts (ExAgNP) frequently utilizes plant extracts as reducing agents and stabilizers, effectively mitigating diabetes‐induced mitochondrial oxidative stress and brain dysfunction.^[^ ^62^ ^]^

In addition, inorganic core‐shell structured nanoparticles can display integrated diagnostic and therapeutic functions. The iron oxide‐cerium oxide core‐shell nanoparticles (Fe_3_O_4_@CeO_2_, IO@CO) were developed by Wu et al. These particles consisted of superparamagnetic iron oxide (Fe_3_O_4_) with a core diameter of ≈10 nm as a magnetic resonance imaging (MRI) contrast agent, and an outer layer of cerium oxide (CeO_2_) with a shell thickness of ≈2 nm displayed a regenerative antioxidant property. The surface of the IO@CO nanoparticles was modified with polyacrylic acid (PAA) to enhance their stability and biocompatibility. This core‐shell structure ingeniously integrated both diagnostic (Fe_3_O_4_ to provide excellent T_2_‐weighted MRI contrast) and therapeutic (CeO_2_ to efficiently scavenge ROS such as H_2_O_2_ and superoxide anions through its reversible Ce^3^⁺/Ce⁴⁺ redox cycle on the surface) functions.^[^ ^63^ ^]^ The nanomedicine may hold great promise for treating ROS‐related inflammatory diseases, including atherosclerosis and rheumatoid arthritis.

It is noteworthy that bioaccumulation and toxicity risks of inorganic nanomedicines represent significant obstacles to their clinical translation. One effective strategy to address these issues is to functionalize inorganic nanomedicines with organic compounds to improve their degradability and regulate their metabolic behavior in vivo.^[^ ^64^ ^]^ For instance, the silicon‐based nanocarriers previously described could be modified through the introduction of thioether bonds during their construction process. When organosilicon compounds containing thioether bonds (TKOS) enter the target tissue (e.g., the T2D liver) with a high‐concentration ROS microenvironment, the thioether bonds are specifically cleaved by ROS, leading to the disintegration of the entire silica shell. This strategy has been demonstrated to prevent the development of chronic inflammation or fibrosis, which is typically associated with prolonged retention of inorganic nanomedicines.^[^ ^57^ ^]^

#### 4.1.2 Organic Nanomedicines

The prevalence of biocompatible nanostructures lies in organic materials. Organic nanomedicines have been extensively utilized in a variety of biomedical applications, including drug delivery.^[^ ^65^ ^]^ At present, organic nanomedicines for the treatment of mitochondrial‐related metabolic diseases are derived from a range of organic nanocarriers, including liposomes, polymeric particles, polymeric micelles, and dendrimers.

Lipid nanoparticles (LNPs) are defined as a closed vesicular system that exhibit core‐shell morphology for encapsulating and delivering therapeutic cargo, and their particle size ranges from nanometers to micrometers. LNPs have become a prevalent nanocarrier in mitochondrial metabolic therapy due to their biocompatibility, biodegradability, and manufacturing scalability.^[^ ^66^, ^67^ ^]^ Furthermore, potent visceral obesity‐targeted therapy in vivo has been achieved by modifying the lipid composition and physicochemical properties of cationic liposomes. For instance, the incorporation of cholesterol into third‐generation polyamide‐amine (PAMAM) dendritic polymers (P‐G3) to form lipid NPs was found to enhance their lipophilicity, thereby improving the visceral fat targeting efficiency. In comparison to unmodified P‐G3, the off‐target rate in other organs, such as the liver and kidneys, was reduced by 30%.^[^ ^68^ ^]^ To address the need for liver regeneration, Rizvi et al. administered nucleoside‐modified mRNA‐LNPs to deliver hepatocyte growth factor (HGF) and epidermal growth factor (EGF) via intravenous injection, and the mRNA‐LNPs displayed a high liver‐targeting efficiency. A hepatocyte transfection rate of 95% was achieved with sustained protein expression for 3 days.^[^ ^69^ ^]^ To tune NASH inflammation regulation, Zhou et al. developed mannosyl‐modified siRNA‐LNPs to target macrophages, resulting in a significant decrease in the M1/M2 phenotype ratio from 0.88 to 0.32.^[^ ^70^ ^]^

Polymeric nanomedicines are prepared using natural or synthetic polymers. Polymers can be designed to meet specific requirements by tuning their physical and chemical properties. Covalent or non‐covalent combinations are employed for co‐loading drugs to achieve better delivery efficacy. Poly(lactic acid‐co‐glycolic acid) (PLGA), an FDA‐approved biodegradable polymer, has been widely used in the construction of smart delivery nanoparticles.^[^ ^71^ ^]^ For instance, Katsuki et al. employed an emulsified solvent diffusion method to prepare a PLGA nanomedicine loaded with nifedipine to stabilize atherosclerotic plaques, and the nanomedicine achieved sustained drug release at the lesion site for up to seven days.^[^ ^72^ ^]^ Hou et al. incorporated phase‐change materials, perfluorohexane (PFH) and dextran sulfate (DS), into a PLGA‐PEG‐PLGA nanomedicine to form an FPD@CD composite system. This nanomedicine with an ultrasound‐responsive property was employed for the treatment of unstable atherosclerotic plaques.^[^ ^73^ ^]^

Polymer micelles are typically formed by the self‐assembly of amphiphilic block copolymers, featuring a hydrophobic core and a hydrophilic shell. Mu et al. constructed a ROS‐responsive polymer micelle with a core composed of polyethylene glycol‐polytyrosine‐ethyl oxalyl (PEG‐Pyr‐EO) and coated the micelle with a hyaluronic acid (HA) shell on its surface via electrostatic adsorption. The PEG‐Pyr‐EO@HA micelle leveraged the CD44 targeting property of HA on the shell to significantly enhance the enrichment efficiency of the micelle in atherosclerotic plaques. The accumulated micelle amount was 1.8 times higher than that in the unmodified micelle.^[^ ^74^ ^]^

Dendrimers constitute a class of highly branched, 3D, spherical, single‐molecular nanomaterials.^[^ ^75^ ^]^ They are obtained through precise algebraic expansion of a “core” small molecule outward in a repetitive branching structure. Dendrimers have a nanoscale dimension, a highly branched molecular structure, physical cavities, and abundant surface functional groups. Among dendrimers, PAMAM dendrimers, with their layered branching structure and high‐density surface amino groups, have been shown to achieve glucose capture and drug delivery.^[^ ^9^ ^]^ Siewiera et al. assessed the effects of PAMAM dendrimers on the mitochondrial respiratory function and associated toxicity in rat hearts. The findings suggested that third‐generation (G3) PAMAM dendrimers exhibited biological toxicity.^[^ ^76^ ^]^ A comparison of G3 and G4 PAMAM dendrimers revealed that the G3 exhibited a greater propensity for metabolic regulation, while the G4 displayed a balance between mitochondrial protective effects and biological toxicity.^[^ ^77^ ^]^ Concurrently, Huang et al. developed a butterfly‐shaped dendrimeric macromolecule (C&D@Z) for multi‐drug co‐delivery after arginine modification.^[^ ^78^ ^]^ Additionally, Xian et al. demonstrated a molecular recognition‐based smart assembly strategy where pyridine‐diboronic acid (DiPBA)‐modified insulin and diol‐modified G6 PAMAM form nanocomposites through electrostatic interactions and dynamic covalent bonds.^[^ ^79^ ^]^

Organic‐organic hybrid nanomedicines combine different organic materials to integrate their respective advantages and expand functionality. For example, chitosan‐lipid nanoparticles (PP‐CLNPs) developed by Darwish et al. exhibit a typical core‐shell structure. The lipid matrix, composed of stearic acid, serves as the core for encapsulating hydrophobic drugs. The outer layer consists of a coating formed by chitosan polysaccharide chains through ionic crosslinking (e.g., sodium TPP). The design of this structure is intended to leverage the high drug loading capacity of the lipid core and the mucosal adhesion properties of the chitosan shell synergistically.^[^ ^80^ ^]^ Similarly, Chen et al. developed SS/MPDA@RES hydrogels. In the hydrogels, the polyphenol‐based mesoporous polydopamine (MPDA) organic core was used to efficiently load resveratrol, while the outer layer of methyl acrylate‐modified silk protein/silk microfiber network provided injectable, photopolymerizable, and bioadhesive properties. The two organic components were integrated at the molecular level, thereby overcoming the limitations associated with low drug solubility and facile metabolism while concurrently imparting ROS scavenging and immune regulation functions. This integrated strategy offers a novel approach for diabetic wound repair.^[^ ^81^, ^82^ ^]^

#### 4.1.3 Organic–Inorganic Hybrid Nanomedicines

Organic‐inorganic hybrid nanomedicines leverage the properties inherent in both organic and inorganic materials, and these hybrid nanomedicines have the potential to offer synergistic therapeutic treatment to metabolic disorders.^[^ ^83^ ^]^ Au‐PEI/shSiglec‐1/PEI‐ASA nanoparticles (ASPA NPs) developed by Zhou et al. were an example of a hybrid nanomedicine. Short hairpin RNA targeting the Siglec‐1 gene was loaded onto gold nanoparticles through electrostatic adsorption. The gold nanoparticles were coated with polyethyleneimine (PEI‐ASA) that was covalently modified with aspirin (ASA). This precise hybrid structure endowed the nanocarrier with multiple functions. The gold core provided a stable platform for gene delivery and exhibited antioxidant activity; the PEI‐ASA polymer layer possessed a charge inversion property, enabling pH‐responsive cleavage and drug (ASA) release in an acidic environment of endosomes, and the layer also protected the gene payload.^[^ ^84^ ^]^ This organic/inorganic synergistic hybridization collectively remodeled the atherosclerotic immune microenvironment.

Additionally, Zhang et al. developed zeolite imidazolate framework‐8 (ZIF‐8) nanoparticles loaded with losartan potassium (LP) (LP@ZIF‐8). ZIF‐8 is a metal‐organic framework (MOF) formed by self‐assembly of inorganic zinc ions (Zn^2+^) and organic ligands 2‐methylimidazole (2‐MI) through coordination bonds, and it is a typical organic‐inorganic hybrid material. The ZIF‐8 framework functioned as an efficient drug carrier for LP. This nanoscale platform achieved a dual therapeutic action of “simultaneous lipid clearance and anti‐inflammatory responses”.^[^ ^85^ ^]^

The composite of carbon nanotubes and biomacromolecules represents another type of hybrid form. Shukla et al. utilized carboxylated single‐walled carbon nanotubes (cSWCNTs) as an inorganic scaffold to anchor positively charged cytochrome c (Cyt‐C) onto the nanotube surface through electrostatic adsorption. The cSWCNT scaffold provided a protective microenvironment for Cyt‐C, shielding it from rapid degradation and enzymatic clearance in vivo, thereby significantly extending its functional half‐life. This protection is attributed to the combination of electrostatic interaction and the steric barrier presented by the nanotube. In addition, the ferrous heme active center of Cyt‐C was coupled to the conductive network of carbon nanotubes, thus this nanomedicine displayed a dual catalytic activity and acted as a peroxidase and catalase‐like enzyme.^[^ ^86^ ^]^

The implementation of multi‐level cooperative hybrid systems, characterized by their intricate structural designs, has been observed to result in the manifestation of morphological transformations during delivery. A representative example is a ROS‐responsive size‐reversible nano‐assembly (HA‐Fc/NP) developed by He et al. This nano‐assembly had a three‐layer hybrid structure. The core consisted of disc‐shaped recombinant high‐density lipoprotein (rHDL) loaded with simvastatin (ST). β‐Cyclodextrin (β‐CD) was anchored to the surface of rHDL, providing critical binding sites for subsequent assembly. The outermost layer consisted of a copolymer formed by grafting amide‐bonded ferrocene (Fc) onto HA (HA‐Fc). Fc, functioning as a hydrophobic metal‐organic group, served as a guest molecule that bound to β‐CD on the core through host‐guest interaction, thereby enabling multivalent assembly of multiple NPs into larger nanoparticles. This multi‐level hybrid structure was engineered to achieve “on‐demand conversion” for intelligent delivery. The innovation of this structure is evident in its ROS‐responsive disassembly capability, ROS oxidation of Fc disrupts host‐guest interactions, causing the large‐sized assembly to dissociate into small‐sized rHDL core particles capable of deep tissue penetration. Experimental findings have demonstrated that the administration of large‐sized HA‐Fc/NP^3sT^ nanoparticles can effectively prolong the circulation time of the nanoparticles in the bloodstream.^[^ ^87^ ^]^

#### 4.1.4 Bio‐Derived Nanomedicines

In addition to organic and inorganic materials, bio‐derived nanomedicines have gained increasing interest. Bio‐derived nanomedicines are prepared by directly incorporating pharmaceutical drugs into natural biological components or biomimicking components, including but not limited to cells, cell membranes, extracellular vesicles, and biomolecules. This approach imparts distinctive biological activity and targetability to bio‐derived nanomedicines.

Among bio‐derived nanomedicines, biomimetic nanomedicines stand out due to their unique preparation approach. The preparation of bionic nanomedicines generally entails the fusion of synthetic nanocores with natural cell membranes. The preservation of the source cell's membrane proteins and functions constitutes their fundamental structural characteristic. This approach has several advantages, including immune evasion, homogenous targeting, and prolonged circulation of nanomedicines.^[^ ^88^ ^]^ To treat atherosclerosis, Chen et al. developed platelet membrane‐coated MSNs (PMSNs) and kept platelet membrane surfaces intact, thereby preserving key membrane proteins, including CD47, on the PMSNs. This resulted in effective evasion of phagocytosis of PMSNs by mononuclear macrophages and an extended circulation half‐life of 48 h in comparison to unmodified MSNs.^[^ ^89^ ^]^ Furthermore, Wang et al. developed macrophage membrane‐coated biomimetic nanoparticles (MM/RAPNPs) by functionalizing PLGA nanoparticles with macrophage membranes. These nanoparticles exhibited a high degree of specificity because of the presence of integrin α4β1 on the membrane. They bind to vascular cell adhesion molecule‐1 (VCAM‐1) on the surface of endothelium through integrin α4β1, resulting in a targeting efficiency three times higher than unmodified particles.^[^ ^90^ ^]^ The bionic hybrid nanodrug developed by Fei et al. further complicates the structure, featuring an inorganic silicon skeleton containing diselenium bonds as its core, it is coated with a macrophage membrane modified by CD44 aptamers, forming a multilayer structure comprising an “inorganic core‐organic shell‐biological membrane.”^[^ ^91^ ^]^ In addition to utilizing homologous biological membranes, engineering strategies for modifications of biological membranes to achieve key functions of specific membranes, such as prolonged circulation and targeting, have been extensively explored. Red blood cell membrane‐mimetic nanoparticles (RBC‐NPs) displayed optimized distribution to the liver and spleen due to their natural deformability and long circulation characteristics.^[^ ^92^ ^]^ For instance, Miao et al. developed red blood cell membrane‐mimicking nanocarriers, which led to a substantial reduction in lung accumulation and efficient enrichment in liver lesions by modulating the particle shape and the elasticity parameters through red blood cell membranes.^[^ ^93^ ^]^

In addition to the use of cell membranes to encapsulate pharmaceutic drugs to prepare biomimetic nanodrugs, cell engineering is another important strategy for preparing bio‐derived nanodrugs. For example, the red blood cell‐micelle complex (SV MC@RBCs) developed by Shen et al. is notable for its unique structure. It is loaded with simvastatin, and it features positively charged polymeric micelles (SV MCs) that adsorb onto the negatively charged surface of natural red blood cells via electrostatic interactions. This results in the formation of a “cell carrier‐nanodrug” composite structure, rather than encapsulating the cell membrane within nanoparticles. This construction method retained the integrity of the RBC membrane and its natural proteins (e.g., CD47), thereby achieving effective evasion of the immune system clearance and significantly prolonging the circulation time of the complex.^[^ ^94^ ^]^

Exosomes, bio‐derived natural extracellular vesicles, have garnered increasing attention as an efficient drug delivery carrier to carry therapeutic agents, including proteins and nucleic acids.^[^ ^95^, ^96^ ^]^ In a seminal study, Lu et al. demonstrated that mesenchymal stem cell exosomes (MSC‐exos) restored the mitochondrial function in endothelial cells under a high‐glucose condition. In a diabetic periodontal bone defect model, hydrogels loaded with MSCs‐exos (Exo@Gel) promoted vascularized bone regeneration.^[^ ^97^ ^]^ It is noteworthy that, in addition to traditional mammalian cell‐derived exosomes, plant‐derived exosome‐like nanovesicles have become more attractive in recent years due to inherent richness produced by plant bioactive cells, abundant plant sources, a low cost, and a high safety profile; therefore, they could be a great candidate for preparing nanomedicine for the treatment of metabolic diseases. For instance, Wang et al. developed reconstituted turmeric nanovesicles (Rec‐tNVs) to overcome limitations of naturally derived turmeric nanovesicles, such as a low curcumin loading capacity and low purity.^[^ ^98^ ^]^ The studies reported so far suggest that exosome‐based nanotherapy holds significant potential for the treatment of metabolic diseases by regulating the mitochondrial function and intercellular signaling pathways.

### 4.2 Organ and Mitochondria Targeting by Nanomedicines

**Figure 4:**
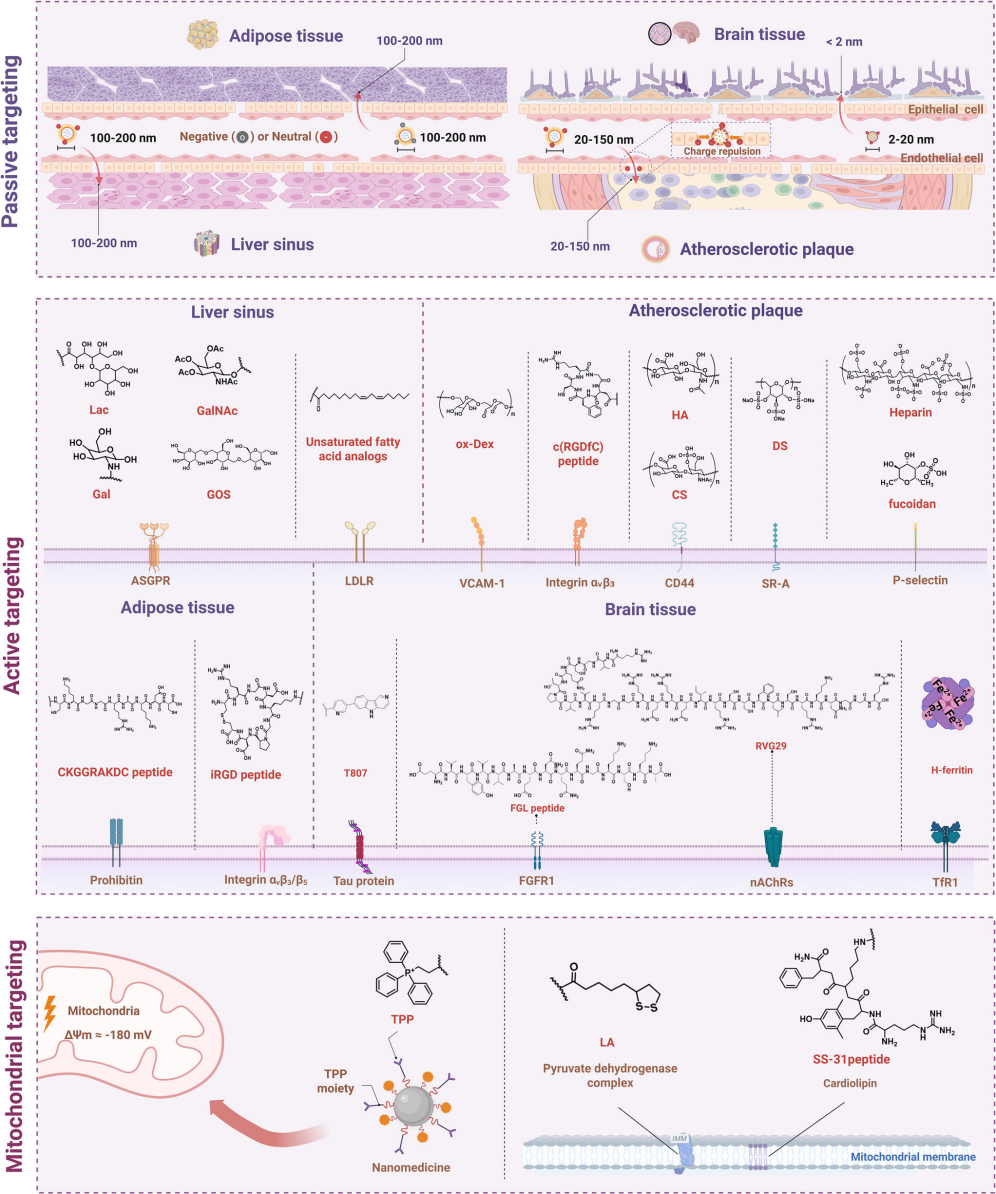
Targeted strategies for nanomedicine from organs to subcellular organelles (mitochondria) in metabolic diseases. Passive targeting of nanomedicines in the liver (100–200 nm, negative or neutral zeta‐potential), atherosclerotic plaques (20–150 nm, negative or neutral zeta‐potential), and adipose tissue (100‐200 nm, negative or neutral zeta‐potential). Active targeting of the liver, atherosclerotic plaques, adipose tissue, and mitochondria is realized through ligand‐receptor interaction. Liver, ASGPR receptor and its ligands (Gal, Lac, and GalNAc);^[^ ^102^, ^106^, ^107^ ^]^ LDLR receptor and its ligands (unsaturated fatty acid analogues). Atherosclerotic plaque, VCAM‐1 receptor and its ligand (ox‐Dex);^[^ ^108^ ^]^ integrin α4β1 and its ligand (RGDfc peptide); CD44 receptor and its ligands (HA and CS);^[^ ^109^ ^]^ SR‐A receptor and its ligand (DS);^[^ ^73^ ^]^ P‐selectin and its ligands (heparin and fucoidan).^[^ ^81^, ^110^ ^]^ Adipose tissue, Prohibitin receptor and its ligand (CKGGRAKDC peptide);^[^ ^111^ ^]^ integrin αvβ3/β5 and its ligand (iRGD peptide).^[^ ^112^ ^]^ Brain, Tau protein and its ligand T807; nAChR and its ligand RVG29; FGFR1 and its ligand FGL peptide; TfR1 and its ligand H‐ferritin.^[^ ^113^, ^114^, ^115^, ^116^ ^]^ For mitochondrial targeting, nanomedicine exploit the high negative membrane potential of ≈−180 mV within the mitochondrial inner membrane. Positively charged TPP groups can passively accumulate in mitochondria driven by this membrane potential. Additionally, active targeting can be achieved through ligands specifically binding to mitochondrial membrane proteins, such as lipoic acid (LA) or SS‐31 peptide, enabling the nanodrug to penetrate the double membrane and accumulate efficiently within the matrix.^[^ ^117^, ^118^ ^]^ ASGPR, Asialoglycoprotein receptor, CD44, CD44 Receptor, CS, Chondroitin sulfate, DS, Dermatan sulfate, FGFR1, Fibroblast growth factor receptor 1, FGL, FGFR1‐binding peptide, GAL, Galactose, GalNAc, N‐Acetylgalactosamin, GOS, Galactose‐oligosaccharide, HA, Hyaluronic acid/Hyaluronan, LA, Lipoic acid, LAC, Lactobionic acid, LDLR, Low‐density lipoprotein receptor, ox‐Dex, Oxidized dextran, RVG29, 29‐mer rabies virus glycoprotein peptide, SR‐A, Scavenger receptor class A, TfR1, Transferrin receptor 1, T807, Tau‐targeting ligand (AV‐1451), VCAM‐1, Vascular cell adhesion molecule‐1. Created in https://BioRender.com.

The therapeutic efficacy of mitochondrially targeted nanomedicines could be significantly improved by implementing effective organ‐ and subcellular organelle‐targeting strategies. There are two fundamental paradigms of drug delivery nanosystems, passive targeting and active targeting. Passive targeting relies on anatomical structural features, such as an interstitial space size and tissue permeability differences, to achieve passive drug accumulation. In contrast, active targeting to achieve precise delivery is primarily through ligand‐receptor molecular recognition.^[^ ^99^, ^100^ ^]^ In this section, a systematic analysis of three pivotal target organs was performed, including the liver, atherosclerotic plaques, and adipose tissue, due to their pivotal roles in metabolic regulation and their distinctive anatomical and molecular characteristics (**Figure** **4**)^[^ ^101^, ^102^, ^103^, ^104^, ^105^ ^]^.

Furthermore, conventional pharmaceuticals are often ineffective in targeting specific subcellular compartments, resulting in non‐specific intracellular distribution and significant toxicity. Mitochondria, a semi‐autonomous organelle with a double‐membrane structure (inner membrane potential ΔΨm ≈ −180 mV), possess unique physicochemical properties, such as a negative membrane potential and specific membrane proteins, which could provide a molecular basis for targeted design of nanomedicine. Nanomedicine may integrate passive targeting driven by the mitochondrial membrane potential (e.g., electrophoretic enrichment of cationic ligands, such as TPP) with active targeting mediated by ligands. For example, the recognition of mitochondrial membrane proteins by lipoic acid (LA) has been demonstrated to enable the nanomedicine containing therapeutic molecules with a coating layer of LA to overcome dual barriers of plasma and mitochondrial membranes and achieve efficient accumulation in the mitochondrial matrix.^[^ ^51^ ^]^ In this chapter, we will systematically elaborate on the mitochondria‐targeting strategies.

#### 4.2.1 Liver‐Targeting Nanomedicines

The liver, the core organ responsible for regulating metabolism, serves as a pivotal target for the treatment of metabolic diseases. Its anatomical structure is unique, featuring a large intercellular space in hepatic sinusoids and a high density of Kupffer cells. Additionally, it possesses cell‐specific receptors, including asialoglycoprotein receptor (ASGPR) and low‐density lipoprotein receptor (LDLR), which provide unique multiple targets for the design of liver‐specific nanomedicines.^[^ ^105^ ^]^

The utilization of passive targeting in liver‐targeting nanomedicines for mitochondrial therapy can enhance the accumulation of therapeutic drugs in nanomedicines driven by the anatomical structure of the liver. A distinctive endothelium‐interstitial space (100–200 nm) and the highly permeable basement membrane of the hepatic sinusoids offer a natural advantage for passive targeting of nanomedicines, facilitating their passive accumulation in the liver through size‐matching.^[^ ^119^ ^]^ For example, the baicalin nanoliposome (BA‐NL), developed by Liu's team, features a particle size of 81.4 nm, which is designed to match the dimensions of hepatic sinusoids for enhanced passive capture.^[^ ^105^ ^]^ Rao et al. developed passive targeting‐based phosphatidylcholine‐cholesterol liposomes (EPC, CHOL) and phosphatidylcholine‐phosphatidylglycerol liposomes (EPC, EPG) with a particle size of ≈100–200 nm and without additional targeting ligand modifications. These liposomes achieved passive enrichment through the natural phagocytic activity of hepatic inflammatory myeloid cells. In a mouse model of NASH induced by a MCD diet, EPC, CHOL liposomes encapsulating curcumin were passively enriched in the liver and internalized by 87 ± 4% of F4/80+ dendritic cells (DCs) in the liver. Conversely, EPC, EPG liposomes encapsulating calcitriol were observed to bind to CD14+ myeloid cells in human liver perfusion cells in a passive manner, resulting in a reduction of over 50% in the proportion of LPS‐induced TNF‐α^+^ and IL‐6^+^ cells.^[^ ^120^ ^]^ These studies have revealed that by optimizing the particle size, surface charge, and lipid composition of nanomedicines, the enhanced permeability and retention (EPR) effect and phagocytosis‐dependent passive targeting efficacy can be maximized. It is noteworthy that a significant number of liver‐targeting nanomedicines, including cerium dioxide nanoparticles (nCeO_2_), zinc oxide nanoparticles, and chitosan nanoparticles, employ this passive retention mechanism to accumulate in liver tissue. The factors associated with this mechanism include their particle size of less than 100 nm) and a negative surface charge, and these factors of nanomedicines facilitate their penetration of the endothelium and prolong their persistence in the liver.^[^ ^101^, ^121^, ^122^ ^]^ These studies have demonstrated that liver‐targeting anti‐inflammatory treatment could be achieved through the EPR effect by manipulating the particle size and surface charge of nanomedicines without ligand‐receptor interactions.

However, receptor‐mediated active targeting enables precise recognition of liver cells. Hepatocytes exhibit a high expression level of multiple specific receptors, among which the ASGPR and LDLR are two promising targets.^[^ ^99^ ^]^ Galactooligosaccharides (GOS) can bind to the ASGPR through terminal galactosyl groups, and nanomedicines with GOS moieties enter hepatocytes via receptor‐mediated endocytosis, significantly improving the nanomedicine uptake efficiency by hepatocyte.^[^ ^123^ ^]^ Teng et al. covalently coupled D‐(+)‐galactose (D‐Gal), which can specifically recognize the ASGPR receptor on the surface of liver cells, to the carboxyl groups of TEMPO‐oxidized starch, ultimately synthesizing the Gal‐OS polymer. This polymer was similar to the lysozyme micelles (LysM) in liver targeting.^[^ ^102^ ^]^ Furthermore, lactobionic acid (Lac) with a galactose residue has been identified as a target ligand to specifically recognize ASGPR overexpressed in hepatocytes.^[^ ^124^ ^]^ Consequently, surface modification of nanomedicines with lactobionic acid can facilitate active targeting and augment drug accumulation in hepatocytes. Li et al. constructed redox‐responsive nanoparticles (Gly‐LA‐Lac/ResNPs) with glycogen (Gly) as the backbone and modified them with both α‐LA and Lac for liver‐targeting delivery of resveratrol (Res).^[^ ^107^ ^]^ Active targeting through specific recognition of ASGPR, highly expressed on hepatocytes could be an efficient approach to deliver nanomedicines into hepatocytes through lactate acid surface modification.

Furthermore, active targeting of LDLR on the hepatocyte surface facilitates drug delivery and plays a direct role in lipid metabolism regulation. When liver cell LDLR expression is activated, modifying the surface of nanomedicines with LDLR‐specific ligands (e.g., unsaturated fatty acid analogues) can enhance their uptake by hepatocytes and promote metabolic clearance of low‐density lipoprotein cholesterol (LDL‐C). This approach was demonstrated to reduce foam cell formation and induce apoptosis of pro‐inflammatory macrophages, thereby synergistically lowering the total cholesterol level in serum.^[^ ^110^, ^125^ ^]^

Despite notable advancements in liver‐targeting strategies, several challenges remain. In advanced NASH, the liver sinusoids can undergo a reduction in size down to 50 nm, thus size‐adaptive nanomedicines should be formulated to accommodate the changes in the sinusoid size.^[^ ^119^ ^]^ In addition, ASGPR is expressed in organs such as the intestines and kidneys, which may lead to extrahepatic drug accumulation.^[^ ^123^ ^]^ Furthermore, the sinusoidal gaps in mouse livers (average 150 nm) are larger than those in humans (100 nm), and the targeting efficacy of nanomedicines for humans may be overestimated from animal experiment data. A critical examination has revealed notable differences between animal experimentation and clinical translation.^[^ ^126^ ^]^ The development of size‐adaptive nanomedicines (e.g., using shape‐changing nanocarriers), the optimization of ligand‐receptor affinity (e.g., synergistic recognition of dual targets), and the combination of passive‐active targeting with multimodal delivery strategies could overcome delivery barriers in the liver, achieve precise regulation of liver cell subpopulations (e.g., parenchymal/non‐parenchymal cells), and advance clinical translation and personalized treatment of metabolic liver diseases.

#### 4.2.2 Atherosclerotic Plaques‐Targeting Nanomedicines

Precise targeting of atherosclerotic plaques is a major challenge in the treatment of metabolic diseases. Pathological characteristics of plaques, including endothelial cell damage, inflammatory infiltration, neovascularization, and oxidative stress, offer a multitude of targets for the development of nanomedicines.^[^ ^104^ ^]^

Nanomedicines can be designed to target mitochondria associated with atherosclerotic plaques in a passive targeting manner. The disrupted microenvironment within atherosclerotic plaques (e.g., inflammation‐mediated increased vascular permeability and loose extracellular matrix) can be harnessed for passive targeting of nanomedicines via the EPR effect.^[^ ^104^ ^]^ By optimizing the size (20–150 nm), surface charge (neutral or negatively charged), and hydrophobicity of nanomedicines, their accumulation within plaques can be significantly enhanced. For example, amorphous selenium quantum dots (A‐SeQDs) (a particle size of ≈50 nm; a negatively charged surface) penetrated the damaged endothelial barrier via passive diffusion, preferentially accumulated in the plaque region, promoted endothelial cell migration, and repair.^[^ ^103^ ^]^ However, reliance on the EPR effect may be challenged by the heterogeneity of neovasculature within plaques, which results in uneven drug distribution and complicates precise intracellular drug delivery and functional performance of nanomedicines.^[^ ^104^ ^]^ In order to address this issue, an intelligent nanomedicine system was developed to integrate microenvironment response characteristics into the optimization of the intracellular delivery process of nanomedicines. Zhou et al. constructed pH‐responsive charge‐reversible nanoscale systems (ASPANPs) with an initial negative/neutral surface charge to facilitate EPR effect enrichment. Upon entering acidic lysosomes within plaque macrophages, the PEI‐ASA component within their structure undergoes protonation, transforming the surface charge to positive and promoting lysosomal escape.^[^ ^84^ ^]^ The dynamic regulation concept of “lesional localized microenvironment response” in this design provides new insights into overcoming temporal and spatial limitations of the EPR effect.^[^ ^104^ ^]^

In addition, ligand‐modified nanosystems can achieve precise delivery to lesions by targeting specific markers within plaques (such as VCAM‐1, CD44, and SR‐A).^[^ ^73^, ^127^, ^128^ ^]^ Ox‐LDL deposited in the endothelium has been shown to induce the activation of receptors that are highly expressed in endothelial cells and inflammatory cells, such as CD44 and VCAM‐1. These receptors serve as targets for the enrichment of nanomedicines. Nasr et al. developed a HA‐conjugated atorvastatin nanodrug (HA‐ATV‐NP), and achieved precise targeting of macrophages within plaques through specific binding of HA on the nanodrug to the CD44 receptor on macrophages.^[^ ^127^ ^]^ Xu et al. reported a cascade‐targeted nanoplatform (PA/ASePSD) whose surface‐coated oxidized dextran (ox‐Dex) can sequentially recognize endothelial VCAM‐1 and macrophage CD44, achieving dual‐stage targeting.^[^ ^128^ ^]^ In addition to VCAM‐1 and CD44, the macrophage scavenger receptor A (SR‐A) has been identified as a specific marker within plaques. For instance, the ultrasound‐responsive nanomedicine (FPD@CDNPs) developed by Hou et al. targets the SR‐A receptor on macrophages via surface electrostatic adsorption of DS. This process significantly enhanced the active targeting efficiency of the nanodrug at the lesion site.^[^ ^73^ ^]^

It is important to acknowledge that dynamic changes in the receptor expression level within the plaque microenvironment may influence the targeting efficiency. For instance, CD44 expression may be downregulated in macrophages during the progression of inflammation, leading to ineffectiveness in targeting via single‐ligand strategies. To address this challenge, Wang et al. proposed biomimetic delivery nanoparticles (MM/RAP NPs) by functionalizing rapamycin (RAP) nanoparticles with macrophage membranes. In this biomimetic design, specific binding between integrin α4β1 on the macrophage membrane surface and VCAM‐1 on the plaque endothelium was harnessed to realize active targeting in inflammatory regions.^[^ ^92^ ^]^

However, current targeted delivery to the atherosclerotic plaque environment is confronted with significant challenges, including 1) the plaque heterogeneity, which may affect the enrichment of nanomedicine in plaques due to differences in the target expression level in specific regions at different stages, and 2) cross‐reactivity between active targeting ligands and receptors on non‐target tissues, which may raise safety concerns.^[^ ^104^, ^129^ ^]^ In response to these challenges, pH/ROS‐responsive nanoscale systems modified with personalized combined multiple ligands based on single‐cell sequencing could be developed to address the differences in the target expression level at different stages of plaque development and reduce cross‐reactivity with non‐targeted tissues.

#### 4.2.3 Fat Tissue‐Targeting Nanomedicines

Adipose tissue, notably white and brown adipose tissue, functions as a primary organ for the storage of energy and the regulation of metabolism. Its dysfunction (e.g., adipocyte hypertrophy, inflammatory infiltration, and browning disorders) is closely related to obesity, insulin resistance, and metabolic syndrome.^[^ ^130^ ^]^ The anatomical structure of adipose tissue is unique, and it is characterized by a loose extracellular matrix and a high vascular density. Additionally, adipose tissue contains cell‐specific receptors, including β3‐adrenergic receptor and PPARγ, which provide multiple binding sites for targeted design.^[^ ^100^ ^]^

The intercellular space in adipose tissue is relatively large (≈100–200 nm), and nanomedicines with an appropriate size of 20‐150 nm can be passively enriched.^[^ ^131^ ^]^ For example, locally injected PLGA polymer nanodrugs typically have particle sizes controlled between 100 and 200 nm to facilitate diffusion and retention within white adipose tissue (WAT).^[^ ^132^ ^]^

A high expression level of specific receptors (such as PPARγ and prohibitin) in adipocytes can be leveraged as a molecular basis for active targeting.^[^ ^111^, ^112^ ^]^ For instance, the targeting peptide CKGGRAKDC has been shown to bind specifically to the inhibin receptor. The targeted nanomedicine (PTNP) constructed based on this peptide and enables precise targeting of WAT.^[^ ^111^ ^]^ Modification of a nanomedicine with targeted peptides of iRGD and P3 peptides that can be bound to integrin αvβ3/β5 receptors and prohibitin receptors, respectively, helped achieve dual targeting of vascular endothelial cells in WAT. Notably, this strategy enabled the delivery of rosiglitazone (a PPARγ activator) and prostaglandin E2 analogues to promote fat tissue browning and angiogenesis, thereby forming a positive feedback loop to enhance the targeting efficiency.^[^ ^112^ ^]^ Furthermore, the positive charge property of chitosan and its mucosal adhesion can enhance electrostatic interaction between chitosan and the adipocyte membrane. Chitosan‐derived nanocarriers, through surface modification with a target ligand such as Lac, can specifically recognize the prohibitin receptor on the surface of vascular endothelial cells in WAT. This specific recognition could result in an improved targeted delivery efficiency.^[^ ^133^ ^]^

Fat tissue targeting faces multiple challenges, specifically, 1) fat tissue heterogeneity leads to differences in the receptor expression level in adipocytes under different metabolic states (obese/normal), such as the abundance of prohibitin and β3‐adrenergic receptors, therefore, single‐ligand targeting strategies are prone to failure due to downregulation of the receptor for that single ligand;^[^ ^133^ ^]^ 2) although a loose matrix allows passive diffusion of nanomedicines, they may result in a short retention time and uneven distribution of nanomedicines;^[^ ^130^ ^]^ and 3) the size of the intercellular gap in the adipose tissue vascular endothelium undergoes dynamic changes between 100 and 200 nm at different pathological stages (e.g., vascular remodeling in the late stages of obesity), which may influence the size‐dependent passive targeting efficiency.^[^ ^130^ ^]^ Consequently, the development of dual/multiple‐targeted ligand systems (e.g., those capable of simultaneous targeting of inhibin and β3‐AR) and size‐dynamically tunable nanocarriers emerges as a promising design direction to address this heterogeneity.^[^ ^100^, ^131^ ^]^

#### 4.2.4 Brain‐Targeting Nanomedicines

In contrast to the “fenestrated permeability” of hepatic sinusoids, the blood‐brain barrier (BBB) is sealed by tight junctions with a virtually negligible paracellular pathway. It exhibits strict selectivity for trans‐BBB transport, passive diffusion is largely confined to small lipophilic molecules (≈<1–2 nm), whereas hydrophilic molecules scarcely traverse via paracellular routes.^[^ ^134^ ^]^ Even so, when nanoparticles fall within a physicochemical window of “ultrasmall size (2–20 nm) + mild surface charge”, they can cross the BBB without exogenous ligands via adsorptive‐mediated transcytosis (AMT) and membrane interactions. With respect to surface charge, a mildly positive ζ‐potential (≈ +5 to +15 mV) markedly strengthens electrostatic adsorption to the negatively charged glycocalyx, thereby enhancing AMT. However, overly cationic surfaces intensify protein corona formation, complement activation, and cytotoxicity/hemolysis, and accelerate clearance by the reticuloendothelial/mononuclear phagocyte system. By contrast, near‐neutral (±5 mV) or mildly negative (−5 to −15 mV) charges afford more stable circulation with reduced protein adsorption, albeit with more conservative BBB flux—hence best used synergistically with ultrasmall sizing.^[^ ^135^ ^]^ For example, u‐MoS_2_ quantum dots (≈4 nm hydrated diameter) bearing a slightly negative, near‐neutral ζ‐potential can enter brain tissue without receptor‐targeting ligands such as Angiopep‐2 or anti‐TfR antibodies, subsequently restoring membrane fluidity and inhibiting Aβ aggregation to improve mitochondria‐related oxidative stress and energy homeostasis.^[^ ^136^ ^]^

To surmount the BBB more efficiently, receptor‐mediated transcytosis (RMT) has become a mainstream strategy for active brain delivery of nanomedicines.^[^ ^114^ ^]^ This approach decorates nanocarriers with ligands that specifically recognize receptors on brain endothelial cells, thereby initiating receptor endocytosis, vesicular transcytosis, and basolateral exocytosis—ultimately increasing drug exposure in the brain parenchyma and improving selectivity for particular regions or cell types.^[^ ^137^, ^138^ ^]^ A key route is the TfR1‐H‐ferritin axis, human heavy‐chain ferritin (H‐ferritin) nanocages are recognized by transferrin receptor‐1 (TfR1) and transported across the BBB. In PD models, carotenoid‐loaded H‐ferritin nanodots exploited this pathway to achieve central nervous system (CNS) accumulation and to improve dopaminergic neuronal phenotypes—showcasing the unique advantages of this “protein cage‐receptor” strategy for loading hydrophobic drugs and enabling genetic engineering.^[^ ^116^ ^]^

Recently, a variety of ligand‐modified biomimetic nanosystems have been engineered to boost brain‐targeting efficiency. Gao et al. developed red‐blood‐cell‐membrane–coated nanoparticles co‐modified with T807 and TPP (T807/TPP‐RBC‐NPs), T807 functions as a BBB‐penetrating ligand enabling rapid trans‐BBB passage and neuron binding, while TPP mediates mitochondrial targeting for curcumin delivery, significantly alleviating Alzheimer's‐related symptoms.^[^ ^113^ ^]^ Similarly, Han et al. designed dual‐ligand biomimetic nanocarriers (RVG/TPP NPs@RBCm) bearing RVG29 for efficient brain targeting and TPP for mitochondrial localization; systemic delivery of resveratrol with this platform improved neuronal function and cognition.^[^ ^114^ ^]^ Beyond endothelial‐receptor targeting, leveraging receptors enriched on specific neuronal subtypes can further enable lesion‐focused and cell‐specific delivery. Qian et al. reported a cholinergic neuron‐targeting system, FGL‐NP(Cit)/HNSS, using a citraconylated PEG‐PTMC polymer to electrostatically load the cationic hybrid peptide HNSS (SS‐31 fused with S14G‐Humanin). Single‐ligand decoration with FGFR1‐binding peptide (FGL) achieved multistage targeting, because fibroblast growth factor receptor 1 (FGFR1) is highly expressed on BBB endothelial cells and on cholinergic neurons in AD‐affected regions (e.g., hippocampus, cortex), FGL conferred selective accumulation in FGFR1‐positive cholinergic neurons—rather than other neurons or glia—after BBB traversal, yielding up to 4.8‐fold higher brain accumulation and 87% neuronal specificity.^[^ ^115^ ^]^

Despite the considerable advancement in the field of brain‐targeting nanomedicines that receptor‐mediated cross‐endocytosis strategies have brought, there is still a need for designs that are far more complex than simple “ligand‐receptor” docking in order to translate this potential into clinical reality. The successful design of a given project requires a delicate balance to be achieved among multiple critical parameters. First, it is imperative that the ligand‐receptor affinity is moderate. Excessively high affinity may lead to “endothelial retention/circulatory endocytosis”, thereby reducing transcellular flux, while excessively low affinity may fail to effectively trigger transport.^[^ ^139^, ^140^ ^]^ Second, it is essential that ligand density and valence balance transmembrane efficiency with avoiding receptor saturation.^[^ ^114^, ^140^ ^]^ Third, it is crucial to enhance endosomal escape and drug release efficiency to reduce lysosomal degradation. It is imperative to minimise peripheral tissue non‐specific uptake and manage protein cap and off‐target risks, given that relevant receptors are also expressed in peripheral tissues.^[^ ^141^, ^142^, ^143^ ^]^ For instance, the FGL‐NP(Cit)/HNSS system undergoes hydrolysis at a pH of ≈5–6 due to citrullination of the side chain. This triggers a “near‐neutral to cationic” charge reversal that disrupts endosomal membranes and enables effective peptide release.^[^ ^115^ ^]^ The tandem design of “RMT brain penetration + acid‐sensitized escape” significantly enhances effective exposure and therapeutic windows.

#### 4.2.5 Mitochondria‐Targeting Nanomedicines

Conventional drug delivery systems have demonstrated the capacity to achieve targeting at the organ or cellular level; however, they can not precisely regulate the pathological microenvironment of subcellular organelles, such as mitochondria. This limitation stems from an insufficient delivery efficiency of these drug delivery systems during crossing multiple biological barriers and breaching the complex microenvironmental regulation at the subcellular level.^[^ ^144^ ^]^ Consequently, the development of mitochondria‐targeting nanomedicines that can overcome the multi‐level delivery barriers at the organelle level has emerged as a pivotal strategy for the treatment of metabolic diseases.^[^ ^145^ ^]^ Presently, a number of mitochondrial‐specific ligands are available for modifying nanoparticles to achieve their mitochondrial targeting. The ligands encompass TPP, tetramethylrhodamine‐5‐isothiocyanate, mitochondrial‐targeting peptides, dequalinium, and natural products (such as hypericin and glycyrrhetinic).^[^ ^146^ ^]^ However, the mitochondrial‐targeting strategies that have been used for the treatment of mitochondrial metabolic diseases are relatively few.

Among mitochondrial targeting strategies, manipulation of the physicochemical properties of nanomedicines for targeting mitochondria is primarily driven by the mitochondrial membrane potential (ΔΨm).^[^ ^145^ ^]^ The inner mitochondrial membrane maintains a transmembrane potential of ≈−180 mV due to the electron transport chain, with a negative charge on the inner surface and a positive charge on the outer surface. This characteristic can be leveraged by cationic molecules or nanomedicines to efficiently accumulate in the mitochondrial matrix through electrophoresis. TPP is a prototypical mitochondria‐targeting ligand. Its cationic property allows its penetration into the mitochondrial bilayer membrane and efficient accumulation in the mitochondrial matrix under the driving force of ΔΨm.^[^ ^147^, ^148^ ^]^ Covalent modification of the surface of nanocarriers with TPP has been shown to significantly enhance the targeting ability of nanocarriers toward mitochondria. The success of the TPP strategy is evident not only in the core carrier itself but also in the evolution of mitochondrial‐targeted nanomedicines toward multifunctionality and intelligence through integration with other functional modules. First, TPP modification has been shown to significantly enhance the targeting efficiency of traditional nanocarriers. A hybrid nanodrug platform, TPP‐modified PLGA‐b‐PEG polymers (PLGA‐b‐PEG‐TPP) blended with non‐targeted polymers (e.g., PLGA‐b‐PEG‐OH or PLGA‐COOH), was developed by Marrache et al. It was shown that the TPP‐modified nanomedicine with a surface charge of ≈+34 mV exhibited a significantly higher mitochondrial uptake level by HeLa cells compared to non‐targeted particles, and the Pearson correlation coefficient between surface charge and cellular uptake level was 0.53 and 0.03 for the TPP‐modified nanomedicine and non‐targeted particles, respectively. Additionally, the proton sponge effect of TPP facilitated the escape of the nanomedicine from endosomes/lysosomes, further enhancing the targeting efficiency of the nanomedicine toward mitochondria. This mechanism effectively prevented non‐specific degradation of the drug in the nanomedicine within cells, thereby enhancing its subsequent therapeutic outcome.^[^ ^149^ ^]^ Additionally, Li et al. developed a natural antioxidant nanomedicine, T4O@TPP/PEG−PLGA, to target mitochondria by covalently modifying TPP onto the surface of a biodegradable PLGA‐b‐PEG block copolymer. The nanomedicine had a diameter of ≈178 nm and a surface charge of +20 mV. This study validated the TPP modification strategy for mitochondrial targeting with a significant increase in the mitochondrial uptake efficiency in human vascular smooth muscle cells (VSMCs) and marked inhibition of vascular calcificatio.^[^ ^150^ ^]^ Furthermore, TPP has been extensively utilized in constructing delivery systems that traverse complex biological barriers. For instance, in the treatment of CNS disorders, Ren et al. anchored TPP to DSPE‐PEG‐modified MoS_2_ quantum dots (TPP‐MoS_2_ QDs). This design enabled the nanoparticles to traverse BBB and effectively evade lysosomes, resulting in their accumulation within neuronal mitochondria. In a cell model of AD, the mitochondrial membrane potential in the TPP‐MoS_2_ QDs‐treated group exhibited a recovery of 85% of normal levels, which is significantly higher than the unmodified group (≈60%).^[^ ^151^ ^]^ Li et al. constructed multifunctional tetrahedral DNA nanostructures (TDFNs) that were modified with TPP and cholesterol (to enhance BBB penetration). In vivo distribution data demonstrated that TDFNs accumulated 3.5 times more in the brain than unmodified DNA tetrahedra and accurately delivered therapeutic antisense oligonucleotides (ASOs) to mitochondria, reducing target mRNA expression levels by ≈70% in an AD model.^[^ ^152^ ^]^ Furthermore, the integration of TPP strategies with biomimetic techniques has led to the development of a new generation of smart nanomedicines. Zheng et al. reported a biomimetic nanosystem (CSCCT NPs) featuring a core of resveratrol‐loaded Cu_2_Se nanoparticles, coated with a macrophage membrane modified with DSPE‐PEG‐TPP. The nanoparticle accumulation in the inflammatory regions of the PD model mouse brains was enhanced by 4.2‐fold by this dual‐targeting design (cell membrane‐mediated inflammatory chemotaxis + TPP‐mediated mitochondrial chemotaxis). PGC‐1α expression was significantly increased (≈1.8‐fold), and it is a key indicator of mitochondrial biogenesis. Consequently, motor dysfunction was improved.^[^ ^153^ ^]^ In a similar vein, Xia et al. developed “lycopene nanodots” by encapsulating lycopene within recombinant human H‐ferritin nanocages and coupling them with TPP. In a mouse model of PD, the system demonstrated not only the delivery of the drug to the mitochondria of dopaminergic neurons but also the restoration of dopamine levels in the striatum to 90% of the control group.^[^ ^154^ ^]^

In addition to passive targeting driven by physicochemical properties of mitochondria, there is a growing interest in ligand‐mediated active targeting strategies to optimize mitochondrial drug delivery. LA functions as a mitochondrial localization signal molecule, and it can bind to mitochondrial membrane proteins, such as the thioesterase complex. This binding process has been demonstrated to promote the penetration of nanocarriers with LA through the mitochondrial bilayer membrane, thereby resulting in their enrichment within the mitochondrial matrix.^[^ ^155^ ^]^ Shen et al. developed an LA‐modified targeted nanocarrier (PA NPs) through simultaneous enzyme‐catalyzed polymerization and esterification reactions. Horseradish peroxidase‐catalyzed hydroxylation of platycodin D resulted in a polymer backbone, and LA was covalently grafted onto the surface of the platycodin D nanomedicine. Mitochondrial colocalization experiments in cells demonstrated that the targeting efficiency of PA NPs was ≈1.62 times higher than that of free platycodin D, significantly reversing the pathological state in diabetic mice.^[^ ^117^ ^]^ Integration of high‐efficiency enzyme‐catalyzed polymerization with great‐specificity LA targeting could significantly improve the efficacy of active targeting in complex disease models.

Furthermore, Xu et al. developed a novel mitochondrion‐targeting antioxidant peptide, SS‐31. This peptide was shown to penetrate cell membranes and accumulate in the mitochondrial matrix via electrostatic interactions. After reaching mitochondria via active targeting, it directly inhibited ROS production, normalized the mitochondrial membrane potential (ΔΨm), and promoted ATP synthesis.^[^ ^128^ ^]^ Qian et al. further designed a smart delivery system based on the targeting potential of SS‐31. This system involved loading the hybrid peptide HNSS, which contains the SS‐31 sequence, onto acid‐sensitive PEG‐PTMC(Cit) nanoparticles. The surface of the nanoparticles was modified with the FGFR1 ligand FGL peptide to target cholinergic neurons. Analysis of mitochondrial colocalization revealed that the smart nanoparticle‐released HNSS, guided by the SS‐31 fragment, exhibited a ≈2.46‐fold higher enrichment efficiency in neuronal mitochondria than the control group. This enabled precise intervention of neuronal mitochondria.^[^ ^115^ ^]^ These studies not only validate the potential of peptide molecules as mitochondrial targeting ligands but also provide a novel therapeutic perspective for restoring mitochondrial function.

A review of the extant literature reveals that the effectiveness of mitochondrial targeting methods varies significantly. With regard to overall performance, lipophilic cations driven by mitochondrial membrane potential (ΔΨm)—represented by TPP demonstrate the highest mitochondrial enrichment when optimized for both size and surface charge, Marrache et al. demonstrated that a typical formulation of targeted nanoparticles (diameter ≈79 nm; **ζ** ≈ +27.4 mV) exhibited high fluorescence overlap with mitochondria, whereas control particles of identical size but with negative charge (**ζ** ≈ −26.5 mV) showed almost no overlap. ICP‐MS quantification revealed optimal mitochondrial uptake in the 80–100 nm size range, with positive charge showing significant correlation (r≈0.5) with uptake. This finding indicates an empirical window of “80‐100 nm, +25–35 mV” for TPP systems.^[^ ^149^ ^]^ However, TPP analogues also exhibit dose‐dependent limitations, Substituted TPP derivatives have been observed to induce measurable membrane depolarization at concentrations ranging from 125 to 250 nM, suggesting the possibility of disruption to the mitochondrial membrane potential and the respiratory chain at elevated exposure levels. Mitigation necessitates the implementation of slow‐release strategies, including carrier density dilution, cleavable linkers, and pulsed administration.^[^ ^156^ ^]^ Peptides and signal peptides (e.g., SS‐31) have been shown to exhibit higher biorecognition and functional restoration potential through protein interactions or cardiolipin binding. However, these formulations often encounter challenges related to sequence degradability, formulation stability, and controlled release, which can be addressed by incorporating protective shells or environment‐triggered designs.^[^ ^157^, ^158^, ^159^ ^]^ It has been demonstrated that small molecules and natural product ligands, including rhodamine and diquat, possess a variety of chemical spaces. This property facilitates the development of probes and combination therapy. However, concerns regarding immunogenicity, synthetic complexity, and off‐target/accumulation issues limit their application in the context of systemic long‐term administration.^[^ ^160^, ^161^ ^]^

### 4.3 Stimuli‐Responsive Strategies to Enhance Therapeutic Efficacy for Mitochondrial‐Related Metabolic Diseases

Metabolic diseases frequently display dynamic and heterogeneous microenvironmental characteristics, including, but not limited to, an acidic pH level at inflammatory sites (e.g., pH 5.0–6.5 at atherosclerotic plaque), a high ROS level in diabetic wounds (a ROS concentration of 3–5 times of that in normal tissues), and a mitochondrial redox potential gradient (ΔΨm) of ≈−180 mV.^[^ ^162^, ^163^ ^]^ These pathological features have been harnessed to develop smart nanomedicines to realize precise regulation of the microenvironmental characteristics.^[^ ^164^ ^]^ However, conventional nanocarriers exhibit inadequate capacity in dynamic responsiveness and rapid adaptation to spatiotemporal variations in the microenvironment.^[^ ^165^, ^166^ ^]^ For example, during the treatment of atherosclerosis, nanomedicine that relies solely on passive targeting via the EPR effect is often unevenly distributed within plaques and the distribution of the nanomedicine is not dynamically changed to map the local ROS concentration, thus drug release is out of synchronism with pathological dynamic changes;^[^ ^132^ ^]^ Transient burst of mitochondrial oxidative stress, such as a sudden increase in the ROS level during myocardial ischemia‐reperfusion, requires nanomedicine at a response rate within a millisecond level.^[^ ^167^ ^]^ In addition, mitochondria, a semi‐autonomous organelle, exhibit a highly dynamic membrane potential (ΔΨm) and a rapidly changing redox state. Traditional non‐responsive nanomedicines are prone to drug leakage during circulation or suffer from insufficient retention due to membrane potential fluctuations.^[^ ^168^ ^]^

**Figure 5:**
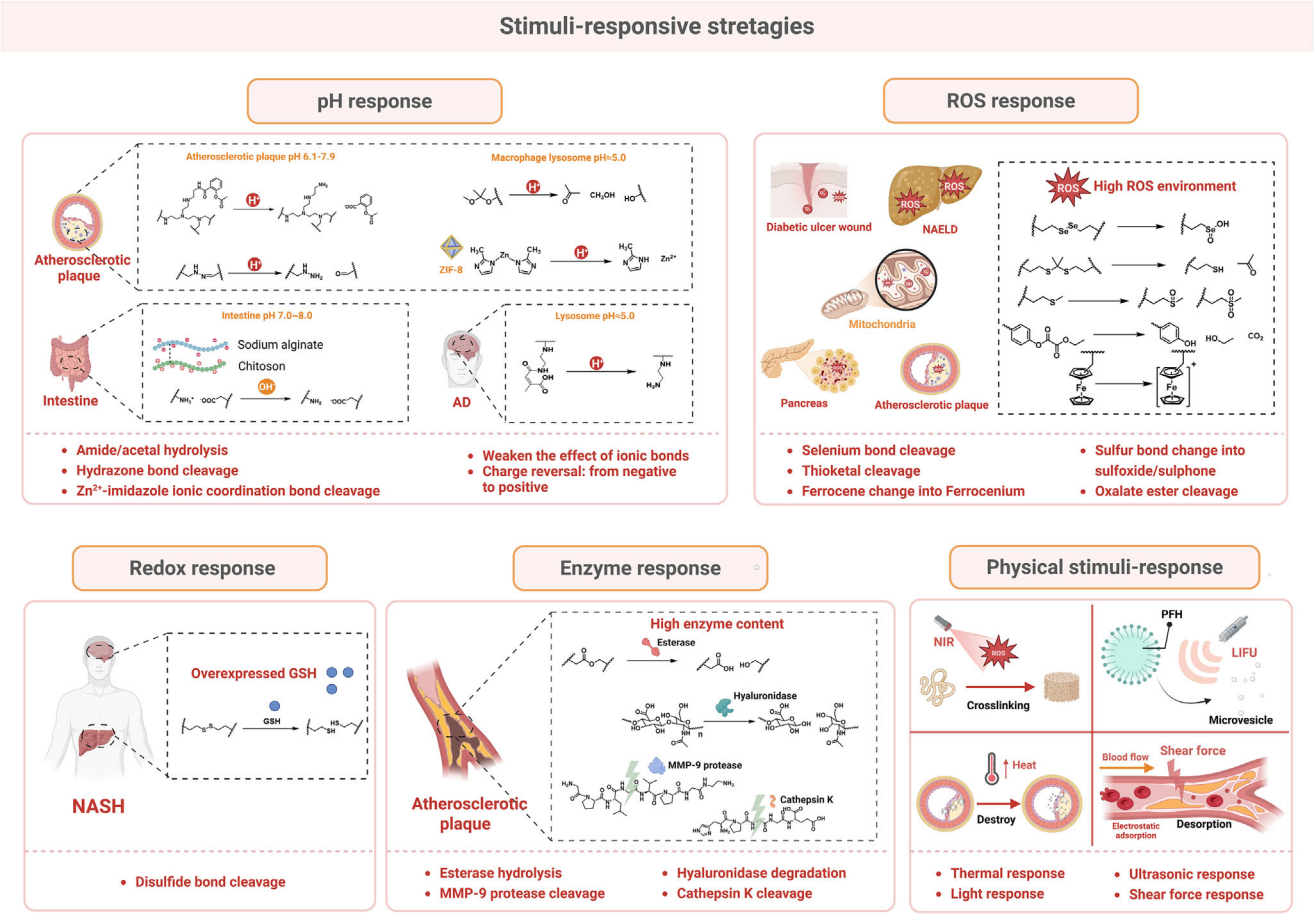
Stimuli‐responsive strategies of nanomedicine. This schematic illustrates multiple stimuli‐responsive mechanisms for smart nanomedicines to achieve spatiotemporal and dose‐controlled drug release at the site of diseases, significantly enhancing the efficacy of mitochondrially targeted therapies of metabolic diseases. Key strategies include pH‐responsiveness through targeted release at acidic atherosclerotic plaques (e.g., hydrolysis of amide/aldehyde condensation reactions, cleavage of imine bonds, or disruption of Zn^2^⁺‐imidazole coordination),^[^ ^84^, ^109^, ^128^, ^169^ ^]^ in the alkaline intestinal fluid (e.g., weakening of ionic bonds)^[^ ^170^ ^]^ and in AD, acid‐catalyzed hydrolysis of the β‐carboxamide bond occurs in the acidic environment of lysosomes, leading to charge inversion and drug release;^[^ ^115^ ^]^ ROS‐responsiveness triggered by a high ROS level in organs and mitochondria (e.g., Se‐Se bond cleavage, thioether bond cleavage, oxidation of aryl borate esters to phenol/boric acid, oxidation of ironene to ironene lactone, or oxidation of sulfides to sulfones/sulfoxides),^[^ ^74^, ^87^, ^94^, ^171^, ^172^ ^]^ glutathione (GSH)‐responsiveness by utilizing a high GSH concentration in the liver or brain tissues (e.g., disulfide bond cleavage)^[^ ^107^, ^173^ ^]^ to trigger drug release, and enzyme‐responsiveness through activation by specific enzymes enriched in organs (e.g., esterase hydrolysis, MMP‐9 protease cleavage, hyaluronidase degradation, or cathepsin K cleavage).^[^ ^89^, ^172^, ^174^ ^]^ Physical stimuli‐responses are triggered by external factors to achieve precise control (e.g., thermal response, light response, ultrasound response, or shear force response).^[^ ^73^, ^94^, ^118^, ^175^ ^]^ GSH, Glutathione, LIFU, Low‐Intensity Focused Ultrasound, MMP‐9 protease, Matrix Metalloproteinase‐9, NIR, Near‐Infrared Light, PFH, Perfluorohexane. Created in https://BioRender.com.

Two novel strategies have been proposed in response to the aforementioned issues. The first strategy is the development of a microenvironment‐triggered drug release system based on a molecular logic gate mechanism, which is initiated by pathological signals. The other strategy is the advancement of a precise mitochondrial targeting technology based upon environment‐responsive delivery carriers^[^ ^176^ ^]^ (**Figure** **5**). The mitochondrial microenvironmental characteristics, including a pH gradient and a high ROS level, serve as a trigger for disassembly and subsequent reassembly of nanocarrier structures. This process enables spatiotemporal‐specific accumulation and release of therapeutic molecules from nanocarriers at the subcellular level. Consequently, the two integrated strategies enhance the therapeutic action of nanomedicines by precisely regulating mitochondrial dysfunction.^[^ ^168^, ^177^, ^178^ ^]^

The pH gradient response strategy is built on the principle of “acid‐base gate control kinetics”. Through strategic manipulation of chemical bonds, pathological microenvironmental changes (e.g., acidic pH or oxidative stress) can be employed as a “molecular switch” that facilitates the release of therapeutic components from nanomedicines.^[^ ^177^, ^179^, ^180^ ^]^ For example, Zhao et al. developed mitochondria‐targeting nanoparticles (mito‐NP) by innovatively introducing pH‐responsive Meo‐PEG‐b‐PDPA polymers. Leveraging the pH‐sensitive protonation of tertiary amines in its structure, the nanoparticle selectively released cyclic RNASCAR in an acidic endosomal environment (pH 5.0–6.5) to achieve mitochondrial delivery of cyclic RNASCAR to inhibit excessive mtROS production.^[^ ^181^ ^]^ Furthermore, the hydrazone bond in a nanomedicine can undergo hydrolysis in an acidic environment, thereby facilitating controlled drug release from the nanomedicine. Cheraga et al. designed a pH‐responsive HA nanodrug (HRRAP NPs) by covalently coupling antioxidant all‐trans retinoic acid (ATR) to the HA backbone via an acid‐sensitive hydrazone bond and encapsulating an anti‐inflammatory drug, RAP, within the nanodrug. This nanodrug displayed a distinct response within an acidic microenvironment (pH 5.0–6.5) of atherosclerotic plaques. Under these acidic conditions, the hydrazone bond in the nanodrug was cleaved, leading to the disassembly of the nanocarrier. This process facilitated the concomitant release of ATR and RAP.^[^ ^109^ ^]^ Verma et al. developed a pH‐responsive vitamin B12‐functionalized layered calcium phosphate nanoparticle (VitB12‐Chi‐CPNPs) based on chitosan (Chi). The efficiency in oral delivery of insulin was enhanced through vitamin B12 conjugation and a layered structure design. In an acidic environment of the stomach, the amino groups of VitB12‐Chi underwent protonation to form a compact polyelectrolyte layer that prevented insulin release. In a neutral to slightly alkaline environment of the intestine, the deprotonation of chitosan enhanced interlayer electrostatic repulsion, thereby triggering sustained drug release. This innovative approach addressed the challenges of enzymatic degradation, low permeability, and poor targeting associated with oral insulin delivery.^[^ ^182^ ^]^ Furthermore, mitochondrial dysfunction is closely linked to oxidative stress in neurodegenerative diseases such as AD. Qian et al. developed a cholinergic neuron–targeted nanosystem (FGL‐NP(Cit)/HNSS) and utilized a citraconylated PEG‐PTMC polymer to impart pH‐responsive behavior. Within the acidic lysosomal environment (pH ≈ 5.0), the nanoparticles undergo a charge reversal from negative to positive, thereby promoting lysosomal escape and accelerating the intracellular release of the HNSS peptide.^[^ ^115^ ^]^

Compared with pH‐responsive nanomedicines, ROS‐triggered strategies are formulated on the dynamic equilibrium mechanism of redox homeostasis.^[^ ^183^ ^]^ The phenolic hydroxyl groups in mesoporous dopamine nanoparticles (MPDANPs) are a natural chemical structure formed during self‐polymerization of dopamine. These phenolic hydroxyl groups can effectively scavenge excess ROS. Deng et al. loaded a positively charged mitochondrial‐targeting peptide, SS31, onto the negatively charged surface of MPDANPs via electrostatic interactions and hydrogen bonds. MPDANPs were observed to undergo polymer oxidation in the presence of excess ROS, triggering the release of the surface‐adsorbed peptide SS31. In the context of treating diabetic wounds, this release mechanism helped enhancing synergistic effects of MPDANPs and SS31, including the regulation of macrophage polarization, the promotion of cell proliferation and migration, and the enhancement of angiogenesis.^[^ ^118^ ^]^

Furthermore, it is noted that ROS‐responsive nanomedicines based upon inorganic nanomaterials have distinctive advantages in electron transfer regulation. Selenium nanoparticles (SENDs) were observed to release selenium ions within an elevated ROS microenvironment of pancreatic β cells in type 2 diabetes. This release was facilitated by the cleavage of C‐Se covalent bonds, and the cleavage process was ROS‐responsive. The released selenium ions were identified as an essential component for intracellular synthesis of glutathione peroxidase 1 (GPX1), a pivotal antioxidant enzyme. The catalytically active center of GPX1 contains selenocysteine (Sec), and the synthesis of Sec is highly dependent on the supply of selenium ions.^[^ ^184^ ^]^ Therefore, SENDs enhanced clearance of harmful ROS such as H_2_O_2_ and released bioavailable selenium ions to promote the biosynthesis of GPX1 and restore the endogenous GPX1 activity. After SENDs treatment, the mRNA expression of GPX1 in β cells increased to 180% of a healthy level, and the enzyme activity was significantly recovered from 66.5 U L^−1^ in a diabetic model to 174.6 U L^−1^ in a healthy level.^[^ ^171^ ^]^ In a similar manner, Zhang et al. employed the ROS‐responsive degradation property of thioether bonds to fabricate hollow nanocapsules with a biodegradable silica matrix containing TKOS. Ultra‐small platinum nanoparticles were uniformly anchored on the inner walls of nanocapsules, and a mitochondrial uncoupling agent, DNPME, was encapsulated in their cavities. In the presence of an elevated ROS level, such as those observed in diabetic livers, thioether bonds underwent rupture, leading to the degradation of nanocapsules and subsequent release of DNPME and ultra‐small platinum nanoparticles. These platinum nanoparticles functioned as catalysts to facilitate the decomposition of H_2_O_2_ into oxygen and water, thereby relieving ROS stress. DNPME functioned as a mitochondrial uncoupling agent by increasing the permeability of the inner mitochondrial membrane for protons, reducing the proton gradient (ΔΨm), decreasing electron leakage, inhibiting ROS generation in the ETC, and simultaneously enhancing fatty acid oxidation.^[^ ^57^ ^]^ Conversely, single‐walled carbon nanotubes (SWCNTs) conjugated with cytochrome c (CytC@cSWCNT) exhibited efficient ROS‐responsive catalytic activity, attributable to their distinctive nanostructure design. Oxidized single‐walled carbon nanotubes (cSWCNTs) had a carboxyl group (‐COOH)‐abundant surface, which promoted stable conjugation with the positively charged regions of the CytC surface through electrostatic interactions. A high specific surface area of cSWCNT provided abundant catalytic sites for CytC, and its π‐π stacking effect also optimized the electron transfer pathways, therefore, CytC@cSWCNT exhibited an outstanding peroxidase‐mimicking activity.^[^ ^86^ ^]^

Furthermore, a high concentration of GSH, ≈2–10 mM, can be employed as a natural reducing molecule, particularly in liver cells (a concentration of 5–10 mM), followed by the kidneys, red blood cells, and mitochondria (containing 10‐15% of total cellular GSH).^[^ ^185^ ^]^ GSH at a high concentration can specifically recognize and cleave disulfide bonds.^[^ ^186^ ^]^ In contrast, the serum GSH concentration in the extracellular space is extremely low, ≈2–20 µM, and disulfide bonds in nanomedicines can remain intact during their systemic circulation.^[^ ^187^ ^]^ In their seminal study, Yi et al. pioneered the field by performing amination modification on Pluronic P85 block copolymers to yield derivatives that contained terminal primary amino groups (‐NH_2_), such as NH_2_‐PEO‐PPO‐PEO‐NH_2_. Subsequently, bifunctional crosslinking agents containing disulfide bonds (─S─S─) were utilized, including N‐Succinimidyl 3‐(2‐pyridyldithio)propionate (SPDP) and its analogues. The reaction of the amino groups of the modified Pluronic P85 at one end of the disulfide bond and the primary amino groups of the leptin (Lep) protein molecule that are primarily located on the side chains of lysine (Lys) residues or the N‐terminal region of the protein) at the other end of the disulfide bond resulted in the construction of a leptin‐Pluronic P85 conjugate (Lep(ss)‐P85) bridged by disulfide bonds. The Pluronic‐modified leptin nanomedicine utilized the controlled release property of disulfide bonds to prolong the blood circulation half‐life of leptin.^[^ ^173^ ^]^

Thermodynamically responsive nanomedicines have also demonstrated their unique therapeutic potential.^[^ ^188^ ^]^ For instance, thermosensitive hydrogels (PDLLA‐PEG‐PDLLA) incorporating Prussian blue nanoparticles (PBNPs) can rapidly gel at body temperature (37 °C) within 30 s, cover diabetic foot ulcers, and continuously release PBNPs to clear ROS and restore mitochondrial function.^[^ ^175^ ^]^ It is noteworthy to mention that light‐responsive nanostructures have the capacity to achieve precise metabolic regulation through temporally and spatially controllable exogenous physical stimuli. Chen et al. developed silk‐based nanocomposite hydrogels (SS/MPDA@RES) through chemical modification of silk fibroin (SF) with glycidyl methacrylate (GMA) to introduce methacrylate groups (─C═C─) into the silk protein. These groups subsequently underwent free radical polymerization reactions, catalyzed by a photoinitiator, to form a cross‐linked network. The precursor solution was directly applied to the wound site via injection and solidified in situ within 30 s under 405 nm visible light irradiation to form a stable cover on the lesions. This provides an efficient and precise therapeutic strategy for the dynamic repair of diabetic wounds.^[^ ^81^ ^]^

Although single stimulus‐responsive drug delivery systems have been well established, credited to advancements in biomedical engineering and nanotechnology, these systems are inadequate in addressing complex physiological barriers and multifaceted challenges imposed by a disease microenvironment within the body.^[^ ^189^, ^190^ ^]^ Single‐stimulus responses, such as pH‐ or temperature‐responsiveness, often result in an insufficient targeting efficiency or poor release controllability due to the heterogeneity in a physiological environment. Consequently, the integration of multidimensional microenvironmental changes during the delivery process (e.g., pH, redox state, and enzyme concentration) with targeted technologies to design multi‐responsive intelligent delivery systems has emerged as a pivotal strategy for achieving spatiotemporally precise and controllable delivery.^[^ ^191^ ^]^ For example, a cascading response nanoplatform (PA/ASePSD) was recently developed by Xu et al. The nanocarrier was connected to the outer layer of oxidized dextran (ox‐Dex) via pH‐sensitive Schiff base bonds. In an acidic microenvironment of the plaque (pH 5.8), the shell layer was detached to exposing the SS‐31 peptide. Subsequently, after exposure to a high ROS concentration, the selenium‐selenium bond was cleaved to completely release astaxanthin and the mitochondria‐targeting peptide SS‐31, thereby scavenging mitochondrial ROS, regulating lipid metabolism, and promoting cholesterol efflux.^[^ ^128^ ^]^ Furthermore, mesoporous polydopamine nanoparticles developed by Deng et al., which were formed through self‐polymerization reaction of dopamine, underwent ROS‐mediated polymer oxidation under high ROS conditions. The presence of numerous phenolic hydroxyl groups and quinone groups in their molecular structure endowed them with unique properties. The nanoparticles absorbed near‐infrared light at 808 nm, and the temperature at the nanoparticle‐accumulated region reached up to 47.2 °C, thereby achieving photothermal sterilization. In addition, these groups accelerated the release of SS31 through the thermal effect, thereby enhancing mitochondrial protection.^[^ ^118^ ^]^ Integrated design of these multi‐stimulus responsive nanomedicines provides new insights for precise intervention in the complex microenvironments of metabolic diseases.^[^ ^192^ ^]^

## 5 Nanomedicine‐Mediated Therapeutic Strategies for Mitochondrial Dysfunction Diseases

**Figure 6:**
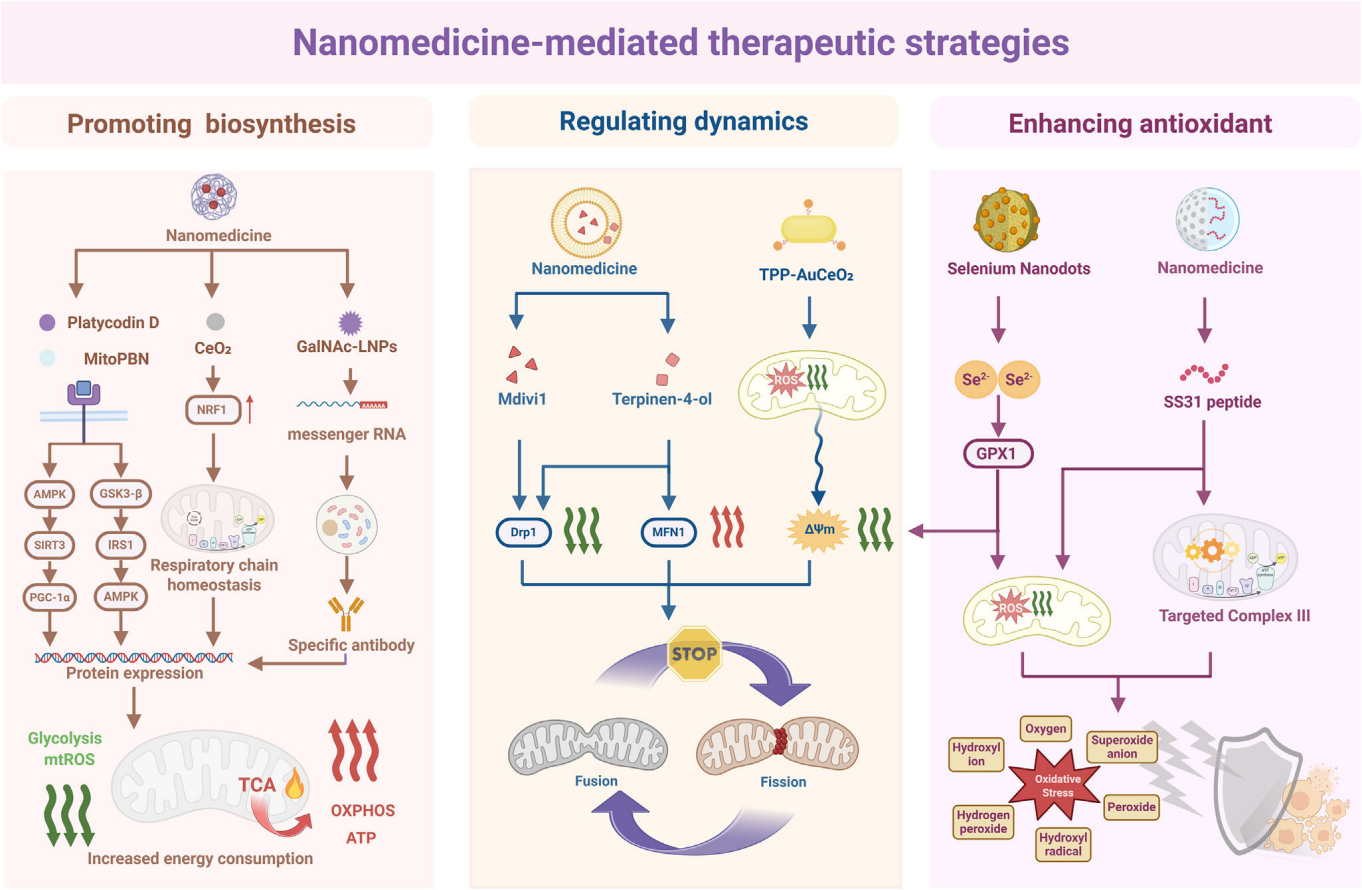
Therapeutic strategies of nanomedicines against mitochondrial dysfunction‐induced metabolic diseases. Nanomedicines synergistically intervene in mitochondrial dysfunction‐induced metabolic diseases through three mechanisms, activating biosynthetic pathways (such as PGC‐1α), regulating the balance between fission and fusion (Drp1/MFN1), and enhancing antioxidant defense (clearing ROS/repair enzymes), thereby reversing mitochondrial dysfunction in multiple dimensions. Created in https://BioRender.com.

The core pathological features of mitochondrial dysfunction include energy metabolism imbalance, oxidative stress, and disruption of kinetic homeostasis, and these features have become potential targets for nanomedicine interventions.^[^ ^168^ ^]^ In this chapter, we elaborate on a series of multidimensional strategies for precise intervention in mitochondrial dysfunction. These strategies are based on the mechanisms outlined above, including promoting biosynthesis, regulating kinetic balance, and enhancing antioxidant defense^[^ ^193^ ^]^ (**Figure** **6**).

### 5.1 Promoting Mitochondrial Biosynthesis

Enhancing mitochondrial biogenesis can be primarily achieved by activating key signaling pathways for mitochondrial biogenesis. For instance, Wu et al. developed MitoPBN‐loaded liposomes to exert an antioxidant effect in a high ROS environment by activating the AMPK/SIRT3/PGC‐1α axis, upregulating genes related to mitochondrial biogenesis, and improving the oxidative phosphorylation capacity of liver cells.^[^ ^194^ ^]^ A naturally occurring cofactor within the mitochondria, thioctic acid‐functionalized ginsenoside D, was delivered by a specific nanocarrier to selectively target the mitochondria, thereby modulating mitochondrial metabolism through the GSK3‐β/IRS1/AMPK pathway.^[^ ^117^ ^]^

Additionally, nanomedicines can deliver mitochondrial protein‐related messenger RNA (mRNA) to target tissues.^[^ ^99^, ^195^ ^]^ Zhang et al. designed galactosyl‐modified lipid nanoparticles (GalNAc‐LNPs) for the delivery of hepatocyte‐specific mRNA.^[^ ^106^ ^]^ This nanomedicine was applied to modulate mitochondrial dynamics, and it facilitated sustained translation and expression of antibody proteins targeting PGC‐1α within target cells via mRNA. This treatment process resulted in sustained upregulation of mitochondrial DNA replication genes, such as nuclear factor 1 (NRF1) and mitochondrial transcription factor A (TFAM), thereby restoring the respiratory chain activity. This mRNA delivery approach presents a compelling alternative to conventional small‐molecule activators. In a similar manner, the Au@16‐pH‐16/miR‐21 mimic nanomedicine developed by Lhamyani et al. achieved targeted delivery of miR‐21 mimics to adipocytes, upregulating the PGC‐1α signaling pathway and subsequently promoting mitochondrial biogenesis.^[^ ^60^ ^]^

Finally, it has been demonstrated that certain inorganic nanomedicines can promote mitochondrial biogenesis by directly regulating signaling pathways. A mechanistic study by Óro et al. revealed that a cerium oxide nanomedicine (CeO_2_ NPs) elicited redox‐mediated reactivation of mitochondrial biogenesis, thereby restoring mitochondrial redox homeostasis through the Ce^3+^/Ce^4+^ redox cycle. In addition, these particles were observed to upregulate components of the ETC that are mediated by NRF1. Concurrently, the mitochondrial mass experienced a significant increase, and the VDAC1 protein expression level rose by 1.8‐fold. This intervention restored the mitochondrial cristae density to a normal level.^[^ ^196^ ^]^

### 5.2 Regulation of Mitochondrial Dynamics

Mitochondrial dynamics imbalance is a core mechanism of mitochondrial dysfunction in metabolic diseases, manifested as fragmentation and fusion disorders caused by excessive division. By targeting proteins related to division/fusion, such as dynamin‐related protein 1 (Drp1) and Mitofusin1 (MFN1), the integrity of the mitochondrial network can be restored.^[^ ^197^ ^]^

The regulation of mitochondrial dynamics is centered on the maintenance of a balance between the processes of mitochondrial fission and fusion.^[^ ^198^ ^]^ Mdivi1, a mitochondrial fission inhibitor, can be utilized to target Drp1 (dynamin‐related protein 1), thereby impeding excessive mitochondrial fission. Furthermore, the suppression of Drp1‐mediated Bax protein translocation to the mitochondria has been shown to reduce CytC leakage into the cytoplasm, thereby inhibiting mitochondrial outer membrane permeabilization (MOMP)‐induced apoptosis.^[^ ^199^ ^]^ Terpinen‐4‐ol is a natural small‐molecule compound extracted from plant essential oils. It possesses a variety of pharmacological activities, including antioxidant, anti‐inflammatory, antibacterial, and mitochondrial protective effects. A terpinen‐4‐ol‐loaded TPP‐modified targeted nanomedicine was shown to inhibit the expression of Drp1 and upregulate the expression of mitochondrial fusion protein MFN1, resulting in the restoration of the mitochondrial membrane potential and effective reestablishment of a dynamic balance between mitochondrial fission and fusion.^[^ ^150^ ^]^ Furthermore, inorganic AuCeO_2_ nanoparticles were reported to indirectly influence a kinetic equilibrium by regulating the mitochondrial respiratory chain activity. It has been discovered that a decrease in the mitochondrial membrane potential (ΔΨm) as a pivotal signal can trigger mitochondrial hyperproliferation. This process is achieved by promoting the recruitment and activation of Drp1 protein within the mitochondria. Consequently, the restoration of the damaged mitochondrial membrane potential emerges as a promising strategy to impede excessive fission in a pathological context. Gutiérrez‐Carcedo et al. developed a mitochondrial‐targeting nanoparticle, TPP‐AuCeO_2_, for restoring ΔΨm by alleviating oxidative stress, thereby indirectly influencing the kinetic equilibrium. TPP‐AuCeO_2_ accumulated selectively in the mitochondrial matrix through cationic selectivity of triphenylphosphine in the nanoparticle, and the nanoparticle cleared excess ROS through its Ce^3^^+^/Ce^4+^ redox cycle. By neutralizing ROS, TPP‐AuCeO_2_ helped restore the mitochondrial membrane potential, thus indirectly regulating mitochondrial dynamics, including inhibiting Drp1‐mediated excessive fission. Furthermore, the antioxidant effect of TPP‐AuCeO_2_ nanoparticles may upregulate the expression of NRF1/nuclear factor E2‐related factor 1 (NFE2L1), thereby exerting an additional effect on mitochondrial biogenesis and antioxidant defense.^[^ ^59^ ^]^

### 5.3 Enhancing Antioxidant Defense

The augmentation of the antioxidant defense system primarily safeguards the mitochondrial function by eradicating ROS or amplifying the activity of endogenous enzymes.^[^ ^200^ ^]^ Smart drug delivery systems (DDSs) can be designed by equipping multifunctional nanocarriers with targeting moieties to achieve multidimensional interventions against mitochondrial oxidative damage. For instance, SENDs have been shown to release selenium ions through ROS response, which activate GPX1 and repair the mitochondrial membrane potential. In addition, SENDs can restore the mitophagy function, thereby reducing oxidative stress by eliminating damaged mitochondria. Furthermore, SENDs have been demonstrated to relieve endoplasmic reticulum stress (ERS) by promoting the expression of key transcription factors, such as PDX1 and MAFA. The application of SENDs led to significant alleviation in pancreatic β‐cell apoptosis.^[^ ^171^ ^]^ In the context of intervention strategies for targeting oxidative stress in various metabolic diseases, nanomedicines can be customized to deliver therapeutic agents based on differential therapeutic mechanisms for oxidative damage characteristics of target organs. For example, a mesoporous dopamine nanomedicine loaded with SS31 was developed for the treatment of diabetes. At the structural level, mesoporous dopamine, with its *π–π* conjugated framework and electron‐delocalized property, efficiently adsorbed and removed ROS within the mitochondria. At the functional repair level, the SS31 peptide targeted respiratory chain complex III to restore the integrity of the electron transport chain by reconfiguring its spatial conformation. This synergistic strategy of combining physical clearance and biochemical repair effectively reversed mitochondrial dysfunction under a high‐glucose condition.^[^ ^118^ ^]^ To treat NAFLD, cerium dioxide nanoparticles (CeO_2_ NPs) were employed with a dual‐blocking mechanism to target both oxidation and peroxidation processes. The CeO_2_ NPs displayed dynamic antioxidant activity, and the reversible redox cycle of Ce^3^⁺/Ce⁴⁺ helped the neutralization of direct oxidants, including ·OH and H_2_O_2_. In terms of product clearance, surface oxygen vacancy defects in the CeO_2_ NPs specifically captured lipid peroxidation end products, such as malondialdehyde (MDA), through coordination interactions. This dual‐track strategy, which eliminated free radicals by consuming their sources and removed toxic byproducts, successfully blocking the malicious cycle of “oxidative stress‐lipid peroxidation”.^[^ ^121^ ^]^

Simultaneously achieving antioxidant, anti‐inflammatory, and metabolic regulation has been explored for the treatment of metabolic diseases through specific nanodrug design. The nanodrug design principles for targeted modification, responsive release, and functional material adaptation can be tailored for disease‐specific oxidative damage mechanisms. These principles establish a scalable technical framework for developing next‐generation mitochondria‐targeting nanomedicines to enhance the antioxidant defense system.

## 6 The Application of Mitochondria‐Targeting Nanomedicines in Metabolic Diseases

In the following section, we present case studies on the application of nanomedicines in the treatment of obesity, atherosclerosis, diabetes, and non‐alcoholic fatty liver disease. We examine specific pathological mechanisms and illustrate design principles of nanomedicines, which provide insights into new designs of nanomedicines or their applications in various metabolic diseases.

### 6.1 Application in Obesity

Adipose tissue is not only the main site of energy storage, but also a core organ for regulating the metabolic balance in the body.^[^ ^201^ ^]^ In patients with obesity, there is an excessive enlargement in adipocytes, which leads to an increase in the lipid storage capacity. This, in turn, triggers lipid overflow that results in an elevated level of circulating free fatty acids (FFAs). These FFAs interfere with insulin signaling pathways in peripheral tissues.^[^ ^202^, ^203^ ^]^ These pathological changes become the root causes for the development of type 2 diabetes.

**Figure 7:**
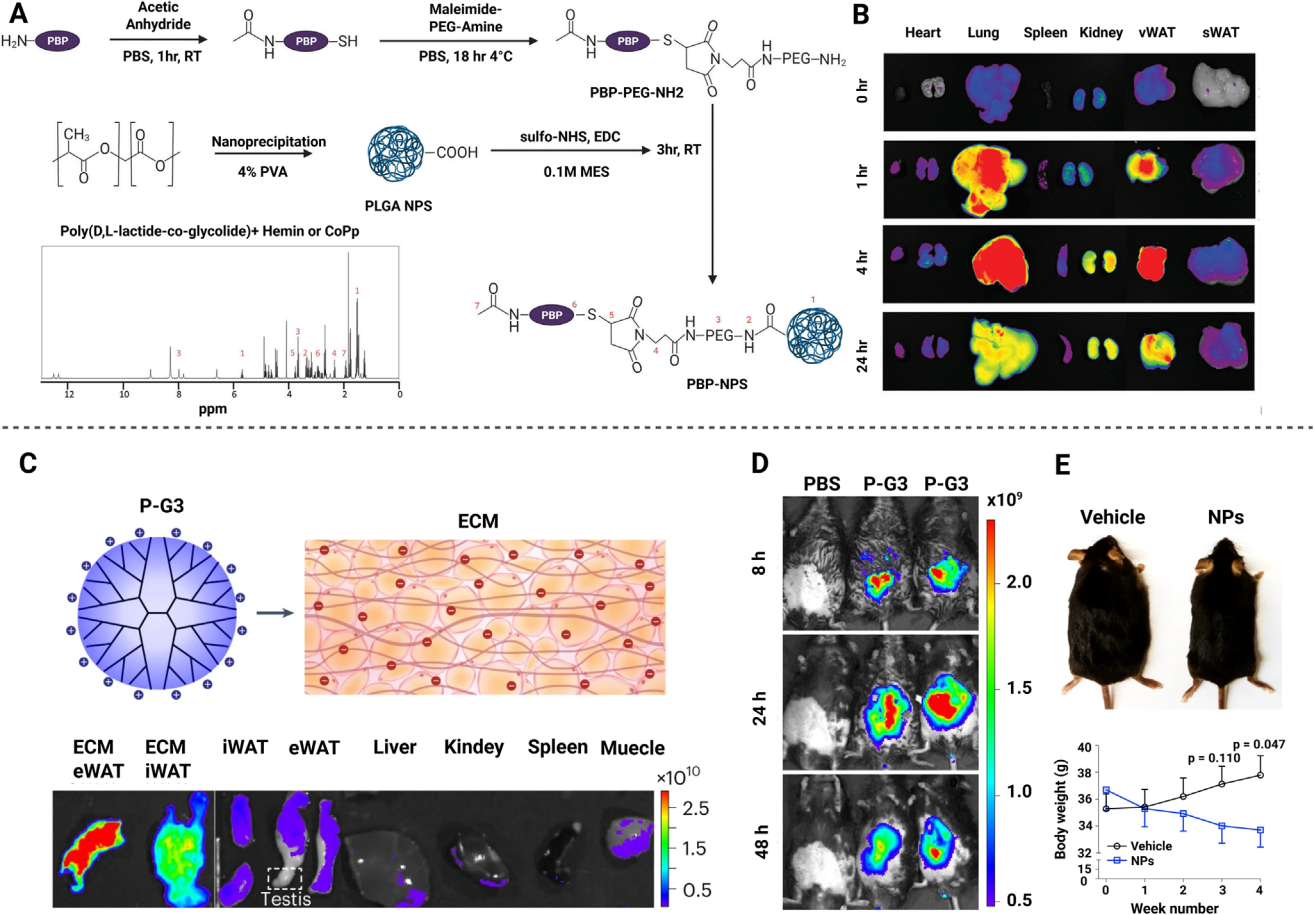
Engineered nanomedicines for targeted intervention of localized fat tissue in patients with obesity. A) Schematic diagram of prohibitin‐binding peptide (PBP)‐conjugated PLGA nanoparticles (PBP‐NPs) encapsulating heme oxygenase‐1 (HO‐1) inducers (Hemin or CoPP) and the in vivo study design. This adipose‐tissue‐addressable nanoplatform exploits prohibitin overexpression on mature adipocytes and adipose tissue macrophages to enrich cargoes in visceral WAT and trigger mitochondrial thermogenic programs. B) *Ex vivo* biodistribution image of intravenously injected Cy5.5‐loaded PBP‐PLGA nanoparticles (PBP‐NPs) in a high‐fat/high‐fructose diet‐induced NASH mouse model, demonstrating preferential hepatic accumulation consistent with fatty‐liver targeting. Reproduced with permission.^[^ ^204^ ^]^ Copyright 2022, Wiley‐VCH GmbH. C) Schematic diagram of third‐generation polyamide amine (P‐G3), a visceral fat‐targeting nanocarrier via electrostatic interaction with overexpressed anionic receptors to achieve selective accumulation in visceral fat tissue. D) In vivo imaging of P‐G3 signals at 8, 24, and 48 h post‐intraperitoneal injection. E) Representative images of mice after 6 weeks of P‐G3 treatment and their weight curves prior to metabolic measurement interference. Reproduced with permission.^[^ ^205^ ^]^ Copyright 2022, Springer Nature Limited. Created in https://BioRender.com.

In the context of obesity‐related metabolic disorders, the development of mitochondria‐targeting nanomedicine has emerged as a promising therapeutic approach. Impaired mitochondrial function leads to a reduction in mitochondrial biogenesis and a decrease in the mitochondrial DNA content, as well as a reduction in the rate of fatty acid β‐oxidation. These changes in adipocyte metabolic pathways include lipogenesis, lipolysis, fatty acid esterification, and adipocyte‐derived adiponectin production.^[^ ^1^ ^]^ In this setting, Hong & Kim developed prohibitin binding peptide (PBP‐NPs) that actively target prohibitin‐rich adipocytes and adipose macrophages to induce HO‐1 and thereby activate a coordinated SIRT1/AMPK → PGC‐1α/PRDM16/PPARγ → UCP1 thermogenic cascade (**Figure** **7**A,B). In diet‐induced obese mice, PBP‐NPs (Hemin or CoPP) selectively accumulated in visceral WAT, reduced body weight (≈20%) without affecting food intake, improved insulin sensitivity, lowered circulating FFAs/TG/LDL, and promoted mitochondrial biogenesis (e.g., TFAM, UCP1) and multilocular “brown‐like” morphology; simultaneously, they re‐polarized ATMs from M1 to M2, decreasing IL‐1β/IL‐6/TNF‐α. Notably, in a NASH model, the same particles dual‐targeted fatty liver, suppressed SREBP1c/FASN, elevated hepatic PGC‐1α/PPARα, reduced triglyceride burden, and attenuated apoptosis/fibrosis (↓ALT/AST, ↓α‐SMA/hydroxyproline), highlighting a browning‐driven, immunometabolic reprogramming route to mitigate obesity and complications.^[^ ^204^ ^]^ Another representative strategy is to achieve localized drug release from nanomedicines for signal pathway regulation. For example, PLGA nanoparticles loaded with dibenzazepine (DBZ) sustainably release DBZ after delivery to adipose tissue, thereby upregulating brown adipose tissue‐associated gene expression by inhibiting the Notch signaling pathway.^[^ ^132^ ^]^

In comparison with conventional pharmaceutical agents, nanocarriers exhibit distinctive advantages in enhancing the efficiency of drug delivery and surmounting delivery barriers. These advantages can be attributed to the effects of their nanoscale size and functional moieties on the surface. A notable example is to improve the water solubility of resveratrol (R) via nanoscale encapsulation with lipid nanocarriers (R‐nano) and liposomes (R‐lipo). The water solubility of resveratrol was enhanced by 25‐fold. In an in vitro white adipocyte model, R‐lipo and R‐nano displayed a 20% and 25% increase in the R content, respectively. Both R‐lipo and R‐nano enhanced brown adipocyte differentiation by activating the PPARγ pathway and inducing the expression of UCP1 mRNA. These results confirmed that the formation of nanomedicine can significantly improve drug bioavailability ^[^ ^206^ ^]^ and overcome the delivery bottleneck of traditional dosage forms to enhance the intracellular uptake efficiency. More notably, preclinical studies have shown that the Pluronic‐modified leptin nanomedicine (Lep(ss)‐P85) can bypass the BBB to increase the accumulation of the delivered drug in the brain by 60%, thereby offering a new treatment option for leptin‐resistant obesity.^[^ ^173^ ^]^ Additionally, Wan et al. developed a third‐generation nanodrug P‐G3 based on polyamide and its cholesterol‐modified derivative, lipophilic P‐G3‐Chol (5) nanoparticles (NPs) (Figure 7C–E). In vivo experiments demonstrated that after IP injection of Cy5‐labeled P‐G3, the fluorescence intensity in visceral fat was 3.5 times higher than that in subcutaneous fat (epididymal white adipose tissue (eWAT) 1.2 × 10⁹ versus inguinal white adipose tissue (iWAT) 3.5 × 10⁸ photons s^−1^ cm^−^^2^ sr^−1^). There were 32 amino groups on the surface of the nanodrug, and they specifically bound to the negatively‐charged extracellular matrix of adipose tissue. In a high‐fat diet mouse model, treatment with P‐G3‐Chol (5) NPs in diet‐induced obese mice with established obesity resulted in a 15% reduction in body weight, a 45% reduction in fat mass, and a 50% reduction in the eWAT depot size, while improving glucose tolerance.^[^ ^205^ ^]^ These examples demonstrate that the multifunctional design of nanocarriers can be specifically tuned for different pathological stages of obesity (such as fat browning disorders and central leptin resistance).

**Figure 8:**
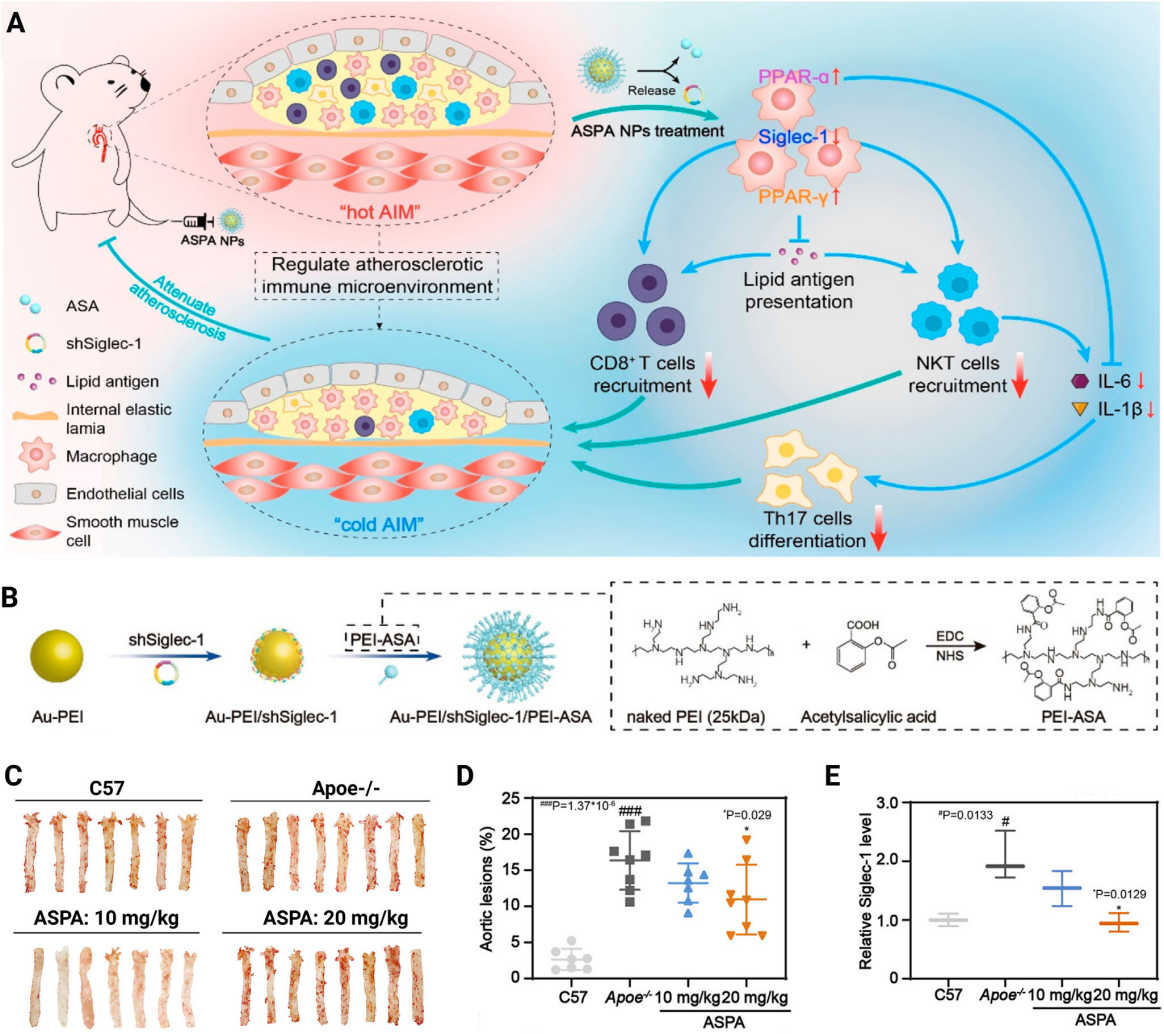
A “heat‐to‐cold” strategy for ASPA nanoparticles to alleviate atherosclerosis by reshaping AIM. A) Mechanisms of action of ASPA nanoparticles in the treatment of atherosclerosis. B) Chemical composition of ASPA nanoparticles and their synthesis route. C) Representative photographs of aortic cross‐sections stained with ORO in mice after different treatments. D) Quantitative analysis of aortic lesion areas. E) Quantitative analysis of the relative Siglec‐1 expression level in aortic root sections. Reproduced with permission.^[^ ^84^ ^]^ Copyright 2022, American Chemical Society. Created in https://BioRender.com.

In addition to directly regulating adipocyte metabolism, meticulously designed nanomedicines have the potential to impede the progression of obesity by disrupting the detrimental cycle between chronic inflammation and mitochondrial dysfunction. The infiltration of M1‐type macrophages into adipose tissue has been observed to result in the secretion of pro‐inflammatory cytokines, such as TNF‐α and IL‐6.^[^ ^207^ ^]^ These cytokines can hinder the mitochondrial β‐oxidation capacity of adipocytes and induce serine phosphorylation of insulin receptor substrate (IRS‐1), consequently leading to the impaired insulin signaling pathway.^[^ ^61^, ^208^ ^]^ This process exhibits a comparable pathophysiological mechanism to that of ROS bursts in myocardial reperfusion injury. Overabundance in inflammatory factors can be considered as a form of “metabolic reperfusion injury”, thereby continuously disrupting the mitochondrial homeostasis of adipocytes. Mitochondria‐targeting nanomedicines can be developed to targeting this insulin signaling pathway. For example, nanodrugs constructed using the lipophilic cationic dye IR‐16 can target the mitochondria of adipose tissue macrophages. Treatment of the nanomedicine resulted in an increase in the ATP/ADP ratio and a restoration of the complex I activity.^[^ ^208^ ^]^

In summary, nanomedicines primarily regulate fat metabolism in obesity treatment through targeted delivery and functionalized design. The utilisation of nanocarriers, such as gold nanoparticles or polymers, has been demonstrated to enhance the efficiency of drug accumulation in adipose tissue. The mechanisms by which this occurs include the promotion of energy expenditure by inducing the conversion of white adipose tissue to brown adipose tissue, the scavenging of reactive oxygen species, and the restoration of mitochondrial function. Concurrently, the nanoscale properties enhance the solubility of drugs and facilitate the permeation of biological barriers (e.g., the BBB). By modulating inflammatory signalling pathways in order to alleviate insulin resistance, these approaches achieve comprehensive therapeutic effects encompassing weight reduction and metabolic improvement. A summary of representative nanomedicines used for obesity treatment, including their types, materials, targeting mechanisms, and therapeutic actions (**Table** **1**).

**Table 1:** Different types of nanomedicines used for obesity treatment.

Year	Types of nanomedicines	Nanomaterials	Drug loading	Target molecules	Targeting mechanisms	Stimuli‐response mechanisms	Diseases treated	Mechanisms of disease treatment
2018^[^ ^201^ ^]^	Lipid nanocarriers, liposomes	Soy L‐α‐phosphatidylcholine, cholesterol	Trans‐Resveratrol	–	Passive targeting	–	Obesity	Activation of the PPARγ signaling pathway promotes the expression of UCP1, which promotes the transformation of white adipocytes to beige adipocytes; increases the expression of beige adipocyte markers (e.g., Ucp1, Cd137) and decreases the expression of white adipocyte markers (e.g., Igfbp3)
2022^[^ ^205^ ^]^	Hybrid nanoparticles	Polyamidoamine‐G3	–	Visceral fat tissue	Passive targeting	–	Obesity	Regulation of the mTOR signaling pathway to inhibit lipid synthesis in adipocytes, thereby reducing adipocyte hypertrophy
2021^[^ ^208^ ^]^	Inorganic nanoparticles	heptamethine cyanine dye	–	Mitochondria in adipose tissue macrophages	Passive targeting	–	Obesity	Increases ATP and ADP/ATP ratios, increases mitochondrial complex levels and activity, enhances oxidative phosphorylation in adipose tissue macrophages, and inhibits M1‐type (pro‐inflammatory) activation of macrophages
2024^[^ ^61^ ^]^	Inorganic nanoparticles	Gold nanoparticles	Carnitine	Adipose tissue, CNS	Passive targeting	–	Obesity and its triggered brain damage (e.g., oxidative damage to the hippocampus, abnormal energy metabolism in the striatum)	Inhibits oxidative stress; reduces levels of pro‐inflammatory cytokines (e.g., IL‐1β, TNF‐α); improves mitochondrial respiratory chain complex (e.g., complexes I and IV) activity and restores ATP production
2024^[^ ^60^ ^]^	Inorganic nanoparticles	Gold nanoparticles	Mir‐21 mimics	Adipose tissue	Passive targeting	–	Obesity	Inhibits oxidative stress; reduces levels of pro‐inflammatory cytokines (e.g., IL‐1β, TNF‐α); improves mitochondrial respiratory chain complex (e.g., complexes I and IV) activity and restores ATP production
2012^[^ ^148^ ^]^	Polymers	PLGA	Lonidamine, α‐tocopherol succinate, curcumin, 2,4‐dinitrophenol	Mitochondria	Active targeting, TPP modification	–	Cancer, AD, obesity	Uncoupling mitochondrial oxidative phosphorylation to increase energy expenditure and decrease fat accumulation
2013^[^ ^111^ ^]^	Polymers	Nanoparticle systems targeting endothelial cells (PTNP)	Cytc	Endothelial cells in WAT	Active targeting, targeted peptides (CKGGRAKDC)	–	Obesity	Induces endothelial cell apoptosis, thus destroying the vascular structure of adipose tissue and inhibiting adipose tissue growth and hypertrophy; activates caspase‐9 and triggers cell apoptosis
2016^[^ ^112^ ^]^	Polymers	PLGA‐b‐PEG	Rosiglitazone, prostaglandin E2 analog (16,16‐dimethyl PGE2)	Vascular endothelial cells of WAT	Active targeting, targeting peptides (iRGD and P3)	–	Obesity	Activation of PPARγ, up‐regulation of the expression of BAT markers such as UCP1 and CIDEA, and promotion of the conversion of WAT to BAT
2017^[^ ^132^ ^]^	Polymers	PLGA	Dibenzazepine	Inguinal WAT	Passive targeting	–	Obesity	Inhibition of the Notch signaling pathway promotes the conversion of WAT to beige adipose tissue; up‐regulation of the expression of beige adipocyte markers such as Ucp1 and Cidea promotes the conversion of WAT to beige adipose tissue
2014^[^ ^173^ ^]^	Polymers	Pluronic P85 (triblock copolymer PEO‐PPO‐PEO)	Recombinant leptin	Hypothalamus and other brain regions (target site of action for leptin)	Passive targeting	Glutathione response	Leptin‐resistant obesity	Activates leptin receptors in the hypothalamus to suppress appetite and enhance energy expenditure

### 6.2 Application in Atherosclerosis

The development of therapeutic strategies for targeting the regulation of oxidative stress, inflammatory responses, and autophagy defects has emerged as a prominent area of research interest in the field of atherosclerosis. Precision and multifunctionality of nanomedicines are two essential features for the implementation of these strategies.

As an mTOR pathway inhibitor, rapamycin has been demonstrated to exert multifaceted effects in the treatment of atherosclerosis by suppressing inflammatory responses, regulating cell proliferation, and promoting plaque stabilization. The optimization of its nanomedicines has led to substantial improvements in their targeting and efficacy.^[^ ^209^ ^]^ For instance, the pH‐responsive hyaluronic acid nanoparticles (HRRNPs) developed by Cheraga et al., as mentioned in Section 4.3.1. At a pH of 5.2, simulating the plaque condition, the cumulative release rates of ATR and the anti‐atherosclerotic drug RAP reached ≈80.3% and 94.9%, respectively, within 48 h.^[^ ^109^ ^]^ Furthermore, Fang et al. developed integrin αvβ3‐targeted and cathepsin K‐responsive nanoparticles (T/RNPs) through self‐assembly of a targeting polymer (PLGA‐PEG‐c(RGDfC)) and a sensitive polymer (PLGA‐Pep‐PEG) containing a CTSK peptide sequence that is a cathepsin K substrate. Rapamycin was released from the nanodrug in the plaque upon activation by the CTSK enzyme, significantly inhibiting the uptake of oxidized low‐density lipoprotein and foam cell formation.^[^ ^174^ ^]^

In addition to single‐target strategies, multi‐target and responsive nanomedicines that harness the plaque microenvironmental features can enhance the drug delivery efficiency and therapeutic efficacy. Chen et al. constructed two ROS‐responsive nanomicelles, N‐Acetylneuraminic acid‐Chondroitinsulfate‐S‐allyl‐L‐cysteine‐Ferrocene and N‐Acetylneuraminicacid‐Chondroitinsulfate‐Thioketone‐4‐methoxyphenylthiourea (SCTM), with a targeting moiety of N‐acetylneuraminic acid (SA) and chondroitin sulfate (CS), respectively. Different H_2_S donors were encapsulated into two micelles, S‐allyl‐L‐cysteine (SAC) in the SACF micelle, while 4‐methoxybenzothiourea (4‐MTC) in the SCTM micelle. After the micelles reached the complex microenvironment of plaques, SA targeted the E‐selectin receptor on endothelial cells, while CS targeted the CD44 receptor, achieving dual receptor targeting and synergistic delivery of RAP and H_2_S donors. In the ApoE^−^/^−^ mouse model, the plaque area in the SCTM@RAP group decreased to 4.89%, significantly lower than that in the free rapamycin group (19.42%).^[^ ^210^ ^]^ Bionic delivery systems overcome the limitations of traditional targeted delivery systems. For example, Wang et al. developed PLGA particles loaded with RAP and coated with macrophage membranes (MM), to construct biomimetic nanoparticles (MM/RAPNPs), thus prolonged the circulation time of the nanomedicine in the bloodstream (with 15% retaining after 48 h) and increasing the rapamycin concentration in plaques by approximately threefold compared to the free drug group.^[^ ^92^ ^]^

Statins (e.g., simvastatin and atorvastatin), classic HMG‐CoA reductase inhibitors, have been shown to enhance lipid‐lowering, anti‐inflammatory, and plaque‐stabilizing effects in the treatment of atherosclerosis through an optimized nanomedicine system. A nimetopril poly‐lactic acid‐hydroxyethyl acrylate copolymer nanodrug was shown to inhibit the monocyte chemotactic protein‐1 (MCP‐1)/CCR2 signaling pathway, thereby reducing the recruitment of inflammatory monocytes (Ly‐6Chigh) to plaques.^[^ ^72^ ^]^ Furthermore, polypropylene sulfide (PPS) in a ROS‐responsive micelle could be decomposed in a high ROS environment. Decomposition of PPS allowed simultaneous release of simvastatin and consumption of ROS, thereby inhibiting the expression of inflammatory factors (such as IL‐6 and TNF‐α) driven by the NF‐κB pathway.^[^ ^94^, ^211^ ^]^ A complex formed by methyl‐β‐cyclodextrin and simvastatin had a core‐shell structure after encapsulation in phospholipids. The complex not only removed plaque cholesterol through host‐guest interaction between cyclodextrin and cholesterol, but also released simvastatin in the cholesterol‐rich area to exert its effect. The synergistic action of cholesterol reduction and simvastatin inhibition reduced the plaque lipid content by 52% and inhibited the transformation of macrophages into foam cells.^[^ ^212^ ^]^ Additionally, an atorvastatin delivery system based on liposome technology developed by Duivenvoorden et al. has demonstrated unique advantages. Recombinant high‐density lipoprotein (rHDL) nanoparticles loaded with liposome‐modified atorvastatin with targeted peptides were used to specifically recognize endothelial cell surface receptors at plaque sites. The targeted liposomal atorvastatin effectively inhibited the proliferation and migration of vascular smooth muscle cells.^[^ ^213^ ^]^

Another pivotal strategy is to redefine the atherosclerotic immune microenvironment (AIM). Zhou et al. designed a pH‐responsive charge‐reversible nanomedicine (ASPA NPs) for delivering shRNA targeting Siglec‐1 (shSiglec‐1) and an aspirin derivative (PEI‐ASA). ASPA NPs underwent hydrolysis within an acidic endosomal environment, resulting in the release of shSiglec‐1, which achieved gene silencing to inhibit the interaction between macrophages and CD8+ T/NKT cells, thereby preventing immune cell infiltration. Additionally, ASA activated the PPAR‐α/γ pathway to promote cholesterol efflux and suppresses the secretion of inflammatory factors IL‐6 and IL‐1β produced by macrophages. The synergistic action of significantly reducing two pro‐inflammatory factor IL‐6 and IL‐1β successfully reprogrammed a pro‐inflammatory “hot AIM” to an anti‐inflammatory “cold AIM”, effectively alleviating atherosclerotic lesions in mouse models (**Figure** **8**).^[^ ^84^ ^]^

Nanotechnology‐aided intervention strategies for targeting mitochondrial metabolic disorders may offer a novel therapeutic perspective for cardiovascular complications associated with atherosclerosis. As previously mentioned, mitochondrial fission inhibitors, including Mdivi1 and cyclosporine A can be encapsulated in PLGA nanoparticles. The Mdivi1‐incorporated nanoparticles were internalized by damaged myocardial cells via an oxidative stress‐responsive mechanism, and they specifically accumulate in these damaged cells. The Mdivi1‐containing nanodrug targeted myocardial mitochondria, inhibited Drp1‐mediated mitochondrial fission, and reduced the myocardial infarction area by 30% in an ischemia‐reperfusion model.^[^ ^199^ ^]^ The cyclosporine A‐containing nanomedicine was found to inhibit the opening of the mPTP, maintain the mitochondrial membrane potential, reduce myocardial cell apoptosis, and improve the cardiac function.^[^ ^214^ ^]^

A nanoplatform that integrates multimodal therapy and imaging has been explored for integrated diagnosis and treatment, and this nanoplatform opens a door for precision medicine in atherosclerosis.^[^ ^215^ ^]^ Photothermal‐responsive copper sulfide nanoparticles were conjugated with TRPV1 monoclonal antibodies on the surface of nanoparticles. The conjugated nanoparticles bound to TRPV1 ion channels on the membranes of VSMCs, achieving targeted accumulation at plaque sites. Targeted accumulation of the nanoparticles triggered a local thermal effect under near‐infrared light irradiation, activating the AMPK pathway and promoting autophagy, which resulted in a reduction in foam cell formation. In addition, the nanoparticles offered the capacity of MR imaging, which could offer MRI‐guided therapy. The paramagnetic copper sulfide core significantly enhanced T2‐weighted signal contrast.^[^ ^216^, ^217^ ^]^ To combine mechanical force regulation and visualization‐guided therapy, Hou et al. developed FPD@CD nanoparticles with a hydrophobic core/hydrophilic shell structure formed by PLGA and PEG, respectively. Under LIFU stimulation, FPD@CD underwent a liquid‐to‐gas phase transition termed as acoustic droplet vaporization (ADV), generating mechanical stress via the cavitation effect. The mechanical stress directly disrupted the lysosomal membranes of macrophages, targeting the clearance of inflammatory cells in plaques. Additionally, an embedded DiR fluorescent probe in the inner core was used for deep‐penetration imaging in the near‐infrared region II (NIR‐II), allowing dynamic tracking of targeted aggregation of the nanodrug within plaques and the resolution of inflammation post‐treatment.^[^ ^73^ ^]^ Additionally, Xu et al. developed a multifunctional cascade‐responsive nanoplatform, PA/ASePSD. The hydrophobic core was also loaded with a photoacoustic probe, PMeTPP‐MBT, while the hydrophilic PEG shell was covalently bound to a mitochondria‐targeting peptide SS‐31. The amino groups on the nanoparticle surface (e.g., PEG/SS3) were coupled with the aldehyde groups of oxidized dextran (ox‐Dex) via the Schiff base reaction, resulting in an outermost pH‐responsive layer. This structural design allowed cascade‐release of encapsulated therapeutic and imaging agents. In a weakly acidic environment of plaques (pH ≈5.8), the pH‐responsive layer (ox‐Dex) was subjected to hydrolysis and became detached to partially expose the SS‐31 peptide. Subsequently, in response to a high ROS concentration in plaques, the diselenium bond was cleaved to simultaneously release astaxanthin to scavenge ROS in macrophages and the SS‐31 peptide to repair the mitochondrial membrane potential. The concurrently released PMeTPP‐MBT probe bound to plaque cholesterol crystals, allowing real‐time assessment of the lipid burden and oxidative stress via photoacoustic imaging, thereby achieving precision therapy guided through in situ and real‐time assessment.^[^ ^128^ ^]^

In summary, nanomedicine for treating atherosclerosis primarily employs multiple precision strategies. First, nanoparticle carriers that are responsive to plaque microenvironments (such as pH, reactive oxygen species, or specific enzymes) are utilized to achieve targeted drug release. The process in question has been shown to suppress inflammation, reduce lipid deposition, and stabilize plaques. Second, the circulation time is extended, and the enrichment at the lesion site is enhanced through biomimetic design or surface functionalization. Thirdly, multi‐pathway synergistic therapies, such as immune microenvironment remodeling and mitochondrial function regulation, are integrated. In conclusion, the integration of diagnostic imaging with therapeutic functions has been shown to result in a synergistic therapy that is both visualized and precise. A summary of representative nanomedicines used for atherosclerosis treatment, including their types, materials, targeting mechanisms, and therapeutic actions (**Table** **2**).

**Table 2:** Different types of nanomedicines used for the treatment of atherosclerosis.

Year	Types of nanomedicines	Nanomaterials	Drug loading	Targeting mechanisms	Stimuli‐response mechanisms	Diseases treated	Mechanisms of disease treatment
2021^[^ ^94^ ^]^	Bionic micelle	Polyglycidyl methacrylate‐polypropylene sulfide (PGED‐PPS)	Simvastatin	Passive targeting	ROS response, shear stress response	Atherosclerosis	Reduces oxidative stress, anti‐inflammatory and antioxidant
2021^[^ ^218^ ^]^	Bionic polymers	RBC membrane‐PMMP	Prednisolone (loanword)	Passive targeting; active targeting, erythrocyte membrane mimicry	ROS response, lipid‐specific response	Atherosclerosis	Inhibition of macrophage activation and inflammatory responses
2022^[^ ^89^ ^]^	Biomimetic nanoparticles	Platelet membrane‐encapsulated mesoporous silica nanoparticles	Anti‐CD47 antibody	Active targeting, platelet membrane bionics	–	Atherosclerosis	Inhibition of the CD47‐SIRPα signaling pathway and deregulation of macrophage phagocytosis
2021^[^ ^92^ ^]^	Biomimetic nanoparticles	Macrophage membrane‐encapsulated biomimetic nanoparticles	Rapamycin	Active targeting, integrin α4β1/VCAM‐1 recognizes VCAM‐1 receptor, passive targeting	–	Atherosclerosis	Inhibition of macrophage secretion of inflammatory factors such as TNF‐α and IL‐6; inhibition of vascular smooth muscle cell and macrophage proliferation
2022^[^ ^84^ ^]^	Hybrid nanoparticles	Gold‐polyethyleneimine (Au‐PEI) nanoparticles with surface‐modified polyethyleneimine‐aspirin (PEI‐ASA) polymers	Short hairpin RNA against Siglec‐1, aspirin	Passive targeting; active targeting, CD68⁺macrophages	pH response	Atherosclerosis	Inhibition of Siglec‐1 expression; activation of PPAR‐α and PPAR‐γ pathways; inhibition of inflammatory factors (e.g., IL‐6 and IL‐1β) produced by macrophages
2020^[^ ^219^ ^]^	Hybrid nanoparticles	Fe_3_O_4_, PDA, cyclodextrin, PEG, polyethyleneimine	Rapamycin	Passive targeting; active target, profilin‐1 antibody	pH response	Atherosclerosis	Inhibition of vsmcs proliferation and migration
2018^[^ ^211^ ^]^	Micelle	Polyethylene glycol‐Polypropylene sulfide (PEG‐PPS)	Andrographolide	Passive targeting	ROS response	Atherosclerosis	Blockade of the NF‐κB signaling pathway and inhibition of inflammatory factor expression (e.g., IL‐6 and MCP‐1)
2023^[^ ^110^ ^]^	Micelle	Low molecular weight heparin‐unsaturated fatty acid conjugate (LMWH‐uFA)	Rapamycin	Active targeting, low‐density lipoprotein receptor	–	Atherosclerosis	Competitive binding to P‐selectin inhibits monocyte recruitment, thereby suppressing early vascular inflammation; reduces ROS levels and inflammatory factor secretion in plaques
2022^[^ ^210^ ^]^	Micelle	Copolymer micelles modified with *N*‐acetylneuraminic acid and chondroitin sulfate	Rapamycin	Active targeting, E‐selectin receptor; CD44 receptor	ROS response	Atherosclerosis	Inhibits leukocyte‐mediated inflammation, increases anti‐inflammatory cytokines, decreases ROS levels, and protects vascular endothelium; inhibits mTOR pathway
2014^[^ ^72^ ^]^	Polymers	PLGA	Pitavastatin	Passive targeting	–	Atherosclerotic plaque	Inhibition of monocyte chemotactic protein‐1 (MCP‐1)/CCR2 signaling pathway
2016^[^ ^220^ ^]^	Polymers	PLGA	Pioglitazone	Passive targeting	–	Atherosclerotic plaque	Activation of PPARγ induces differentiation of monocytes in an anti‐inflammatory direction (M2‐type macrophages)
2016^[^ ^221^ ^]^	Polymers	PLGA	Irbesartan	Passive targeting	–	Myocardial ischemia‐reperfusion injury	Activation of PPARγ inhibits recruitment of inflammatory monocytes (Ly6ChighCCR^2+^) to ischemic myocardium
2016^[^ ^199^ ^]^	Polymers	PLGA	Mitochondrial division inhibitor 1	Passive targeting; Mitochondrial targeting/mitochondrial membrane potential dependent	–	Ischemia‐reperfusion injury due to acute myocardial infarction	Blockade of Drp1‐mediated mitochondrial division and reduction of Bax protein translocation to mitochondria; inhibition of MOMP (mitochondrial outer membrane permeabilization)
2016^[^ ^222^ ^]^	Polymers	PLGA	Pitavastatin	Passive targeting	–	Ischemia‐reperfusion injury due to acute myocardial infarction	Activation of PI3K/Akt pathway; inhibition of monocyte‐mediated inflammatory response
2016^[^ ^214^ ^]^	Polymers	PLGA	Cyclosporine A	Passive targeting; Mitochondrial targeting/mitochondrial membrane potential dependent	ROS response	Ischemia‐reperfusion injury due to acute myocardial infarction	Inhibition of mPTP opening prevents mitochondrial membrane potential collapse and reduces cytochrome c release, thereby inhibiting cardiomyocyte apoptosis and necrosis
2013^[^ ^223^ ^]^	Polymers	PLGA	Coq_10_, VEGF	Passive targeting	–	Myocardial Ischemia	Stimulates endothelial cell proliferation and migration, promotes neovascularization and improves myocardial blood supply; scavenges free radicals and reduces oxidative stress
2022^[^ ^109^ ^]^	Polymers	Hyaluronic acid nanoparticles	All‐trans retinaldehyde, rapamycin	Passive targeting; Active targeting, hyaluronic acid	pH response	Atherosclerosis	Scavenging of ROS to reduce lipid peroxidation; inhibition of the MTOR pathway to reduce proliferation of macrophages and smooth muscle cells, and inhibition of the expression of inflammatory factors (e.g., TNF‐α and IL‐6)
2022^[^ ^85^ ^]^	Polymers	Zeolite imidazolate framework‐8 (ZIF‐8) nanoparticles	Losartan potassium	Passive targeting	pH response	Atherosclerosis	Induction of foam cell autophagy and activation of reverse cholesterol transport; inhibition of angiotensin II receptor and reduction of inflammatory factors (e.g., IL‐1β, IL‐6, and TNF‐α)
2022^[^ ^169^ ^]^	Polymers	Hyaluronic acid	Simvastatin	Active targeting, hyaluronic acid (HA) and CD44 receptor	pH response, enzyme response	Atherosclerosis	Inhibits HMG‐coa reductase and lowers cholesterol levels
2022^[^ ^174^ ^]^	Polymers	PLGA‐Pep‐PEG and PLGA‐PEG‐c	Rapamycin	Active targeting, c(rgdfc) peptide targeting integrin αvβ3	Histone K response	Atherosclerosis	Inhibition of phagocytosis and cytokine release by inflammatory macrophages and reduction of oxidized low‐density lipoprotein uptake
2020^[^ ^212^ ^]^	Polymers	Methyl‐beta‐cyclodextrin	Simvastatin	Passive targeting	Cholesterol response	Atherosclerosis	Inhibition of the inflammatory response and proliferation of macrophages and removal of cholesterol from plaques
2023^[^ ^172^ ^]^	Polymers	Polymer PMEMA	Glucocorticoids prednisolone and β‐cyclodextrin	Active targeting, CD44 receptor	ROS response, MMP‐9 response	Atherosclerosis	Inhibits macrophage M1 polarization and reduces lipid uptake; upregulates ABCA1/G1; enhances lipid efflux; solubilizes lipids
2021^[^ ^224^ ^]^	Polymers	PAMAM	Simvastatin	Passive targeting; Active targeting, erythrocyte membrane mimicry	ROS response; shear stress response	Atherosclerosis	Inhibits activation of inflammatory macrophages and reduces ROS production; inhibits cholesterol synthesis
2022^[^ ^73^ ^]^	Polymers	PLGA‐PEG‐PLGA nanoparticles	Perfluorohexane, Fe_3_O_4_, dir	Passive targeting; Active targeting, A‐type scavenger receptor	Low‐intensity focused ultrasound response	Atherosclerosis	ADV effect leads to macrophage apoptosis and reduces inflammatory response in plaques
2022^[^ ^81^ ^]^	Polymers	Fucoidan‐chitosan	Fucoidan	Active targeting, P‐selectin	ROS response, pH response	Atherosclerosis	Scavenging of DPPH radicals, superoxide and hydroxyl radicals to reduce oxidative stress; inhibition of LPS/IFN‐γ‐induced release of inflammatory factors (e.g., TNF‐α, IL‐6, IL‐1β) in RAW 264.7 macrophages
2020^[^ ^127^ ^]^	Polymers	Hyaluronic acid	Atorvastatin	Active targeting, CD44 receptor	Enzyme response, pH response	Atherosclerosis	Inhibition of the inflammatory response in macrophages and reduction of the expression of inflammatory factors (e.g., TNF‐α, IL‐1β, IL‐1α)
2023^[^ ^128^ ^]^	Polymers	Based on a π‐conjugated polymer (pmetpp‐MBT) as the core, dextran shells	Astaxanthin	Active targeting, VCAM‐1 receptor and CD44 receptor in macrophages; Mitochondrial targeting, SS‐31 peptide	pH response, ROS response	Atherosclerosis	Decreased pro‐inflammatory factors (TNF‐α, IL‐6) and increased anti‐inflammatory factors (IL‐10); up‐regulated ABCA1/G1 and down‐regulated CD36/LOX‐1 and decreased ox‐LDL uptake by macrophages
2016^[^ ^225^ ^]^	Polymers	PLA, PLGA, PEG	Interleukin 10	Passive targeting	–	Atherosclerosis	Activation of STAT3 and SOCS3 signaling pathways to inhibit inflammatory responses
2017^[^ ^89^ ^]^	Polymers	PLGA, lipid bilayer	Simvastatin	Active targeting, CD44 receptor, scavenger receptor BI	HAase response	Atherosclerosis	Promotion of intracellular cholesterol efflux and reduction of macrophage‐to‐foam cell conversion; inhibition of matrix metalloproteinase expression in macrophages
2022^[^ ^226^ ^]^	Polymers	Cyclodextrin, PMEMA	Prednisolone, lipid‐specific AIEgen	Active targeting, CD44 receptor	ROS response	Atherosclerosis	Inhibition of inflammatory responses in plaques; removal of lipids from plaques
2020^[^ ^74^ ^]^	Polymer micelles	PEG‐Ptyr‐EO	Simvastatin	Active targeting, CD44 receptor	ROS response	Atherosclerosis	Inhibits production of inflammatory factors (e.g., IL‐1β, IL‐6, and TNF‐α) and modulates the inflammatory immune microenvironment; reduces oxidative stress
2018^[^ ^63^ ^]^	Inorganic nanoparticles	Fe_3_O_4_, CeO_2_	CeO_2_	Passive targeting	ROS response	Atherosclerosis, rheumatoid arthritis	Scavenges ROS and reduces inflammatory response
2013^[^ ^227^ ^]^	Organic nanoparticles	Perfluorocarbon nanoparticles	–	Active targeting, VHPKQHR peptide	–	Atherosclerosis and breast cancer	VCAM‐1‐positive cells that specifically bind inflammatory endothelial cells and tumor vasculature
2018^[^ ^216^ ^]^	Inorganic nanoparticles	Copper sulfide nanoparticles	–	Passive targeting; Active targeting, TRPV1 channels	Light and heat response, temperature response	Atherosclerosis	Increased Ca^2^⁺ activates the AMPK signaling pathway, which in turn activates autophagy; reduced oxidized low‐density lipoprotein (oxldl)‐induced foam cell formation in vascular smooth muscle cells
2016^[^ ^228^ ^]^	Liposome	Phospholipids; Cholesterol	Short‐chain fatty acid acetates	Passive targeting	–	NAFLD	Reduces ATGL expression and serum FFA in vascular smooth muscle cells (SAT); downregulates hepatic lipid synthesis genes; increases hepatic mitochondrial OXPHOS complexes; induces SAT browning
2023^[^ ^87^ ^]^	Liposome	Disc‐shaped recombinant high‐density lipoprotein (rHDL), hyaluronic acid‐ferrocene conjugate (HA‐Fc)	Simvastatin	Active targeting, CD44 receptor, passive targeting	ROS response	Atherosclerosis	Inhibits secretion of inflammatory factors (e.g., IL‐6, TNF‐α, and CCL2) and attenuates plaque inflammation; promotes cholesterol efflux from macrophages/foam cells
2015^[^ ^108^ ^]^	Liposome	Hydrogenated Soy Phosphatidylcholine, cholesterol	Antisense microrna‐anti‐mir‐712	Active targeting, VHPK peptide	–	Atherosclerosis	Inhibition of mir‐712 expression restores the expression of its downstream genes TIMP3 (matrix metalloproteinase inhibitor‐3) and RECK, inhibits matrix metalloproteinase and deintegrin metalloproteinase activities, reduces extracellular matrix degradation, and prevents plaque formation
2017^[^ ^229^ ^]^	Liposome	Liposome	Liver X receptor agonist T0901317	Active targeting, intercellular adhesion molecule‐1	–	Atherosclerosis	Activation of hepatic X receptor signaling pathway inhibits PDGF‐BB‐induced proliferation of vsmcs
2023^[^ ^230^ ^]^	Liposome	Soybean phospholipids, cholesterol	Berberine	Active targeting, P‐selectin	–	Atherosclerosis, ulcerative colitis	Inhibits the expression of inflammatory factors (e.g., IL‐6, IL‐1β, and P‐selectin) and reduces the infiltration of inflammatory cells, exerting anti‐inflammatory effects
2021^[^ ^231^ ^]^	Liposome	Phospholipids, cholesterol	Dir (near infrared fluorescent dye)	Active targeting, CD36 receptor; passive targeting	–	Atherosclerosis	Blocking CD36‐mediated oxidized LDL uptake reduces lipid accumulation and inflammatory response; interfering with the binding pathway of oxldl to its receptor
2014^[^ ^213^ ^]^	Liposome	Recombinant high‐density lipoprotein nanoparticles	Simvastatin	Active targeting, scavenger receptor B1	–	Atherosclerosis	Inhibition of the mevalonate pathway reduces the production of inflammatory factors (e.g., TNF‐α and MCP‐1)
2021^[^ ^232^ ^]^	Liposome	Lipid nanoparticles (surface modified with natural apolipoprotein B‐100)	Rapamycin (loanword)	Active targeting, scavenger receptors (e.g., CD68)	–	Atherosclerosis	Rapamycin slows plaque progression by modulating the inflammatory response of macrophages and reducing inflammatory factors within the plaque
2019^[^ ^94^ ^]^	Liposome	Soybean phospholipids, cholesterol	Fluorescent dye Coumarin 6	Passive targeting	–	Atherosclerosis	In the plaque inflammatory microenvironment, neutrophils undergo netosis and release nets along with loaded drugs

### 6.3 Application in diabetes

The core mechanism of type 2 diabetes lies in the dual defect of insulin resistance and β‐cell dysfunction. In addition, downregulation of oxidative phosphorylation‐related gene expression and an abnormal fatty acid oxidation capacity led to the accumulation of lipid metabolic intermediates, which inhibit glucose cellular uptake.^[^ ^233^ ^]^

Based on insulin resistance and β‐cell damage caused by mitochondrial dysfunction in diabetes, nanomedicines can precisely target pancreatic β‐cells or skeletal muscle mitochondria to deliver antioxidants (e.g., SENDs and MitoPBN) or uncoupling agents (e.g., DNPME) to restore the function of the electron transport chain. Li et al. developed a mitochondrion‐targeting nanomedicine, T4O@TPP/PEG‐PLGA, using terpinene‐4‐ol (T4O) as a natural antioxidant and mitochondrial protector, and PEG‐PLGA as a nanocarrier. Treatment with T4O@TPP/PEG‐PLGA significantly reduced the ROS level in vascular smooth muscle cells, inhibited the expression of osteogenic markers (e.g., Runt‐related transcription factor 2 (RUNX2) and Bone morphogenetic protein 2 (BMP2))), and improved diabetic vascular calcification.^[^ ^150^ ^]^ Additionally, Huang et al. developed SENDs as a prodrug for an antioxidant enzyme, GPX1. SENDs displayed an exceptional ROS scavenging capacity, and 90% of ·OH radicals were cleared by SENDs at a concentration as low as 32 µg/mL. Such a high scavenging efficiency of SENDs is credited to their structure. The C‐Se bonds embedded in the carbon skeleton are controllably cleaved under ROS stimulation to gradually release selenium ions, not only avoiding the toxicity of free selenium ions but also precisely enhancing the expression of GPX1 in β cells.^[^ ^171^ ^]^

In addition to mitochondrial targeting, liver‐targeting nanomedicines offer broad‐spectrum intervention strategies for diabetes by regulating lipid metabolism and insulin sensitivity. Wu et al. encapsulated a liver‐targeting free radical scavenger, MitoPBN, within liposomes, resulting in negatively‐charged stable nanoparticles, Nano‐MitoPBN, with a diameter of ≈100 nm and a drug loading efficiency of 80.2%. Intraperitoneal injection of Nano‐MitoPBN to HFD mice at a dose of 2.5 mg kg^−1^ for a period of eight weeks resulted in a significant reduction in the fasting blood glucose concentration from 12 to 8 mmol L^−1^.^[^ ^234^ ^]^ Similarly, a platinum nanomedicine was prepared from biodegradable Pt‐SiO_2_ nanocapsules loaded with a mitochondrial uncoupling agent, DNPME. DNPME was released from the platinum nanomedicine to activate the AMPK pathway in a high ROS environment, reversing hepatic steatosis and insulin resistance.^[^ ^57^ ^]^ In addition, Zn^2^⁺ released from zinc oxide nanoparticles (ZnONPs) activated the AMPK/SIRT1 pathway, thereby inhibiting liver lipid synthesis genes (SREBP‐1c, FAS) and reducing fat production.^[^ ^101^ ^]^

**Figure 9:**
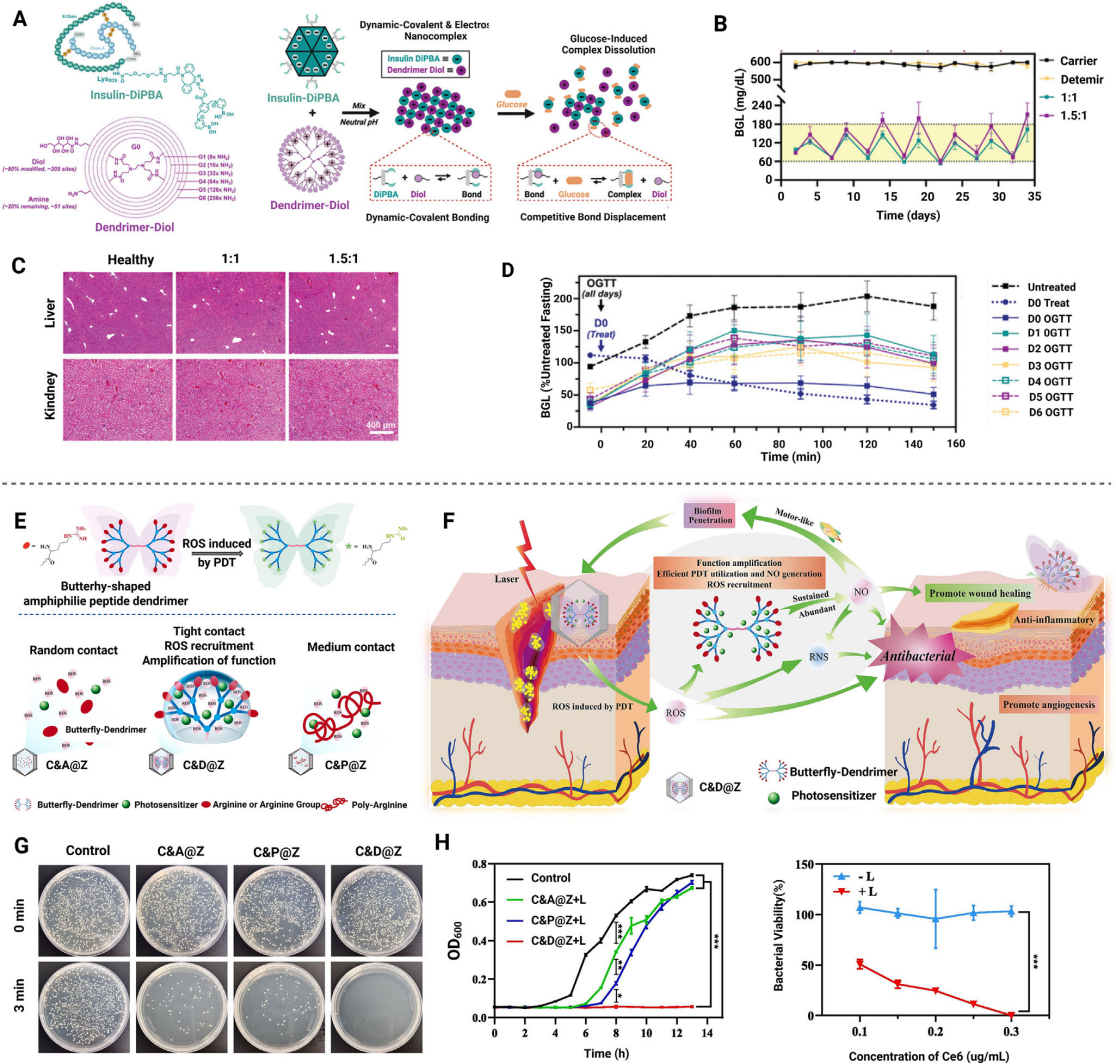
Engineering nanocarriers for targeted therapy of diabetes. A) Chemical structure of insulin‐DiPBA. When negatively charged insulin‐DiPBA was mixed with positively charged dendrimer‐diol, electrostatic interactions and dynamic covalent bonds between DiPBA and diol drove the formation of a nanocomplex. B) The blood glucose level in STZ‐induced diabetic mice administered the insulin‐dendrimer nanocomposite at a charge ratio of 1:1 or 1.5:1. C) Liver and kidney tissues in healthy mice after one month of continuous administration of the nanocomposite. D) The blood glucose level (BGL) in STZ‐induced diabetic mice administered with the insulin‐dendritic macromolecular nanocomposite after overnight fasting compared to that in the mice administrated with an equivalent dose of insulin detemir. Reproduced with permission.^[^ ^79^ ^]^ Copyright 2023, Wiley‐VCH GmbH. E) The basic principle of NO production from butterfly‐shaped amphiphilic peptide dendritic polymers and schematic diagrams of C&A@Z, C&P@Z, and C&D@Z. F) Schematic diagram of C&D@Z derived from butterfly‐shaped amphiphilic peptide dendritic polymers with antibacterial, anti‐inflammatory, and wound healing effects for the treatment of DFU infections. G) Representative LB agar plates of *staphylococcus aureus* colonies after treatment with C&A@Z, C&P@Z, and C&D@Z under different laser irradiation durations. H) Growth curves of *Staphylococcus aureus* (S. aureus) treated with different materials (C&A@Z + L, C&P@Z + L, and C&D@Z + L) over a 10‐h period. Semi‐quantitative results of *Staphylococcus aureus* colonies after treatment with different concentrations of C&D@Z. Reproduced with permission.^[^ ^78^ ^]^ Copyright 2025, Elsevier B.V. Created in https://BioRender.com.

In addition to the strategies for directly targeting mitochondria or metabolic organs to restore their functions, maintaining blood glucose homeostasis is crucial for alleviating mitochondrial oxidative stress in pancreatic β cells and insulin‐sensitive tissues (such as the liver and muscles). The development of intelligent, long‐lasting blood glucose control nanosystems can indirectly protect the mitochondrial function by reducing the glycemic load, which may be a promising direction for diabetes nanotherapy. For instance, Xian et al. developed a glucose‐responsive insulin‐dendrimer nanocomposite (**Figure** **9**A–D). This composite self‐assembled from insulin modified with pyridine diborate (DiPBA) and G6 PAMAM diol dendrimers via dynamic covalent bonds of borate esters and electrostatic interactions. By harnessing the unique glucose‐responsive property of DiPBA in this nanocomposite, the insulin release rate from the nanocomposite was significantly accelerated with an increase in the glucose concentration. A single subcutaneous injection of this nanocomposite was shown to maintain the normal blood glucose level in diabetic mice for a minimum of five days and effectively control the blood glucose concentration in a diabetic minipig model for a minimum of seven days. This long‐lasting medication in regulating the blood glucose level was achieved by continuously alleviating the glyco‐toxic stress on pancreatic β‐cells and the peripheral tissue mitochondria caused by a high‐glucose environment.^[^ ^79^ ^]^

In precision intervention studies for diabetes complications, different conditions exhibit targeted nanodrug therapy regimens. Unique regimens for targeted nanomedicines have been developed for specific diabetes complications to achieve precision intervention.^[^ ^235^ ^]^ For the treatment of diabetic foot ulcers, Xu et al. developed a novel drug delivery system, PBNPs@PLEL, by uniformly dispersing Prussian blue nanoparticles (PBNPs) in a thermosensitive poly(lactic acid)‐poly(ethylene glycol)‐poly(lactic acid) (PDLLA‐PEG‐PDLLA, PLEL) hydrogel. In vivo experiments demonstrated that PBNPs@PLEL promoted wound healing in diabetic mice by synergistically providing the ROS‐scavenging and antioxidant therapeutic action of PBNPs to alleviate oxidative stress, and the protective physical barrier, sustained release properties, and moist wound environment offered by the PLEL hydrogel matrix. On day 14 post‐treatment, a significant increase in the wound closure rate was found in the 100 µg PBNPs@PLEL group compared to the control group and the PLEL group.^[^ ^175^ ^]^ Huang et al. developed a multidrug co‐delivery nanosystem from arginine‐terminated butterfly‐shaped peptide dendrimers (C&D@Z) (Figure 9E–H). This system consisted of a zeolite imidazolate framework‐8 (ZIF‐8) backbone loaded with a photosensitizer, dihydroporphyrin e6 (Ce6), and arginine‐modified peptide dendrimers. Under NIR light irradiation, Ce6 was initially stimulated to generate ROS, which subsequently reacted with the butterfly‐shaped dendritic polymers to continuously produce a substantial quantity of nitric oxide (NO). In a murine model of methicillin‐resistant staphylococcus aureus infection induced by diabetes, the wound area was diminished to 7.88% within 10 days, a substantial improvement in comparison to the control group, which exhibited a wound area of 50.05%.^[^ ^78^ ^]^ In the study by Patricia et al., TPP‐AuCeO_2_ was prepared by depositing 0.1 wt% gold nanoparticles (5.3 ± 3.0 nm) onto the surface of CeO_2_ nanoparticles (5.2 ± 0.3 nm). In comparison with CeO_2_, TPP‐AuCeO_2_ demonstrated a substantial reduction in the intracellular ROS level, as evidenced by a 50% decrease in the DCFH‐DA fluorescence intensity. Furthermore, TPP‐AuCeO_2_ enhanced the inherent antioxidant defense mechanism in the body by stimulating NRF1/NFE2L1 protein expression, which was indicated by a 20‐30% increase observed through Western blot analysis.^[^ ^59^ ^]^

In summary, nanomedicine for diabetes primarily employs multi‐pathway synergistic interventions. These include the utilization of mitochondrial‐targeted strategies for the delivery of antioxidants, which protect pancreatic β‐cell function and improve insulin resistance. Furthermore, metabolic organs such as the liver are targeted in order to regulate lipid metabolism and insulin sensitivity. Additionally, glucose‐responsive smart nanosystems are developed for sustained glycemic control, which indirectly mitigates hyperglycemic toxicity to tissues. Finally, multifunctional nanomaterials with antioxidant, antibacterial, and wound‐healing properties are designed for the targeted treatment and management of complications like diabetic foot. A summary of representative nanomedicines used for diabetes treatment, including their types, materials, targeting mechanisms, and therapeutic actions (**Table** **3**).

**Table 3:** Different types of nanomedicines used for diabetes treatment.

Year	Types of nanomedicines	Nanomaterials	Drug loading	Target molecules	Targeting mechanisms	Stimuli‐response mechanisms	Diseases treated	Mechanisms of disease treatment
2023^[^ ^118^ ^]^	Polymers	Mesoporous Polydopamine Nanoparticles (MPDA)	SS31, a mitochondria‐targeting peptide	Mitochondrial inner membrane and localization of diabetic wounds	Active targeting, SS31 peptide	ROS response, pH response	Diabetic total skin defects (diabetic foot ulcers)	Reduces oxidative stress levels; promotes M2 macrophage polarization (CD206, IL‐4/IL‐13); upregulates PI3K/Akt/mTOR pathway
2024^[^ ^150^ ^]^	Polymers	PEG‐PLGA	Terpinen‐4‐ol	Mitochondria	Mitochondrial targeting, TPP modification	–	Diabetic vascular calcification	Reduces mitochondrial ROS levels, restores SOD activity, and reduces lipid peroxidation products (MDA); inhibits expression of mitochondrial splitting protein DRP1 and promotes expression of fusion protein MFN1
2012^[^ ^76^ ^]^	Dendrimers	Polyamidoamine‐G3	–	–	Passive targeting	–	Diabetic cardiomyopathy	Amino capture of methylglyoxal, reduces protein glycosylation and maintains complex II (succinate dehydrogenase) activity
2010^[^ ^77^ ^]^	Dendrimers	Polyamidoamine‐G4	–	–	Passive targeting	–	Diabetic cardiomyopathy	Reduced levels of fat‐soluble antioxidants (e.g., alpha‐tocopherol, coenzyme Q) in myocardial and hepatic tissues to alleviate oxidative stress
2025^[^ ^78^ ^]^	Dendrimers	Butterfly‐shaped peptide dendritic polymer, ZIF‐8	Chlorin e6	–	Passive targeting	Light response, pH response	Sites of infection in diabetic foot ulcers	Anti‐inflammatory and promotes angiogenesis
2024^[^ ^79^ ^]^	Dendrimers	PAMAM dendrimer with peripherally modified diols	Insulin‐dipba	–	–	Glucose concentration response	Diabetes	Promotes glucose uptake, storage and utilization
2023^[^ ^172^ ^]^	Inorganic Nanoparticles	Sends	Se	Liver and pancreatic beta cells	Passive targeting	ROS response	Type 2 Diabetes Mellitus (T2DM)	Scavenging ROS, inhibiting lipid peroxidation and oxidative DNA damage; restoring GPX1 activity; restores mitochondrial membrane potential; inhibits PERK/eif2α/ATF4/CHOP pathway
2023^[^ ^57^ ^]^	Inorganic Nanoparticles	Pt‐SiO_2_	2,4‐Dinitrophenol methyl ether	Liver, mitochondria	Passive targeting; mitochondrial targeting, mitochondrial membrane potential dependent	ROS response	T2DM	Scavenging of ROS; inhibition of mitochondrial ETC; activation of the restoration of the hepatocyte insulin signaling pathway (IRS‐1/PI3K/Akt)
2023^[^ ^62^ ^]^	Inorganic Nanoparticles	AgNPs	AgNPs	–	Passive targeting	–	Diabetes mellitus complications	ROS scavenging; restoration of respiratory chain complex I, II and II+III activity; Antioxidant enzyme activation; reduction of thiobarbituric acid reactive substances (TBARS) levels in brain mitochondria
2022^[^ ^86^ ^]^	Inorganic Nanoparticles	Single‐walled carbon nanotubes	Cyt C	Mitochondria	Active targeting, vesicle protein‐mediated endocytosis	–	Cancer, T2DM, neurodegenerative diseases, myocardial ischemia, etc.	ROS scavenging; mitochondrial membrane potential restoration; reduction of protein carbonylation and lipid peroxidation
2022^[^ ^176^ ^]^	Inorganic Nanoparticles	Prussian blue nanoparticles, poly (levulinic acid‐ethylene glycol‐levulinic acid) (PDLLA‐PEG‐PDLLA) triblock copolymers	Pb NPs	–	Passive targeting	Temperature response	Diabetic foot ulcer	ROS scavenging; restores mitochondrial membrane potential and reduces calcium overload; Inhibition of pro‐inflammatory factors (IL‐6, TNF‐α)
2020^[^ ^59^ ^]^	Inorganic Nanoparticles	TPP‐AuCeO_2_	CeO_2_, Au NPs	Mitochondria	Active targeting, TPP modification	–	Diabetes, cancer, AD, obesity and stroke	Neutralizes ROS; increases CAT‐like activity; restores mitochondrial membrane potential; upregulates NRF1 and NFE2L1 gene and protein expression
2024^[^ ^81^ ^]^	Polymers	MPDA	Resveratrol	Mitochondria	Mitochondrial targeting	Light response, ROS response	Chronic diabetic wounds	Scavenges ROS and restores mitochondrial membrane potential; promotes macrophage M2 polarization; inhibits inflammation (IL‐6) and activates AMPK/SIRT1/PGC‐1α pathway
2024^[^ ^236^ ^]^	Inorganic Nanoparticles	CuO NPs	The surface of CuO NPs was coated with clove (Syzygium aromaticum) bud extract and α‐tocopherol (vitamin E)	Liver disease	Passive targeting	–	Diabetes‐related complications	Regulates lipid metabolism; reduces oxidative stress
2016^[^ ^182^ ^]^	Inorganic Nanoparticles	Calcium phosphate nanoparticles	Insulin	Intestine	Active targeting, Intrinsic factor receptor	pH response	Diabetes	Promotes insulin absorption
2021^[^ ^237^ ^]^	Hybrid Nanoparticles	Chitosan ‐sodium alginate‐oleic acid composite nanoparticles	Lutein	–	Passive targeting	–	Diabetic retinopathy	Reduced ROS; inhibition of mitochondrial membrane potential collapse
2024^[^ ^238^ ^]^	Hybrid Nanoparticles	Polymer complexes of filipin protein and polycaprolactone	Berberine	–	–	–	Diabetic alveolar bone defects	Up‐regulation of LC3‐II/I reduces P62 accumulation, alleviates autophagic flow blockage, and reduces apoptosis; Runx2/Alp gene expression is significantly elevated
2024^[^ ^80^ ^]^	Hybrid Nanoparticles	Chitosan lipid nanoparticles	Piperine	–	Passive targeting	–	Diabetes‐related cognitive deficits (e.g., diabetic encephalopathy, neuroinflammation)	Anti‐oxidative stress; upregulation of BDNF and CREB expression in the hippocampus
2024^[^ ^117^ ^]^	Hybrid Nanoparticles	Lipoic acid‐functionalized platycodin D nanocarriers	Platycodin D	Mitochondria	Mitochondrial targeting, TPP modification	–	T2DM	Reduces oxidative stress; regulates GSK3‐β/IRS1/AMPK signaling pathway
2015^[^ ^170^ ^]^	Hybrid Nanoparticles	Alginate/Trimethyl Chitosan (TMC) Nanoparticles	Insulin	Intestine	Passive targeting	pH response	Diabetes	Insulin to lower blood sugar
2012^[^ ^133^ ^]^	Hybrid Nanoparticles	Alginate/Chitosan Coated Nanoemulsion	Insulin	Intestine	Passive targeting	pH response	Diabetes	Insulin to lower blood sugar
2019^[^ ^234^ ^]^	Liposome	Soybean phospholipids, cholesterol	MitoPBN	Liver, mitochondria	Passive targeting; active targeting, TPP modification	–	T2DM	ROS clearance; enhanced insulin sensitivity (upregulation of AKT phosphorylation), decreased inflammatory factors (IL‐6, TNF‐α)
2021^[^ ^194^ ^]^	Liposome	Soybean phospholipids, cholesterol	MitoPBN	Liver, mitochondria	Passive targeting	–	T2DM	Reduces oxidative stress; activates AMPK/SIRT3/PGC‐1α signaling pathway

### 6.4 Application in Non‐Alcoholic Fatty Liver Diseases

In NAFLD, adaptive responses of mitochondria to lipid overload result in compensatory enhancement in the oxidative capacity and a decrease in the energy coupling efficiency, leading to lipid deposition and insulin resistance.^[^ ^201^, ^205^ ^]^ Typical features of mitochondrial dysfunction in NAFLD include, ROS bursts, depletion of antioxidant defense systems (such as SOD2 and GPX), mitochondrial DNA damage, and increased proton leakage.^[^ ^239^, ^240^, ^241^, ^242^ ^]^

To treat NAFLD, nanocarriers for small molecular drugs can be customized by following the “targeted + responsive + synergistic” principles to ensure drug accumulation and efficient release at the lesion site. These nanocarriers can be modified with ligands (e.g., Gal, LA, or TPP) or stimulus‐responsive moieties by harnessing pathological microenvironmental characteristics (e.g., elevated GSH and ROS levels) to achieve dual‐targeted delivery to hepatocytes and mitochondria, thereby enhancing therapeutic efficacy while reducing off‐target toxicity. For instance, Res has been shown to exert antioxidant, anti‐inflammatory, and lipid metabolism‐regulating effects. However, its low water solubility and poor bioavailability has hampered its clinical application. To address this issue, Teng et al. developed a Gal‐functionalized oxidized starch‐lysozyme (Gal‐OSL) nanocarrier loaded with Res as a liver‐targeting nanomedicine, Gal‐OSL/Res. In vivo experiments demonstrated that after 6 weeks of treatment, triglyceride (TG) and (MDA levels in mouse livers decreased by 55% and 40%, respectively.^[^ ^102^ ^]^

In another study, a glycogen (Gly)‐based liver‐targeting, redox‐responsive nanomedicine (Gly‐LA‐LacNPs) was developed by modifying α‐LA and Lac to achieve efficient loading and delivery of Res. The disulfide bonds in α‐LA were reduced in a high‐concentration glutathione (GSH) environment, leading to the degradation of the nanodrug and the release of Res. After a period of six weeks post treatment of mice with this nanodrug, serum levels of ALT, AST, TC, and TG in the HFD mice decreased by 45%, 38%, 32%, and 41%, respectively, which were accompanied by a substantial reduction in hepatic lipid deposition.^[^ ^107^, ^244^ ^]^

It is noteworthy that the delivery bottleneck of resveratrol can be applied to other natural bioactive compounds which face the same bioavailability challenge. A growing body of research has identified dual or multi‐targeted strategies for these natural drugs, offering novel approaches to enhancing their efficacy and safety. Astaxanthin (AXT) has been shown to protect the liver cell mitochondrial function by scavenging ROS. However, its lipophilic nature and gastrointestinal instability has diminished its efficacy. Wan et al. designed a dual‐targeted nanomedicine by introducing TPP into the surface of the nanomedicine through an esterification reaction with hydroxypropyl‐β‐cyclodextrin (HD). It was found that AXT was successfully delivered to hepatic mitochondria. In an oxidative stress model, the ROS level in the dual‐functional nanomedicine group was significantly reduced to 62.20%. The recovery rate of the mitochondrial membrane potential was 97.35%, which was significantly higher than that in the single‐targeted group.^[^ ^245^ ^]^

In addition to complex active targeting strategies, physicochemical properties of nanocarriers (e.g., size and charge) can be tuned to achieve passive targeting and prolonged retention. Silymarin (SIL), a well‐known hepatoprotective agent, has been shown to increase it bioavailability through the use of a chitosan nanocarrier.^[^ ^246^ ^]^ In this study, electrostatic interaction between positively charged chitosan and negatively charged sodium tripolyphosphate resulted in the formation of ionically cross‐linked nanoparticles which were loaded with silymarin (SILNPs). These nanoparticles, with an approximate diameter of 100 nm, were shown to extend the duration of drug retention in liver tissues through mechanisms of passive targeting, such as the EPR effect. In a model of liver injury induced by CCl4 in rats, the high‐dose SILNP group, administered with a dosage of 50 mg kg^−1^ of body weight, displayed a 67% reduction in the serum ALT level and a 63% reduction in the serum AST level, both of which approached the normal levels.^[^ ^122^ ^]^ Curcumin has been demonstrated its potential in the treatment of metabolic liver diseases.^[^ ^247^ ^]^ Choudhury et al. compared two physical encapsulation methods for curcumin, one encapsulated within liposomes and the other in PLGA nanoparticles. Physicochemical properties of two curcumin nanomedicines revealed that the PLGA nanomedicine (35 ± 9 nm) had a smaller particle size and a narrower size distribution than the liposome nanomedicine (140 ± 60 nm). A small size of the PLGA nanomedicine facilitated its passive targeting through the liver sinusoids endothelium and enhanced its intracellular retention. In a mouse model, the PLGA nanomedicine (35 ± 9 nm) administered orally exhibited an AUC_0_₋∞ of 2025.59 µg/mL/min that was 3.13 times higher than that of the liposome nanomedicine. This delivery strategy may be particularly effective for lipophilic drugs for the treatment of metabolic liver diseases.^[^ ^248^ ^]^

In contrast to antioxidant strategies, multidimensional intervention can be attained by means of synergistic regulation of lipid metabolism and inflammatory responses. For instance, Wang et al. employed an oxidative method to modify kelp cellulose, resulting in a surface that was abundant in carboxyl groups. This surface exhibited high dispersibility, a substantial negative charge with a zeta potential of ≈−29.34 mV, and a porous network structure. Kelp nanocellulose was assembled with sodium caseinate (SC) to form a composite nanodrug (TKNC/SC) with an approximate particle size of 285 nm. The nanodrug treatment resulted in a 1.87‐fold increase in Nrf2 protein expression, as well as 1.71‐fold and 2.01‐fold increases in the HO‐1 and NQO1 mRNA levels, respectively.^[^ ^249^ ^]^

We have demonstrated the use of nanomedicines to enhance the delivery efficiency of a single drug for treating NAFLD. However, the intricate pathology of NAFLD marked by disrupted lipid metabolism and oxidative stress requires multi‐targeted, synergistic interventions.^[^ ^250^ ^]^ Smart nanoplatforms capable of delivering or generating multiple active components in a simultaneous manner could be developed for synergistic interventions. Fenofibrate (FNB), a PPARα agonist, promotes fatty acid oxidation; however, its systemic side effects have hampered its application. In a seminal study, Zhou et al. utilized vitamin E‐derived peroxides (OVE) as carriers to successfully load FNB. In a high‐ROS microenvironment associated with NAFLD, the OVE nanocarrier was observed to release FNB and generate α‐tocopherol. This dual “therapeutic and protective” function from FNB and α‐tocopherol regulated lipid metabolism and oxidative stress in a simultaneous manner. In comparison with free drugs, the FNB concentration in the liver tissue reached 2.3 times that of free drugs.^[^ ^251^ ^]^

**Figure 10:**
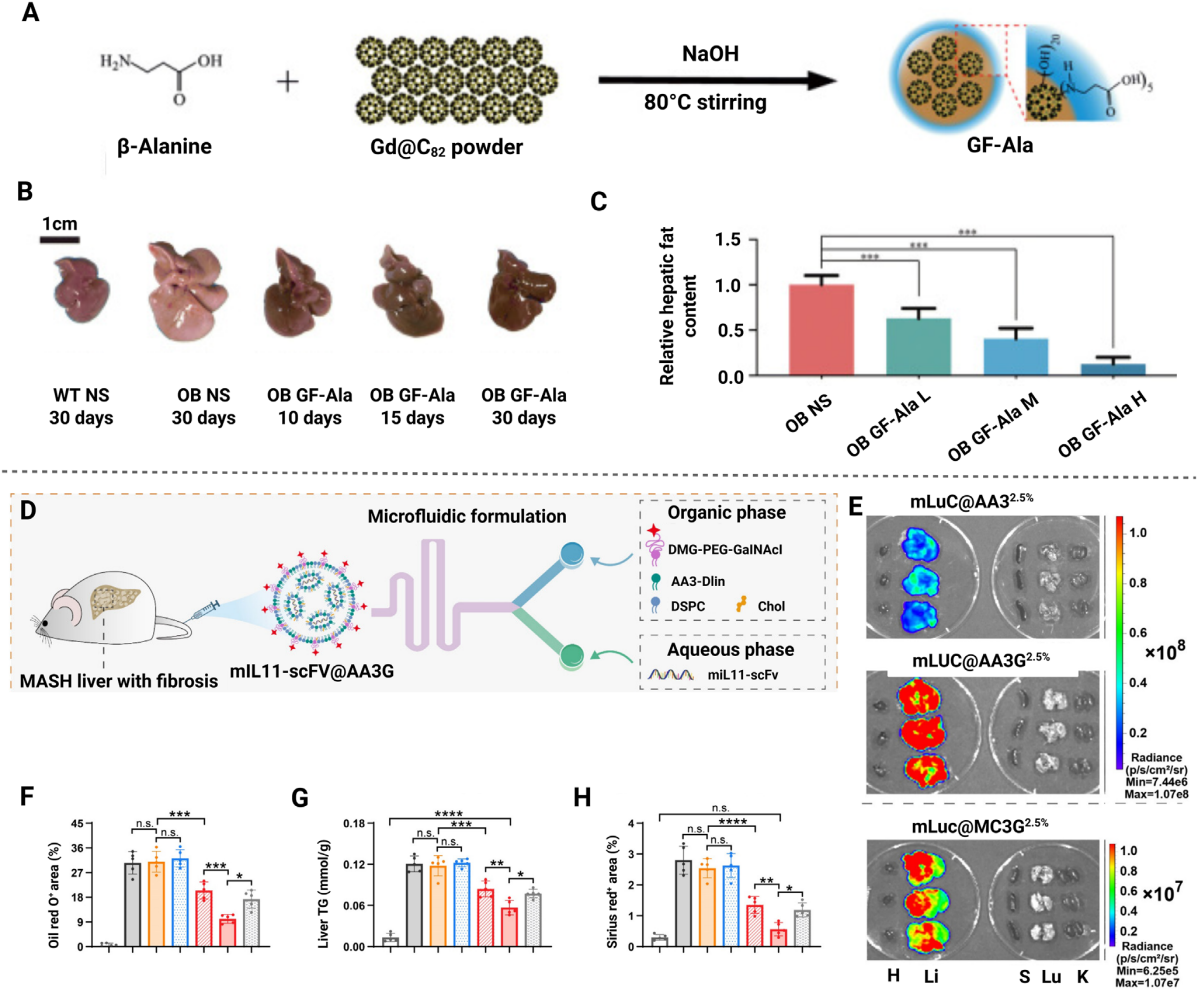
Engineering nanocarriers for precise intervention in liver diseases. A) Schematic diagram of synthesis of GF‐Ala, a β‐alanine‐functionalized gadolinium fullerene to inhibit ApoB100 degradation by scavenging ROS, promote triglyceride transport, and efficiently reverse hepatic steatosis and obesity‐related metabolic disorders. B) Photographs of liver tissues after euthanasia following GF‐Ala treatment at different treatment periods. C) The liver fat content after GF‐Ala treatment. Reproduced with permission.^[^ ^243^ ^]^ Copyright 2020, American Association for the Advancement of Science. D) Schematic diagram of mIL11‐scFv‐mediated liver‐specific antibody therapy using AA3G‐targeted LNP for the treatment of MASH and liver fibrosis. E) In vitro luminescence images in the heart (H), liver (Li), spleen (S), lung (Lu), and kidney (K) of healthy mice at 6 h after treatment with a 2.5% LNP formulation. F) Quantitative analysis of Oil Red O‐positive areas in liver tissue sections. G) Liver triglyceride (TG) content in liver tissue sections. H) Quantitative analysis of Siri red‐positive areas in liver tissue sections. Reproduced with permission.^[^ ^106^ ^]^ Copyright 2021, Springer Nature Limited. Created in https://BioRender.com.

In addition to small‐molecule drugs, certain nanomaterials with intrinsic biological activity can serve as a therapeutic agent, offering a new avenue for NAFLD intervention. An example of the nanomaterials is the gadolinium fullerene nanodrug (GF‐Ala) (**Figure** **10**A–C). This compound inhibited the degradation of apolipoprotein B100 (ApoB100) by scavenging ROS, thereby reversing hepatic steatosis.^[^ ^243^ ^]^ Apart from small‐molecule drugs and bioactive nanomaterials, nucleic acid drugs have been explored to transiently regulate endogenous repair pathways in the liver. Recent studies have demonstrated that LNP‐mediated delivery of nucleoside‐modified mRNA effectively and specifically induced transient expression of HGF and EGF in hepatocytes that lasted approximately three days.^[^ ^69^, ^106^ ^]^ This non‐integrating mRNA‐LNP platform presents a novel, highly translational therapeutic strategy for promoting liver repair and reversing metabolic liver injury by precisely and controllably activating endogenous regenerative pathways (Figure 10D–H).

Although the nanomedicines mentioned above have shown promise in the treatment of NAFLD, their long‐term safety and clinical translation challenges remain to be addressed. For example, metal nanomedicines (such as cerium oxide) can clear ROS through the Ce^3^⁺/Ce⁴⁺ cycle, but their prolonged retention in the body may be detrimental; meanwhile, the degradation products of polymer carriers (e.g., PLGA) should be evaluated for their biocompatibility. Targeting and controlled‐release could be integrated through multifunctional nanoplatforms to advance the development of precision therapy for NAFLD.^[^ ^121^ ^]^

In summary, nanomedicine for treating NAFLD primarily relies on the synergistic effects of multiple strategies. These include the utilisation of liver‐targeting ligands or the modulation of the physical properties of nanoparticles to achieve drug enrichment in the liver. In addition, the design of smart carriers responsive to elevated hepatic reactive oxygen species or glutathione for precise drug release is also employed. Furthermore, the simultaneous modulation of lipid metabolism and oxidative stress by encapsulating natural compounds such as resveratrol or the development of nanomaterials with dual therapeutic and antioxidant functions is a key aspect of the field. Finally, the advancement of nanoplatforms capable of co‐delivering multiple drugs or nucleic acids for multi‐targeted synergistic intervention against fatty liver disease is of great significance. A summary of representative nanomedicines used for NAFLD treatment, including their types, materials, targeting mechanisms, and therapeutic actions (**Table** **4**).

**Table 4:** Different types of nanomedicines used for NAFLD treatment.

Year	Types of nanomedicines	Nanomaterials	Drug loading	Target molecules	Targeting mechanisms	Stimuli‐response mechanisms	Diseases treated	Mechanisms of disease treatment
2024^[^ ^91^ ^]^	Biomimetic nanoparticles	Macrophage membrane shells, diselenide‐bridged mesoporous silica cores	All‐trans retinoic acid, labetalol	Activated hepatic stellate cells	Active targeting, CD44 receptor	ROS response	NAFLD	Inhibit the activation of aHSCs and lipid accumulation induced by sympathetic neurotransmitters; promote the return of aHSCs from activation to quiescence. Reduces intrahepatic TG and TC levels.
2019^[^ ^102^ ^]^	Micelle	Galactose‐modified oxidized starch‐lysozyme nanocarriers	Resveratrol	Liver disease	Active targeting, ASGPR	–	NAFLD	AMPK/SIRT1/FAS/SREBP1c pathway activation; inhibition of phosphorylation at the Ser307 site of IRS‐1 reduces glucose and insulin tolerance
2023^[^ ^251^ ^]^	Polymers	Vitamin E‐derived peroxylates, DSPE‐PEG as amphiphilic conjugates	Fenofibrate	Liver disease	Passive targeting	ROS response	NAFLD	Activation of PPARα; attenuation of oxidative stress; reduction of lipid peroxidation and restoration of mitochondrial function
2022^[^ ^107^ ^]^	Polymers	Glycogen‐based nanoparticles	Resveratrol	Liver disease	Passive targeting, hepatophilicity of Gly‐NPs; active targeting, ASGPR	GSH response	NAFLD	Reduces oxidative stress; regulates TLR4/NF‐κB signaling pathway and inhibits expression of inflammatory factors (e.g., TNF‐α, IL‐1β, IL‐6)
2024^[^ ^252^ ^]^	Polymers	Polydopamine Nanoparticles, PDNPs	PDNPs	Liver disease	Passive targeting	–	NAFLD	PDNPs scavenge ROS via their surface phenolic moieties; reduce lipid accumulation; reduce lipid synthesis
2019^[^ ^253^ ^]^	Polymers	Chitosan	Interleukin‐22 (IL‐22) gene	Liver disease	Passive targeting	–	NAFLD	Activation of STAT3/Erk1/2 signaling pathway in hepatocytes; activation of Nrf2/SOD1 pathway; up‐regulation of fatty acid β‐oxidation genes (Acox1, Cpt1a) and lipid transporter genes (Acc1), and inhibition of fatty acid synthesis genes (FAS)
2020^[^ ^181^ ^]^	Polymers	Meo‐PEG‐b‐PDPA	Steatohepatitis‐associated circRNA ATP5B Regulator	Liver, mitochondria	Mitochondrial targeting, TPP modification	pH response	NASH	circRNA SCAR inhibits NASH‐associated inflammation and fibrosis by binding to ATP5B and blocking mPTP opening
2020^[^ ^122^ ^]^	Polymer nanoparticles	Chitosan	Silymarin	Liver disease	Passive targeting	–	Liver cell necrosis, fibrosis	Down‐regulation of pro‐apoptotic genes (Bax, p53, Caspase‐3) and up‐regulation of anti‐apoptotic genes (Bcl‐2)
2020^[^ ^243^ ^]^	Inorganic nanoparticles	Gadofullerene nanoparticles	Triglycerides	Liver disease	Passive targeting	ROS response	Hepatic steatosis	Inhibits the degradation of ApoB100 and facilitates the transport of TG from the liver to the bloodstream
2017^[^ ^121^ ^]^	Inorganic nanoparticles	Nanocrystalline cerium dioxide	CeO_2_	Liver, mitochondria	Passive targeting	–	NAFLD	Reduces pro‐inflammatory factors (IL‐1β, IL‐12Bp40) and restores anti‐inflammatory factors (IL‐4, IL‐10, TGF‐β) to normal levels; activates AMPK‐PPARα signaling pathway
2016^[^ ^196^ ^]^	Inorganic nanoparticles	CeO_2_	CeO_2_	Liver, spleen	Passive targeting	–	Liver fibrosis	Inhibition of gene expression of NADPH oxidases (Ncf1, Ncf2), alleviation of oxidative stress and ER stress pathway (Atf3, Hspa5 downregulation)
2019^[^ ^101^ ^]^	Inorganic nanoparticles	ZnO	ZnO	Liver disease	Passive targeting	–	NAFLD	AMPK/SIRT1 signaling pathway activation
2024^[^ ^254^ ^]^	Organic nanoparticles	Obeticholic acid and atorvastatin	Obeticholic acid, atorvastatin	Liver disease	Passive targeting	–	NAFLD	Activates FXR receptors and inhibits lipid synthesis genes; Inhibition of HMG‐CoA reductase
2024^[^ ^255^ ^]^	Hybrid nanoparticles	Whey protein isolate, WPI, galactose oligosaccharide	Astaxanthin	Liver, mitochondria	Active targeting, ASGPR; mitochondrial targeting, TPP modification	pH response	NAFLD	Reduces oxidative stress; maintains mitochondrial membrane potential and reduces mitochondrial dysfunction and apoptosis
2023^[^ ^245^ ^]^	Hybrid nanoparticles	Sodium alginate, hydroxypropyl‐β‐cyclodextrin	Astaxanthin	Liver, mitochondria	Active targeting, ASGPR; mitochondrial targeting, TPP modification	pH response	NAFLD	Reduces oxidative stress; maintains mitochondrial membrane potential and reduces mitochondrial dysfunction and apoptosis
2025^[^ ^249^ ^]^	Hybrid nanoparticles	Kelp Nanocellulose, TKNC, Sodium Caseinate	Fucoxanthin	Liver disease	Passive targeting	–	NAFLD, obesity	Activation of the Nrf2/HO‐1/NQO1 signaling pathway
2011^[^ ^256^ ^]^	Hybrid nanoparticles	Cyclodextrin Complex	Naringenin	Liver disease	Passive targeting	–	Hyperlipidemia, diabetes, HCV infection	Activation of Nrf2 signaling pathway; activation of PPARα and PPARγ pathways
2016^[^ ^248^ ^]^	Hybrid nanoparticles	Phosphatidylethanolamine (PE), cholesterol, di‐cetylphytosphingosine phosphate (DCP), PLGA	Curcumin	Liver disease	Passive targeting	–	Hepatocellular necrosis and steatosis	Increases intracellular antioxidant enzyme activity; reduces mitochondrial membrane permeability changes; block PARP (poly ADP ribose polymerase) cleavage to avoid DNA damage
2021^[^ ^69^ ^]^	Lipid nanoparticles	Ionizable cationic lipids, phosphatidylcholine, cholesterol	Nucleoside‐modified mRNA (encoding hepatocyte growth factor HGF and epidermal growth factor EGF)	Liver disease	–	–	NAFLD	Induced DNA synthesis in hepatocytes and significantly increased the number of EdU+ hepatocytes
2022^[^ ^70^ ^]^	Lipid nanoparticles	Electrolyzable lipids, phospholipids, cholesterol	HMGB1‐siRNA	Liver macrophage	Active targeting, macrophage surface mannose receptor	–	NASH	Silencing of macrophage HMGB1 gene, decreasing serum levels of inflammatory factors such as IL‐6 and TNF‐α; promotes the conversion of pro‐inflammatory M1‐type macrophages to anti‐inflammatory M2‐type macrophages
2020^[^ ^105^ ^]^	Liposome	Soy Lecithin, cholesterol	Baicalin	Liver disease	Passive targeting	–	NAFLD	Inhibition of the Toll‐like receptor 4 (TLR4) signaling pathway to reduce the production of inflammatory mediators (e.g., TNF‐α, IL‐1β, IL‐6, etc.)
2018^[^ ^120^ ^]^	Liposome	Egg yolk phosphatidylcholine, egg yolk phosphatidylglycerol, cholesterol	Curcumin, 1,25‐Dihydroxyvitamin D3/Calcitriol	F4/80⁺, inflammatory DCs and macrophages, liver	Passive targeting	–	NASH	Inhibition of the JNK‐MAPK‐NF‐κB pathway, reduction of pro‐inflammatory factors such as TNF‐α and IL‐6, and up‐regulation of IL‐10, IL‐4 and oxidative phosphorylation‐related genes (PPARγ, MRC‐1)
2024^[^ ^106^ ^]^	Liposome	Electrolyzable lipids, cholesterol	mRNA encoding a single‐stranded variable region fragment (scFv) of IL‐11 (mIL11‐scFv)	Liver disease	Active targeting, ASGPR	–	MASH	Inhibits JNK/ERK phosphorylation and reduces pro‐inflammatory factor (TNF‐α, CCL2, CCL5) expression and inflammatory cell infiltration

### 6.5 Applications in Brain Metabolic Disorders

Recent studies have revealed that neurodegenerative diseases such as AD and PD, though traditionally not classified as metabolic disorders, exhibit numerous characteristic metabolic abnormalities. These phenomena are now recognized as manifestations of brain metabolic disorders.^[^ ^257^ ^]^ These diseases exhibit analogous pathophysiological characteristics to peripheral metabolic syndrome, including insulin resistance, glucose metabolism disorders, and chronic oxidative stress. However, their primary lesions are localized to the CNS, extending beyond the BBB.^[^ ^258^ ^]^ A substantial body of research has identified mitochondrial dysfunction as a prevalent pathological nexus in neurodegenerative diseases such as AD and PD. Impaired mitochondrial energy metabolism and excessive ROS production have been demonstrated to directly result in neuronal damage. This metabolic abnormality has led some scholars to label AD as “Type 3 diabetes”, emphasizing its connection to systemic metabolic disorders like brain insulin resistance and systemic T2DM.^[^ ^257^ ^]^ In addition, other neurodegenerative diseases, including Huntington's disease and amyotrophic lateral sclerosis, have been shown to exhibit significant mitochondrial dysfunction and metabolic defects. This finding underscores the broad significance of cerebral metabolic dysregulation in neurodegenerative pathology. Collectively, brain metabolic dysfunction occupies a pivotal intersection between neurodegenerative pathology and systemic metabolic disorders, with mitochondria emerging as a core therapeutic target.

Mitochondria‐targeted nanomedicines have emerged as a promising avenue for addressing the complex pathology of AD, evolving from simple antioxidant nanocarriers to multifunctional brain‐targeting platforms. One of the earliest examples is the mitochondria‐targeted nanocatalyst, TPP‐CeO_2_. The Hyeon team demonstrated that these nanoparticles localize to neuronal mitochondria to scavenge excess reactive oxygen species, thereby suppressing neurotoxic oxidative stress. In an AD mouse model, a single intracerebral administration was found to preserve ≈40% of hippocampal neurons that would have otherwise been lost to degeneration,^[^ ^259^ ^]^ thereby demonstrating the technology's potential for combating mitochondrial oxidative damage in vivo. Building on this concept, researchers have designed more sophisticated nanomedicines that not only neutralize ROS but also modulate protein aggregation and neuroinflammation. For instance, Ren et al. developed dual‐functional molybdenum disulfide quantum dots (TPP‐MoS_2_ QDs) with mitochondrial‐targeting TPP modification. These ultramicroscopic nanozymes exhibit dual enzyme‐mimetic activity, including superoxide dismutase and catalase, which effectively reduce intracellular superoxide and hydrogen peroxide levels. It is noteworthy that TPP‐MoS_2_ QDs have been observed to penetrate the BBB and accumulate in the brain. This phenomenon has been shown to significantly mitigate the toxic effects of Aβ oligomers by “repolarizing” microglia from pro‐inflammatory M1 to anti‐inflammatory M2 phenotypes. Furthermore, TPP‐MoS_2_ QDs demonstrated a notable efficacy in mitigating Aβ‐induced oxidative stress, exhibiting a reduction in ROS levels of ≈55.4% and 79.2%, respectively, compared to the Aβ group. The intensity of mitochondrial autophagy markers (red fluorescence) decreased by ≈60.4% in the TPP‐treated group compared to the Aβ group, indicating significantly reduced mitochondrial damage and autophagy.^[^ ^151^ ^]^

Recent studies in AD research further underscore the potential of mitochondria‐targeted nanomedicines. Han et al. designed a dual‐modified biomimetic nanomaterial system, RVG/TPP‐RSV NPs@RBCm, featuring a lipid nanocarrier core encapsulated by red blood cell membrane. The membrane surface incorporates rabies virus glycoprotein peptide RVG29 and TPP groups, enabling a “cross‐BBB + mitochondrial targeting” cascade positioning. The system was found to be loaded with resveratrol (RSV), which led to a substantial reduction in mitochondrial and intracellular ROS levels (by ≈60% or more) in HT22 neurons in vitro. Additionally, it was observed that the system was capable of restoring superoxide dismutase (MnSOD) expression and decreasing lipid peroxidation end product MDA levels. In APP/PS1 mice, the intravenous administration of RVG/TPP‐RSV NPs@RBCm effectively ameliorated memory deficits. The Morris water maze test revealed a reduction in escape latency and an increase in platform crossings, indicating a significant cognitive recovery compared to the control group.^[^ ^114^ ^]^ Another study by Qian et al. reported the development of the cholinergic neuron‐targeted nanosystem FGL‐NP(Cit)/HNSS. This nanosystem displays acid‐sensitive charge reversal properties, expeditiously releasing HNSS within lysosomes and partially directing it to mitochondria via SS31, thereby restoring mitochondrial ultrastructure and homeostasis. In 3xTg‐AD transgenic mice, treatment led to a significant reduction in Aβ deposition and hyperphosphorylated tau levels, while concurrently improving memory deficits and cholinergic neuronal damage.^[^ ^115^ ^]^

**Figure 11:**
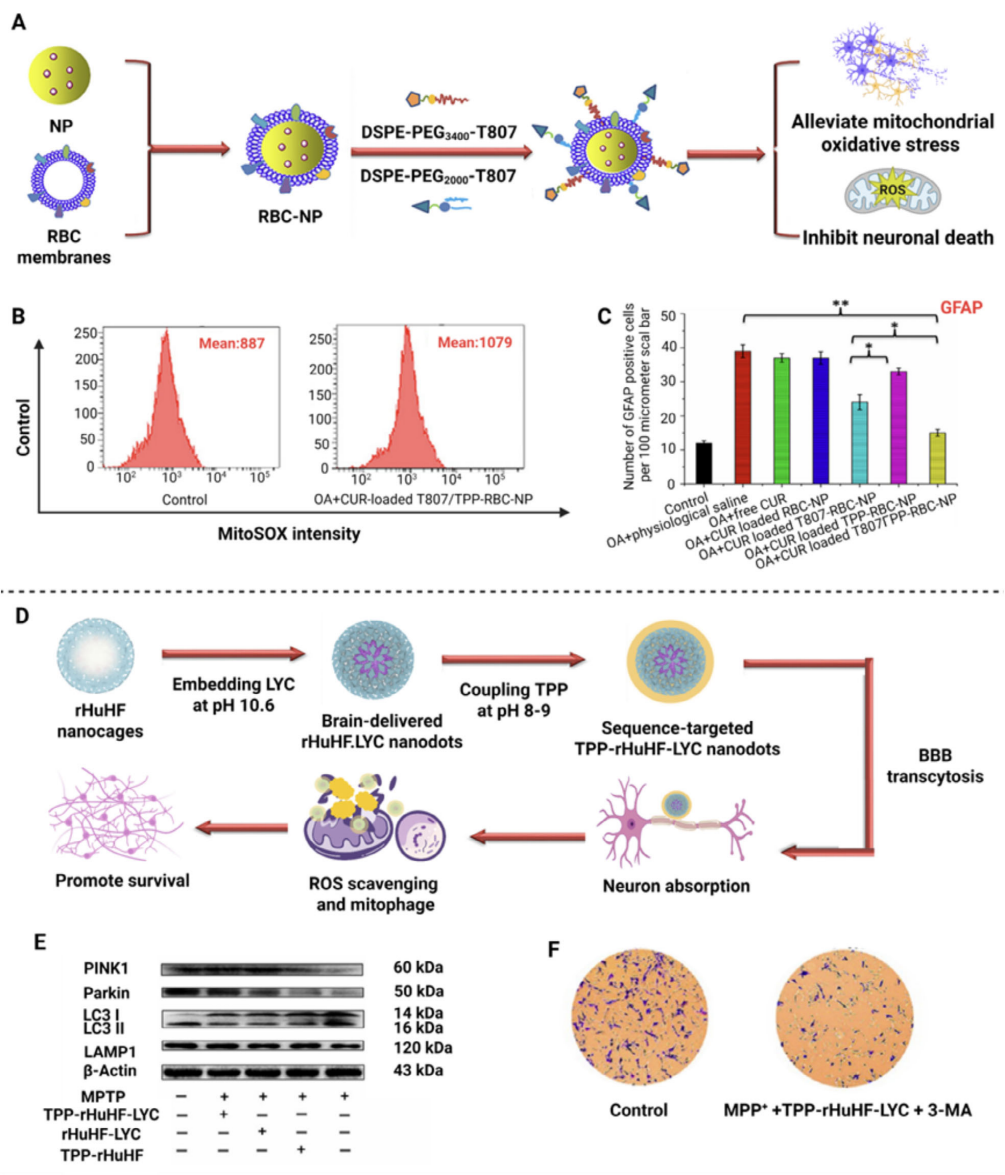
Engineering nanocarriers for targeted therapy of neurodegenerative diseases. A) Schematic illustration of the preparation and application of T807/TPP‐RBC‐NPs. The system features a human serum albumin nanoparticle core coated with a red blood cell membrane, functionalized with dual ligands. T807/TPP‐RBC‐NPs significantly alleviate mitochondrial oxidative stress and inhibit neuronal death. B) Cellular uptake of different coumarin‐labeled formulations by OA‐injured HT22 cells in a co‐culture model. C) Immunohistochemical staining results for GFAP. Reproduced with permission.^[^ ^113^ ^]^ Copyright 2020 Acta Materialia Inc. Published by Elsevier Ltd. All rights reserved. D) Schematic diagram of the preparation and mechanism of TPP‐rHuHF‐LYC nanodots. Lycopene is loaded into the nanocage cavity of recombinant human H‐ferritin (rHuHF), and TPP is conjugated to its outer surface to construct the sequentially targeted nanoparticle TPP‐rHuHF‐LYC, which significantly enhances mitophagy and promotes neuronal survival. E) Changes in the expression of proteins in the PINK1/Parkin mitophagy pathway in mouse dopaminergic neurons. F) Representative microscope images of cell morphology from the pro‐survival mitophagy experiment. Reproduced with permission.^[^ ^116^ ^]^ Copyright 2023, American Chemical Society. Created in https://BioRender.com.

Gao et al. proposed a biomimetic “outer shell + inner core” cascade targeting strategy. This strategy involves the use of human serum albumin nanoparticles as the core, coated with red blood cell membranes, and incorporating dual ligands T807 (promoting brain/neuronal targeting) and TPP (mitochondrial targeting) into the membrane exterior (T807/TPP‐RBC‐NPs). The system is loaded with curcumin, which enables triple‐tiered targeting (BBB→neuron→mitochondria). This significantly alleviates mitochondrial oxidative stress and inhibits neuronal death both in vitro and in vivo, thereby improving AD‐related phenotypes. These findings corroborate recent research and development (R&D) trends, specifically the integration of BBB transport peptides with mitochondrial targeting groups to construct multifunctional nanoplatforms achieving dual targeting^[^ ^113^ ^]^ (**Figure** **11**A–C). These platforms have the capacity to address multiple hallmark pathologies of AD, including β‐amyloid aggregation, oxidative stress, neuroinflammation, and mitochondrial dysfunction, through a singular formulation. In summary, the field of mitochondrial‐targeting nanomedicines for AD has evolved from a focus on simple antioxidant delivery, as exemplified by TPP‐CeO_2_ nanoenzymes, to a more sophisticated integration of strategies that target both the BBB and mitochondria. This evolution enables the concurrent delivery of therapeutic interventions, thereby offering a novel and promising paradigm for modifying the course of AD.

Mitochondrial dysfunction is a core driver of PD pathogenesis, manifested as impaired mitochondrial clearance due to defects in the electron transport chain (particularly complex I) and dysregulation of the PINK1/Parkin pathway. The aforementioned factors, when considered collectively, contribute to the characteristic loss of dopaminergic neurons in the substantia nigra and the accumulation of protein aggregates (α‐synuclein) in PD.^[^ ^260^ ^]^ Consequently, mitochondrial‐targeted nanotherapies for PD aim to simultaneously restore mitochondrial quantity (biogenesis) and quality (mitophagy), while protecting neurons from oxidative stress and proteotoxicity. Zheng et al. recently employed biomimetic copper selenide nanocarriers coated with macrophage membranes and modified with DSPE‐PEG‐TPP, loaded with curcumin.^[^ ^153^ ^]^ This mitochondria‐targeted nanomedicine (abbreviated as CSCCT nanoparticles) was engineered to migrate specifically to inflamed neural tissues, activating the NAD+/SIRT1/PGC‐1α/NRF1/TFAM signaling cascade to upregulate mitochondrial DNA replication and biogenesis. In mouse models of PD induced by MPTP, treatment with CSCCT nanoparticles led to a significant restoration of mitochondrial function. This restoration was characterized by a reduction in mitochondrial reactive oxygen species levels and an improvement in mitochondrial membrane potential. Additionally, treatment resulted in enhanced cellular respiration in dopaminergic neurons. Concurrently, enhanced mitochondrial autophagy for the selective clearance of dysfunctional mitochondria proved equally critical. In their study, Chen et al. engineered nanoparticles surface‐grafted with PINK1 antibodies, which were designed to recognize depolarized mitochondria. The researchers developed a simultaneous delivery system for siRNAs targeting USP30, a deubiquitinating enzyme that has been shown to antagonize Parkin‐mediated mitochondrial autophagy. This enhanced PINK1/Parkin‐mediated mitochondrial autophagy has been shown to promote both damaged mitochondrial clearance and healthy mitochondrial protection.^[^ ^260^ ^]^ Building upon this, more integrated “metabolism‐mitochondria‐inflammation” oriented work employs natural antioxidant molecules and achieves “BBB transport + dual targeting to mitochondria”. For instance, Xia et al. loaded lycopene into the nanocage cavity of recombinant human H‐ferritin (rHuHF) and conjugated TPP to its outer surface, constructing the sequence‐targeted TPP‐rHuHF‐LYC nanoparticle. This platform utilizes transferrin receptor‐mediated transcytosis across the BBB for brain entry while achieving mitochondrial localization via TPP. In the MPP^+^‐induced injury model, TPP‐rHuHF‐LYC significantly reduced intracellular Ca^2^⁺ overload and mitochondrial ROS (mitoSOX), restored ATP to 0.84 ± 0.02 (control = 1.00; MPP^+^ = 0.62 ± 0.01), and decreased NADH/NAD+ from 3.52 to 2.05. The drug also increased the LC3‐II/I ratio to 0.86 (MPP+ group = 0.50), up‐regulated PINK1/Parkin expression, and reduced α‐synuclein levels. In vivo, it demonstrated brain enrichment, improved polarity tests, and enhanced striatal dopamine levels, among other motor and biochemical indicators (Figure 11D–F).^[^ ^116^ ^]^

In addition to these “single‐target” interventions, researchers investigated combination therapies employing nanocarriers. For instance, nanoparticles loaded with antioxidants have been shown to inhibit α‐synuclein fibrillation and alleviate microglial inflammation, thus targeting two key drivers of PD progression.^[^ ^261^ ^]^ Concurrently, innovative delivery pathways and brain penetration methods are equally crucial. Guo et al. developed an inhalable cobalt‐doped Prussian blue nanozyme (PB/Co) encapsulated within DOTAP/DPPC cationic liposomes to form (PB/Co)@DD nasal spray. This formulation utilizes the olfactory bulb pathway to bypass the BBB and enter brain tissue. This nanozyme displays SOD/POD/CAT multi‐enzyme‐like activity, concomitantly scavenging existing ROS and inducing mitochondrial autophagy in the striatum to impede ROS regeneration at its origin. This approach has been demonstrated to reduce neuroinflammation and reverse motor deficits in models of PD, including MPTP and 6‐OHDA models.^[^ ^154^ ^]^ Collectively, these advances demonstrate multifaceted pathways for mitochondrial‐targeted nanomedicine intervention in PD, creating new mitochondria, eliminating defective mitochondria, delivering therapeutic genes and antioxidants, and precisely triggering drug release at target sites. This approach aims to achieve neuroprotection and metabolic restoration through a coordinated multi‐targeted strategy involving “energy metabolism, protein homeostasis, and neuroinflammation”.

In summary, the core strategy for treating brain metabolic disorders such as AD and PD with nanomedicines lies in constructing multifunctional nanoplatforms capable of crossing the BBB. This objective is realized through the surface modification of brain‐targeting peptides and mitochondrial‐targeting ligands, thus facilitating the precise delivery of therapeutic agents to neuronal mitochondria. These nanomedicines have been shown to scavenge reactive oxygen species in order to alleviate oxidative stress. In addition to this, they have been demonstrated to restore mitochondrial function and energy metabolism through multiple pathways, including the regulation of mitochondrial autophagy and the promotion of biogenesis. Concurrently, they intervene in multiple pathological pathways, including abnormal protein aggregation and neuroinflammation, thereby achieving comprehensive intervention across the entire disease network.

## 7 Current Status, Challenges, and Future Prospects

### 7.1 Clinical Research on Nanomedicines in Mitochondria‐Related Metabolic Diseases

Currently, nanomedicines have made substantial progress in their clinical translation to the treatment of metabolic diseases, including obesity, diabetes, atherosclerosis, and non‐alcoholic fatty liver disease. A synopsis of the clinical trial status of nanomedicines for treating metabolic diseases is shown in **Table** **5**. First, liposomes represent a primary nanocarrier for nanomedicines in clinical trials to target metabolic diseases. Due to their structural similarity to cell membranes, they exhibit excellent biocompatibility and biodegradability, significantly reducing immunogenicity and systemic toxicity. Extensive biosafety data of liposomes amassed over the course of decades of their clinical use suggests that they are a biosafe nanocarrier with the lowest risk from a regulatory point of view.^[^ ^262^ ^]^ Second, biomimetic nanomedicines have the potential to emerge as a future direction in the realm of nanomedicine development. In comparison to liposomes, biomimetic nanomedicines have the capacity to more accurately mimic natural components or structures in biological systems to improve their biocompatibility and functionality. For example, biomimetic nanomedicines display an excellent targeting capacity because they can actively recognize and bind to specific receptors or biomarkers on the surface of target cells by mimicking the recognition mechanisms of natural molecules in the body. In contrast, liposomes generally possess a relatively weak active targeting capability. Their surfaces are typically modified by attaching targeting molecules, including antibodies or ligands, to achieve a certain degree of active targeting.^[^ ^263^ ^]^ Third, the sample size of the existing clinical trials for nanomedicines is generally small, and there is insufficient data to build the correlation between nanomedicine efficacy and their mechanisms of action against metabolic diseases. A significant gap in the existing body of clinical trial research is on the direct regulatory impact of nanomedicines on mitochondrial dysfunction, including processes such as oxidative phosphorylation and kinetic imbalance.^[^ ^264^ ^]^

**Table 5:** Nanomedicines used in clinical trials.

Disease	Research topic	Research phase	Number of participants	Project number	Institution	Type of nanomedicine	Therapeutic drug
Obesity	Zein nanoparticles for glycemic control (glucocaps)	Not Applicable	69	Nct05560412	Clinica Universidad de Navarra	Organic nanoparticles	Zein
	Liposomal amphotericin B (ambisome) pharmacokinetics administrated with a single intravenous dose to obese patients (ASPEN)	Phase 4	16	NCT02320604	Radboud University Medical Center	Liposomes	Amphotericin B (ambisome)
	Effects of glutathione (an antioxidant) and N‐Acetylcysteine on inflammation	Not Applicable	78	NCT01550432	Stanford University	Liposomes	Glutathione, N‐acetylcysteine
Diabetes	Clinical application of mesenchymal stem cells seeded in a chitosan scaffold for diabetic foot ulcers	Phase 1	40	Nct03259217	Assiut University	Polymers	Mesenchymal stem cells
	Capsaicin nanoparticles in patients with painful diabetic neuropathy	Phase 2 Phase 3	60	NCT01125215	Mahidol University	Organic nanoparticles	Capsaicin
Atherosclerosis	Treatment of patients with atherosclerotic disease by methotrexate‐incorporated LDL‐like nanoparticles	Phase 2 Phase 3	40	Nct04616872	University of Sao Paulo General Hospital	Biomimetic nanoparticles	Methotrexate
	Treatment of patients with atherosclerotic disease with paclitaxel‐incorporated LDL‐like nanoparticles (PAC‐MAN)	Phase 2 Phase 3	40	NCT04148833	University of Sao Paulo General Hospital	Biomimetic nanoparticles	Paclitaxel
	Alprostadil liposomes for injection for lower extremity arteriosclerosis obliterans	Phase 2	20	NCT04197323	CSPC Zhongqi Pharmaceutical Technology Co., Ltd	Liposomes	Alprostadil
	A proof‐of‐concept study on determining local delivery efficiency and efficacy of a nanocort (DELIVER)	Phase 1 Phase 2	21	NCT01647685	Academisch Medisch Centrum‐Universiteit van Amsterdam	Liposomes	Prednisolone sodium phosphate
	Silencing inflammatory activity by injecting a nanocort into patients with atherosclerotic disease (SILENCE)	Phase 1 Phase 2	30	NCT01601106	Academisch Medisch Centrum‐Universiteit van Amsterdam	Liposomes	Prednisolone sodium phosphate
	Prostaglandin E1 (Liprostin) treatment with lower limb angioplasty for peripheral arterial occlusive disease	Phase 2	12	NCT00053716	–	Liposomes	Prostaglandin E1
	Plasmonic nanophotothermal therapy of atherosclerosis (NANOM‐FIM)	Not Applicable	180	NCT01270139	Ural State Medical University	Inorganic nanoparticles	Silicon dioxide gold nanoparticles
NAFLD	The effect of curcumin on the development of prednisolone‐induced hepatic insulin resistance (curpred)	Not Applicable	24	Nct04315350	Copenhagen University Hospital	Liposomes	Curcumin

The safety and preliminary efficacy of nanomedicines in the treatment of metabolic diseases have been confirmed from current clinical studies, particularly in anti‐inflammatory interventions for atherosclerosis and the management of diabetes complications. However, precision nanotherapy targeting mitochondrial dysfunction remains in the preclinical stage. Future effort should be dedicated into two key directions, the development of mitochondria‐targeting clinical nanoformulations to overcome subcellular delivery barriers; and the execution of Phase II/III trials based on mitochondrial pathophysiological mechanisms, advancing from “tissue repair” to “organelle function reconstruction”.^[^ ^265^ ^]^

### 7.2 Challenges of Nanomedicines for Mitochondria‐Related Metabolic Diseases

**Figure 12:**
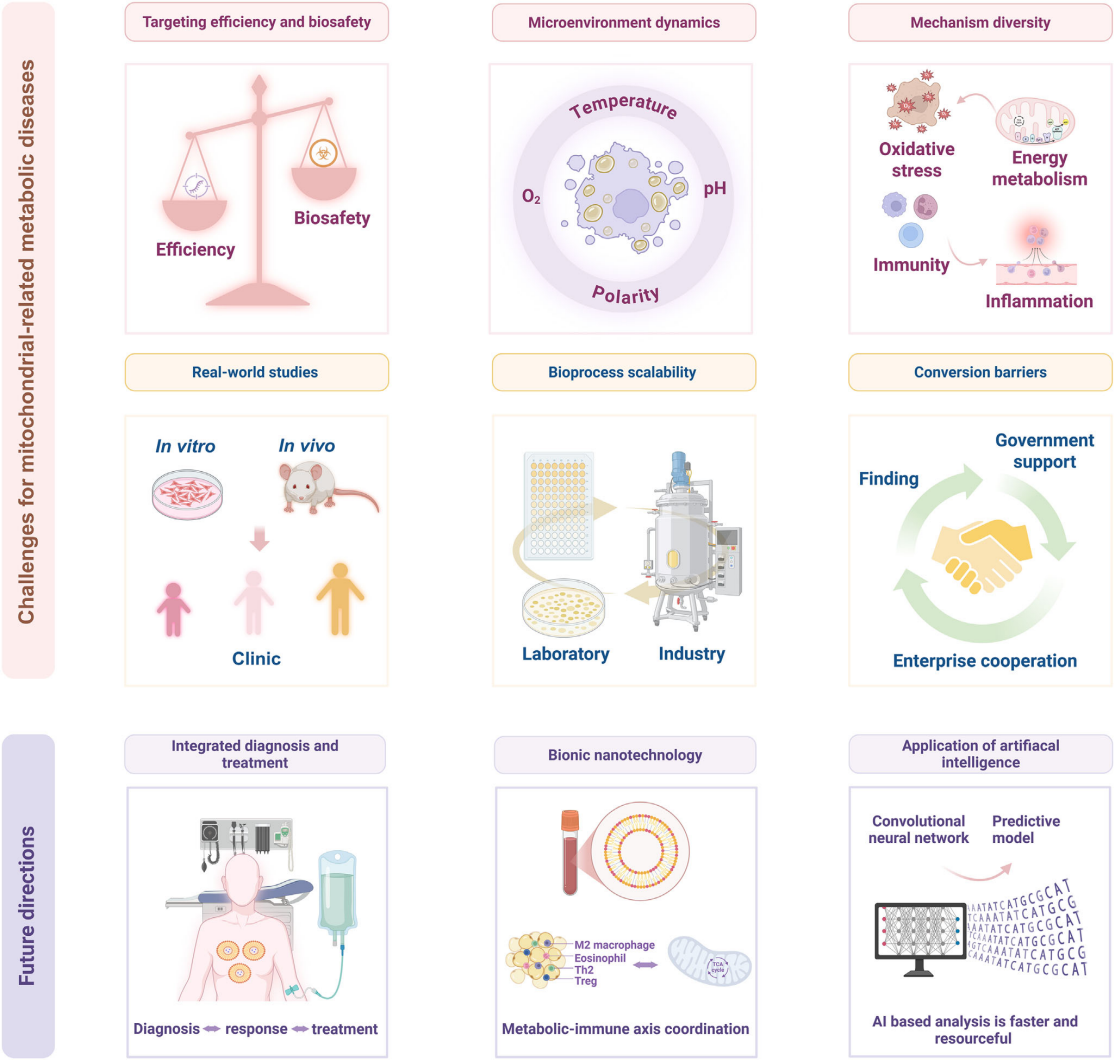
Challenges and future directions of nanomedicines for mitochondria‐related metabolic diseases. Challenges including targeting efficiency, diverse mechanisms & synergistic intervention, limited data for nanomedicine developability by weighing the targeting efficiency and biosafety, bioprocess scalability & analytical standardization, and conversion barriers. Future directions include developing integrated diagnosis‐treatment‐control smart nanosystems, coordination of the metabolism‐immune axis & bionic nanotechnology, and AI‐driven personalized therapy. Created in https://BioRender.com.

Mitochondria‐targeting nanomedicines have demonstrated their potential in the treatment of metabolic diseases, but their clinical translation faces multidimensional and multi‐scale systemic challenges.^[^ ^266^ ^]^ The nanocarriers for passive targeting of mitochondria are often designed on the basis of the mitochondrial membrane potential (ΔΨm), while targeting of these nanocarriers may fail under pathological conditions. Multi‐pathway synergistic mechanisms of antioxidation, metabolic regulation, and immune regulation have not been comprehensively elucidated. Additionally, biocompatibility, batch consistency, and regulatory framework compliance of nanocarriers for nanomedicines remain key obstacles to their clinical translation. Current research challenges are summarized in **Figure** **12**. These challenges include a trade‐off between the targeting efficiency and biosafety of nanomedicine, the complexity of a pathological microenvironment and the challenge of its dynamic evolution through different stages of diseases, synergistic challenges and “double‐edged sword” effects of multi‐dimensional pathological interventions, large‐scale production in a good manufacture practice (GMP) facility versus tailored production for personalized individual treatment, systemic bottlenecks in clinical translation, and real‐time, in situ assessment of therapeutic efficacy.^[^ ^264^, ^265^, ^267^ ^]^ In the following sections, molecular mechanisms, technical bottlenecks, and translation barriers of these challenges are systematically analyzed to lay the scientific foundation for the development of breakthrough strategies.

The core challenge of mitochondria‐targeting nanomedicines lies in an optimized balance between the targeting efficiency and biosafety of the nanocarrier system.^[^ ^265^ ^]^ Currently, the mainstream mitochondrial targeting strategies, such as TPP, rely on the membrane potential to realize targeting. Under pathological conditions, such as a reduced mitochondrial membrane potential or a damaged membrane structure, these strategies may fail, or even lead to mitochondrial depolarization due to excessive positive charges that triggers cell apoptosis.^[^ ^268^ ^]^ For example, gold nanomedicines or liposomal delivery systems may exhibit off‐target effects in tumor or neurodegenerative disease models, and they accumulate non‐specifically in the liver or kidneys to exacerbate their systemic toxicity.^[^ ^269^ ^]^ In addition, long‐term biocompatibility of nanocarriers, especially inorganic nanoparticles, remains to be systematically evaluated. For example, although cerium oxide nanomedicines can clear ROS through the Ce^3^⁺/Ce⁴⁺ cycle, their retention in the body may cause chronic inflammation or tissue fibrosis.^[^ ^121^ ^]^

Dynamic changes in a disease microenvironment (e.g., pH, oxygen partial pressure, or ion concentrations) may be accommodated through tuning drug release kinetics.^[^ ^267^, ^270^ ^]^ Pathological changes in different disease states (e.g., vascular remodeling in late‐stage adipose tissue, narrowing of hepatic sinusoids in NASH, and plaque heterogeneity) and in individual patients can significantly impact the efficiency of passive and active targeting of nanomedicines.^[^ ^271^ ^]^ Additionally, the disease microenvironment (e.g., tumor hypoxia and chronic inflammation) can be primarily considered during the design of environment‐adaptive intelligent nanomedicines, while the intracellular mitochondrial microenvironment has not been adequately considered for nanomedicine design. Factors including actual temperature, polarity, hypoxia, and pH within the mitochondria are rarely considered in the development of mitochondria‐targeting nanomedicines. How these factors influence the targeting efficiency of a nanomedicine and its drug release remains to be elucidated. For instance, pH‐responsive nanodrugs, after escaping from lysosomes, may fail to trigger drug release due to an alkaline environment of the mitochondrial matrix, resulting in limited therapeutic efficacy.^[^ ^272^, ^273^, ^274^ ^]^

Although mitochondrial oxidative stress is a prevalent root cause for metabolic diseases, reliance on the antioxidation strategy (e.g., ROS scavenging or SOD mimicry) is inadequate for addressing disease‐specific pathological issues.^[^ ^275^ ^]^ For instance, in NAFLD, the contradictory mechanisms of compensatory enhancement of mitochondrial β‐oxidation and lipid toxicity‐induced ROS bursts suggest that combined regulation of lipid metabolism by nanomedicines should be implemented. In the context of diabetic cardiomyopathy, the observed damage to mitochondria and their accumulation are a consequence of defective mitophagy. A targeted approach should be developed to repair the PINK1/Parkin pathway.^[^ ^276^ ^]^

Another significant impediment to the transition from laboratory research to clinical application is stemmed from large‐scale production and analytical standardization of nanomedicines. Synthesis of nanomedicines in the laboratory is often at a gram level; however, industrial‐scale manufacturing frequently encounters issues such as discrepancies in particle size distribution and surface modification homogeneity from batch to batch. For instance, minor variations in process parameters (e.g., ultrasonic power and phospholipid ratios) during liposome surface modification with TPP can substantially impact the mitochondrial targeting efficiency.^[^ ^14^ ^]^ In addition, in GMP‐compliant production, the stability of the Ce^3^⁺/Ce⁴⁺ ratio in cerium oxide nanoparticles is critical by preventing ligand detachment from the surface of gold nanoparticles. This ratio must be controlled through a quality control system compliant with ISO 13485 standards.^[^ ^15^ ^]^

Moreover, clinical translation of nanomedicines must surmount policy and funding barriers. For instance, the FDA's nanomedicine classification guidelines (2022) stipulate a separate submission of the CMC data for carrier components. However, a GMP‐grade excipient is often in short supply, such as DSPE‐PEG2000.^[^ ^17^, ^277^ ^]^ Funding allocation is also imbalanced. Basic research on mitochondrial targeting mechanisms accounts for over 70% of the total funding, while translational research, such as formulation process optimization and toxicological evaluation, often relies on corporate investment.^[^ ^278^ ^]^

The majority of clinical data for nanomedicines are derived from Phase III clinical trials that are meticulously controlled through highly standardized trial designs (e.g., exclusion of concomitant medications and restriction of patient heterogeneity). These designs may result in significant discrepancies in the efficacy of nanomedicines between clinical studies and clinical practice. For instance, the combination of proton pump inhibitors (PPIs) and pH‐responsive nanocarriers has been demonstrated to substantially alter the drug release behavior. When a gastric pH increases from 1.2 to 6.0 due to co‐administration of PPIs, the intestinal release rate from Eudragit L series nanocarriers decreases from 82% to 47%.^[^ ^279^ ^]^ This pH‐dependent release discrepancy may result in inadequate drug exposure at the target site, consequently affecting therapeutic efficacy. In a similar manner, physiological characteristics of obese patients (e.g., a 3–5 cm thickness of the subcutaneous fat layer) may significantly alter the diffusion kinetics of locally injected nanomedicines. This phenomenon may be attributed to a high viscosity and a low vascular density of adipose tissue.^[^ ^280^ ^]^ However, leveraging Real‐World Research (RWS) to address nanomedicine developability challenges—such as batch‐to‐batch variability in carrier synthesis, unpredictable in vivo behavior under physiological heterogeneity, and long‐term biocompatibility—remains nascent.^[^ ^281^ ^]^ Establishing dedicated RWS frameworks (e.g., integrating electronic health records with nanocarrier pharmacokinetic data) requires significant funding to standardize data collection, validate analytical methods, and build predictive models for clinical translation. Current underinvestment in this field impedes solutions to critical scalability and safety barriers.^[^ ^282^ ^]^

### 7.3 Future Directions of Nanomedicines for Mitochondria‐Related Metabolic Diseases

We project three directions for the development of nanomedicines for the regulation of mitochondria. First, the integration of targeted delivery, real‐time imaging, and enhanced therapeutic action into one single nanocarrier could achieve “visualized precision/potent interventions”.^[^ ^283^, ^284^ ^]^ The advent of multimodal imaging technologies, such as PET/MRI dual‐modality probes, has enabled the tracking of the spatiotemporal distribution of nanomedicines in myocardial tissues, thereby ushering in a novel medical paradigm of “visualized therapy”.^[^ ^285^, ^286^, ^287^ ^]^

Second, the construction of nanomedicines for multidimensional regulation of the metabolic‐immune axis could realize simultaneous metabolic remodeling and immune regulation. To achieve synergistic regulation of the metabolic‐immune axis, the core resolution lies in breaking the malicious cycle of mitochondrial‐immune interactions. For instance, nanodrugs designed to target the mitochondria of macrophages have been shown to restore the ATP/ADP ratio, promote M2 polarization, and reverse chronic inflammation. Similarly, a novel “nanoblume” after integration of an enzyme, Mn_3_O_4_‐IRF‐5siRNA, has demonstrated the capacity to catalyze the clearance of ROS and reprogram the macrophage phenotype, resulting in a 48.7% reduction in neural scarring after spinal cord injury.^[^ ^288^, ^289^ ^]^ Such multi‐mechanism synergistic strategies have the potential to overcome the limitations of metabolic‐immune cross‐regulation.

Third, artificial intelligence‐driven personalized therapy can be employed by leveraging multi‐omics data and intelligent algorithms to achieve end‐to‐end customization from drug design and efficacy prediction to dynamic dose optimization (Figure 12). Collaborative bioinformatics tools have been instrumental for advancing mitochondrial physiology and pathology research, and these tools are developed from intertwined advances in high‐throughput screening, genome‐wide analysis, and other cutting‐edge technologies.^[^ ^290^ ^]^ The development of personalized nanodrugs tailored to the patients’ genotypes, metabolomics, and mitochondrial surface characteristics is poised to become the prevailing paradigm in this field. For instance, natural phenolic acid derivatives, such as MITO‐rosmarinic acid, can be efficiently customized through continuous flow synthesis, and their mitochondrial targeting efficiency and antioxidant activity can be tailored according to individual differences.^[^ ^291^ ^]^ Additionally, artificial intelligence (AI) has emerged as a pivotal preclinical research instrument in nanomedicines. The construction of functional groups in nanodrugs could be accelerated through AI models for drug‐nanocarrier linkers, their 3D conformation, and nano‐bio interactions. These AI models can be integrated to predict delivery, distribution, and mechanisms of action of nanomedicines.^[^ ^292^ ^]^

Four, in addition to the preceding strategies, mitochondrial transplantation (MT) can serve as a parallel and complementary approach; its core rationale is the direct supplementation of functional mitochondria to restore impaired cellular bioenergetics and re‐establish organ homeostasis.^[^ ^293^ ^]^ Preclinical evidence indicates that MT reduces hepatic lipid deposition, enhances energy metabolism, and improves insulin responsiveness in models of fatty liver disease and metabolic syndrome, consistent with a “multi‐tissue benefit” profile.^[^ ^294^, ^295^ ^]^ Stem cell–mediated mitochondrial transfer provides mechanistic and therapeutic support for metabolic liver disease,^[^ ^296^ ^]^ and when combined with exogenous mitochondrial pretreatment, further augments hypoglycemic and hepatoprotective effects in T2D‐NAFLD.^[^ ^297^ ^]^ At the level of complications, platelet‐derived mitochondrial transplantation has been shown to ameliorate cognitive impairment and improve brain mitochondrial function in diabetic mice, suggesting that systemic metabolic abnormalities and their neurological sequelae may also benefit.^[^ ^298^ ^]^ Notably, while MT itself constitutes an organelle‐replacement therapy rather than a nanomedicine modality, nanotechnology could plausibly optimize MT—for example, by enabling targeted delivery, protective encapsulation, and functional modification of mitochondria—thereby achieving synergy.

Five, restoring lysosomal function is emerging as a novel strategy for treating mitochondrial dysfunction in metabolic diseases. The acidic environment of lysosomes can be rewired using nanomaterials to effectively restore autophagic flux and improve mitochondrial function in hepatocytes and pancreatic β‐cells. This establishes the “lysosomal reset‐mitochondrial recovery” therapeutic logic.^[^ ^299^, ^300^ ^]^ This process involves the lysosomal Ca^2^⁺‐TFEB signalling axis, a pathway that links lysosomal function directly to mitochondrial quality control.^[^ ^301^ ^]^ Furthermore, enhanced lysosomal acidification alleviates cellular iron deficiency and indirectly corrects mitochondrial respiratory defects.^[^ ^302^ ^]^ In liver disease models, enhanced lysosomal biogenesis has also been shown to improve mitochondrial bioenergetics synergistically.^[^ ^303^ ^]^ Future studies combining “lysosomal reacidification” nanomedicine interventions with mitochondrial‐targeting strategies, validated through stratified analysis in NAFLD/T2D populations, show promise in achieving precise metabolic regulation from the level of individual organs to the whole organism.

Moreover, advances in nanomedicines for metabolic diseases generally require multidisciplinary collaboration among the fields of pharmacology, bioinformatics, clinical medicine, materials science, nanotechnology, artificial intelligence, and systems biology. A substantial body of research has validated the importance of interdisciplinary collaboration on applying cutting‐edge technologies/scientific discoveries from different disciplines.^[^ ^304^ ^]^ The treatment of metabolic diseases is undergoing a paradigm shift from a single‐target intervention model to a multidimensional dynamic regulation model through interdisciplinary collaboration and innovation. This transition is expected to achieve precise control of metabolic diseases through the entire nanomedicine life cycle, from its discovery and development to its in vivo behavior prediction, efficacy assessment, and personalized application.^[^ ^305^ ^]^ The ultimate objective is to establish a spatiotemporal dynamic regulation nanosystem for mitochondrial repair, thereby driving the evolution of metabolic disease treatment from molecular intervention to systemic regulation.

## 8 Summary

Traditional drugs face challenges in precisely intervening in mitochondrial function including poor targeting, low bioavailability, and adverse side effects. Nanomedicine provides a novel approach for enhancing mitochondrial function through the application of engineered design, multimodal synergy, and microenvironment‐responsive strategies. Design, manufacturing, and translation of nanomedicine are contingent on interdisciplinary collaboration and integration of advanced technologies such as multiomics, computational modeling, and artificial intelligence. Advances in these technologies enhance our fundamental understanding of the interaction between nanomedicine and biological response and support the development of safe, targeted, effective, multi‐drug‐loading, and multi‐modular nanomedicine for the treatment of metabolic diseases. The overarching objective is to formulate “organ‐cell‐mitochondria” multi‐level targeted repair strategies. Future research endeavors should prioritize the solutions to prevailing impediments in the clinical translation process. This transition could lead to precise, efficient, and safe clinical practices that can effectively address and potentially reverse the pathological progression of major metabolic diseases, including obesity, diabetes, NAFLD, and atherosclerosis.

## Conflict of Interest

The authors declare no conflict of interest.
